# Pancreatic α-cells are functionally heterogeneous and sex-dependently regulated by neighboring endocrine cells

**DOI:** 10.1038/s42003-026-10163-6

**Published:** 2026-06-23

**Authors:** Ya-Chi Huang, Marjan Slak Rupnik, Guy A. Rutter

**Affiliations:** 1 Research Centre of the Hospital Centre of the University of Montreal (CRCHUM) and University of Montreal, Montreal, QC Canada; 2 Centre for Physiology and Pharmacology and Comprehensive Center for AI in Medicine, Medical University of Vienna, Vienna, Austria; 3 Institute of Physiology, Faculty of Medicine, University of Maribor, Maribor, Slovenia; 4 Alma Mater Europaea University Maribor, Maribor, Slovenia; 5 Section of Cell Biology and Functional Genomics, Division of Diabetes, Endocrinology and Metabolism, Department of Metabolism, Digestion and Reproduction, Faculty of Medicine, Imperial College London, Hammersmith Hospital, London, UK; 6 Research Institute of the McGill University Health Centre, Montreal, QC Canada; 7 Lee Kong Chian School of Medicine, Nanyang Technological College, Nanyang, Singapore

**Keywords:** Diabetes, Diabetes, Calcium signalling

## Abstract

Glucose and paracrine regulation of α-cells, particularly with respect to sex differences, is still poorly understood. Here, we imaged islets from GluCre:GCaMP6f mice in pancreatic slices, additionally loaded with a red Ca²⁺ indicator, to precisely interrogate Ca²⁺ dynamics in α-cells and the adjacent β- and δ-cells. During a glucose ramp (1.8-10.8 mM), α-cell Ca²⁺ oscillations were heterogeneous, antiphasic to β-cells, and inversely correlated with δ-cells. Selective inhibition of insulin receptor (InsR) and somatostatin receptor subtypes-2 and 3 (SSTR2/3) shifted the majority of α-cells to hyperactivity, delayed the decline of α-cell activity and onset of β-cell Ca²⁺ oscillations during the ramp. While female α-cells exhibited greater sensitivity to SSTR2/3 inhibition, male α-cells were more responsive to InsR blockade. Under complete SSTR2/3 and InsR antagonism, α-cells exhibited elevated Ca²⁺ oscillations but remained glucose-dependent. We conclude that intra-islet coordination fine-tunes α-cell glucose responses, with sex-specific paracrine signaling potentially shaping glucagon profiles in metabolic disease.

## Abstract (web summary)

High spatiotemporal resolution imaging of pancreatic slices from GluCre:GCaMP6f mice reveals Ca²⁺ dynamics in α-cells and neighboring β- and δ-cells, uncovering sex-dependent differences in α-cell responses to insulin and somatostatin.

## Introduction

Glucagon, secreted by pancreatic α-cells, is a key hormone that counteracts the actions of insulin to prevent hypoglycemia. Under physiological conditions, glucagon secretion is potentiated at low glucose ( < 5 mM) and suppressed at high glucose ( > 5-7 mM)^1^. The tight regulation of α-cell glucagon secretion, together with the coordinated control of insulin and somatostatin release from the neighboring β- and δ-cells respectively, is essential for maintaining normoglycemia. In diabetes, however, the regulation of α-cell activity is impaired^2^. Thus, in type 2 diabetes (T2D), α-cells hyper-secrete glucagon, leading to excessive hepatic glucose production and worsening hyperglycemia. Conversely, in type 1 diabetes (T1D), α-cell glucose sensing mechanisms are compromised. Patients with T1D consequently experience postprandial hyperglycemia and face the life-threatening risk of hypoglycemia during periods of fasting.

Despite its critical role in glucose homeostasis, the regulation of α-cell glucagon secretion is incompletely understood. It is, for example, unresolved whether α-cells sense glucose directly^1,3–6^, or indirectly^7–13^ via paracrine signaling, to regulate glucagon secretion. At a more fundamental level, it is debated whether rising glucose levels stimulate^14^, suppress^15,16^, or do not affect^17^ glucagon secretion from α-cells. A recent study employing α-cell-specific GCaMP-expressing mouse models demonstrated heterogeneous patterns of glucose-stimulated cytosolic Ca^2+^ responses and glucagon secretion across α-cell subpopulations^18^. This aligns with our previous findings^5^, as well as those of others^19^, demonstrating that pancreatic α-cells exhibit both functional^5,19,20^ and genetic heterogeneity^19,21^. However, the mechanisms and physiological relevance of this heterogeneity remain unclear, particularly in the context of disrupted intra-islet signaling.

α-, β-, and δ-cells are closely positioned within rodent and human islets^22^. This anatomical juxtaposition is believed to support local feedback loops that fine-tune islet hormone secretion and glucose regulation^23^. Indeed, the suppressive actions of β-, and δ-cells on α-cell activity are well established; β-cells secrete insulin, which suppresses α-cell activity via insulin receptor signaling^11,13,24^, and urocortin 3, which regulates δ-cell activity^25^, while δ-cells release somatostatin, which inhibits glucagon release by acting on somatostatin receptors^8,10,12,13,26^. Given that a rise in intracellular Ca²⁺ is the primary trigger for hormone release in α-, β-, and δ-cells^5,27,28^, approaches that simultaneously monitoring dynamic Ca²⁺ oscillations within these cells offer a convenient proxy for assessing hormone secretion at the single-cell level, particularly when examining interactions between neighboring cells, such as β- and δ-cells, which are anatomically adjacent to α-cells and are likely to exert regulatory influences.

Men and women differ in their susceptibility to developing T1D^29^ and T2D^30^. While significant sex differences in glucose metabolism, glycemic control, and insulin sensitivity have prompted substantial research into sex-dependent β-cell function in diabetes^31,32^, data on potential heterogeneity of α-cell function from a sex-dimorphic perspective are unavailable. Given that sex-specific mechanisms in islet function may underlie differential susceptibility and metabolic outcomes in disease^33^, advancing our understanding of sexually-dimorphic α-cell functions holds considerable potential for developing more effective and potentially sex-specific strategies to correct hyperglucagonemia in patients with diabetes.

In the present study, we employed both sex of age-matched reporter mice expressing the genetically-coded Ca^2+^ sensor GCaMP6f selectively in α-cells^34^. High spatiotemporal resolution dual-Ca²⁺ indicator confocal imaging was performed in fresh pancreas slices, wherein a red-shifted Ca^2+^ indicator was further loaded. This approach enabled simultaneous monitoring of the Ca^2+^ oscillations in α-, β- and δ-cells. We aimed to investigate the α-cell heterogeneity through 1) characterizing, at the single-cell level, α-cell Ca^2+^ oscillation patterns in response to glucose; 2) assessing the Ca^2+^ oscillation patterns in α-cells in relation to the activities of neighboring β- and δ-cells. By selectively blocking insulin and somatostatin signaling pathways, we evaluated whether glucose regulates α-cell glucagon secretion via intrinsic glucose sensing mechanisms, paracrine interactions, or both; 3) lastly, addressing the contribution of sex dimorphism to α-cell glucose responsiveness with or without perturbed paracrine signaling.

## Results

### In vivo characterization of the GluCre: GCaMP6f mouse

**Fig. 1:**
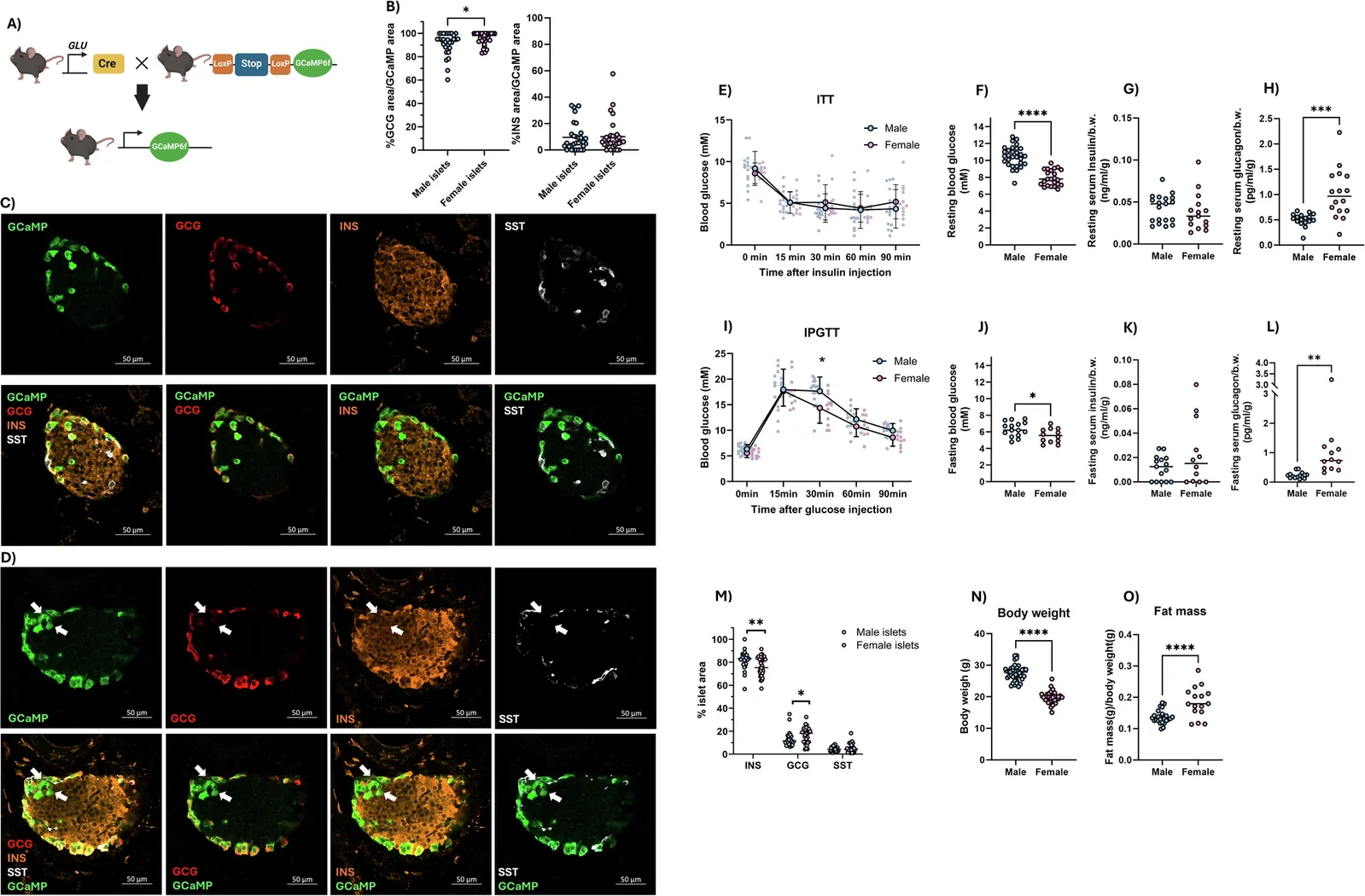
Characterization of GluCre:GCaMP6f mice. **A** Schematic diagram of GluCre:GCaMP6f mouse generation. Homozygous GluCre mice were crossed with homozygous GCaMP6f^fl/fl^ mice, in which GCaMP6f is expressed downstream of a LoxP-flanked STOP cassette. Created in BioRender. Rutter, G. (2026) https://BioRender.com/v0q30i9. **B** Proportions of glucagon-positive (GCG, left) and insulin-positive (INS, right) areas within the total GCaMP-expressing area in individual islets from male (blue) and female (pink) mice. *N* = 30 islets from 6 males (76 ± 9.3 days), and 31 islets from 5 females (85.8 ± 7.8 days). *p < 0.05, unpaired two-tailed t-test. **C**, **D** Representative confocal images of male GluCre:GCaMP6f islets showing GCaMP expression (green) and immunostaining for glucagon (GCG, red), insulin (INS, orange), and somatostatin (SST, white). Arrows in (**D**) indicate ectopic GCaMP expression in β-cells. **E** Insulin tolerance test (ITT). *N *= 15 males and 12 females (80.3 ± 12 and 82.8 ± 13.4 days, respectively). Error bars represent mean ± standard deviation (SD). **F** Resting blood glucose levels. *N* = 30 males and 23 females (80.1 ± 1.2 and 85.8 ± 0.9 days). **G**, **H** Resting serum insulin (**G**) and glucagon (**H**) levels. *N* = 15 males and 12 females (79.3 ± 11.9 and 80.8 ± 13.4 days). ***p < 0.001, unpaired two-tailed t-test. **I** Intraperitoneal glucose tolerance test (IPGTT). *N* = 15 males and 12 females (84.3 ± 12 and 85.8 ± 13.4 days). *p < 0.05, two-way repeated measures ANOVA. Error bars represent mean ± SD. **J** Fasting blood glucose levels. *N* = 15 males and 12 females (74.7 ± 8.2 and 82.7 ± 12.3 days). *p < 0.05, unpaired two-tailed t-test. **K–L** Fasting serum insulin (**I**) and glucagon (**J**) levels. *N* = 15 male and 12 female mice with mean age: 74.7 ± 8.2 and 82.7 ± 12.3 days, respectively. **p < 0.01, unpaired two-tailed t-test. **M** Quantification of INS-, GCG-, and SST-positive areas relative to total islet area. *N* = 30 islets (6 males, 76 ± 9.3 days) and 31 islets (5 females, 85.8 ± 7.8 days). *p < 0.05, **p < 0.01, Mann–Whitney test. **N**, **O** Phenotypic measurements including body weight (**N**) and fat mass (**O**) in 25 males and 16 females (86.2 ± 12.6 and 85.0 ± 13.8 days). ****p < 0.0001, unpaired two-tailed t-test. All animal age were provided as mean ± SD.

In this study, we aimed to uncover the dynamic behavior of α-cell Ca^2+^ activity across extensive cell populations through large-scale, high-resolution analysis. To this end, we generated GluCre:GCaMP6f mice which expressed GCaMP6f, a Ca^2+^ sensor encoded downstream of the glucagon promoter, enabling GCaMP6f expression selectively in α-cells (Fig. 1A). Recombination in this model was highly efficient (Fig. 1B), and quantitative immunocytochemical analysis revealed strong colocalization between GCaMP6f and glucagon-positive areas of the islet (Fig. 1B–D); female GluCre:GCaMP6f mouse islets showed a greater degree of overlap between the glucagon positive areas and the GCaMP6f signal, compared to male islets (Fig. 1B left and 1C; female vs. male: 90.4 ± 2.24% vs. 87.4 ± 2.58% of GCaMP6f areas are positive for glucagon). The remaining 10.1% ± 2.21% and 9.52% ± 1.93% of total GCaMP6f region in female and male islets, respectively, were positive for insulin, suggesting minor mis-expression of GCaMP6f in β-cells (Fig. 1B right and 1D white arrows), as observed in a previous report^35^. For the present study, only cells that were positive for both GCaMP6 and glucagon were analyzed as α-cells.

Insulin sensitivity (Fig. 1E) and circulating fasting and resting insulin levels (Fig. 1G, K) in GluCre:GCaMP6f mice were comparable between male and female mice. However, the overall insulin-positive area within islets was smaller in female GluCre:GCaMP6f mice (Fig. 1M), suggesting that β-cells in females exhibit greater secretory efficiency than those in males, thereby enabling comparable circulating insulin levels. Under an intraperitoneal glucose tolerance test (IPGTT), female mice exhibited better glucose clearance than males (Fig. 1I). Female GluCre:GCaMP6f mice also showed lower fasting and resting glycemia (Fig. 1F, J), lower body weight (Fig. 1N), and higher body fat mass (Fig. 1O), when compared to their age-matched male counterparts. Female mice have higher circulating resting and fasting glucagon levels (Fig. 1H, L), likely resulting from the larger glucagon-positive regions in female islets (Fig. 1M).

### Glucose-stimulated Ca^2+^ dynamics in α-cells are heterogeneous

**Fig. 2:**
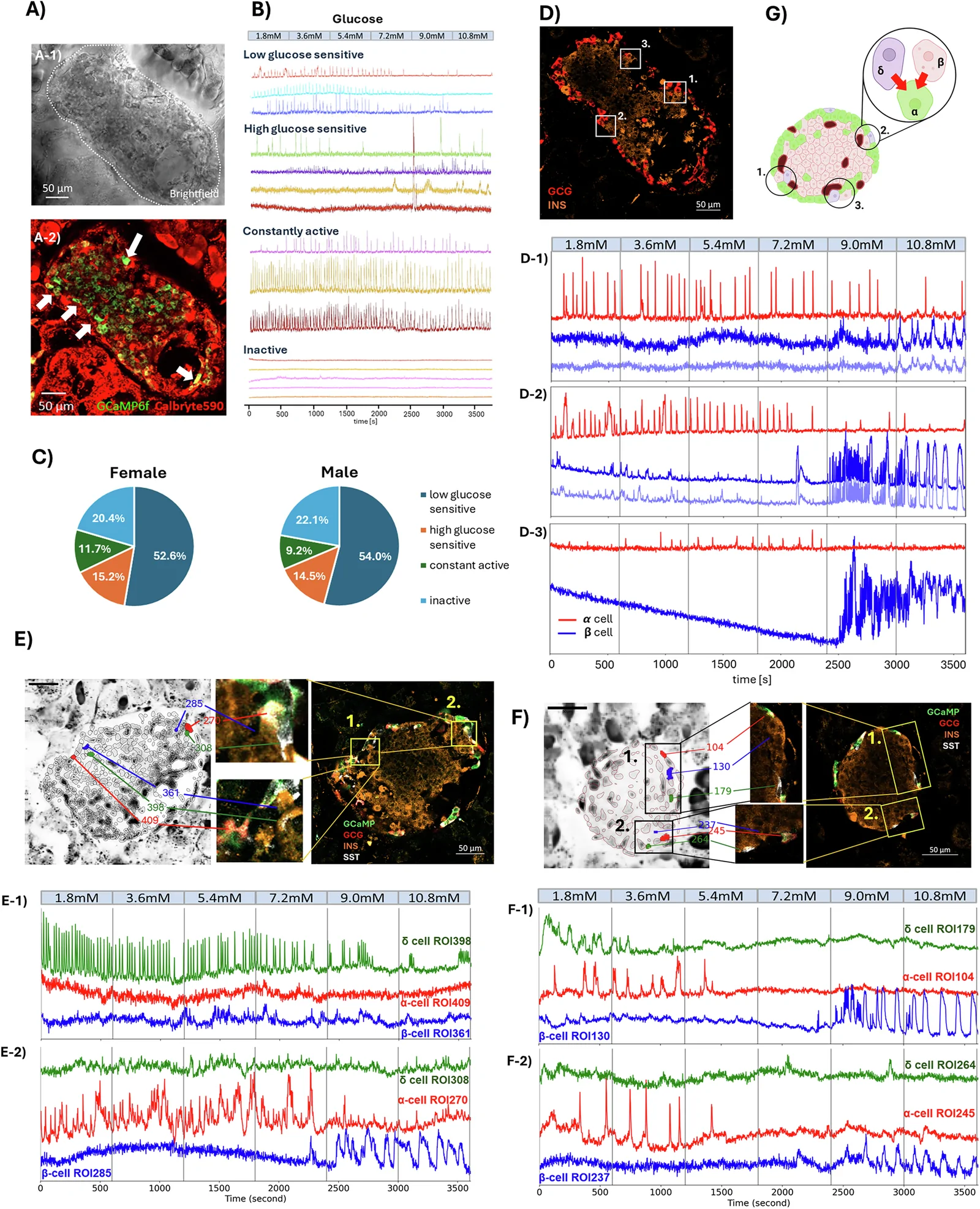
Ca^2+^ dynamics in α-, β- and δ-cells in GluCre:GCaMP6f islets in pancreas slice. **A** A representative islet within a pancreas slice. **A-1** Brightfield image of an islet (outlined by a white dotted line) in a pancreas slice prepared from a G*luCre:GCaMP6f* mouse. **A-2** All pancreatic cells in the slice are loaded with Calbryte 590^AM^ (red), while α-cells express GCaMP6f (dense bright green; some examples are indicated by white arrows). Note the lighter green fluorescence in cells at the islet center represents inert signals, stemming from background fluorescence and no Ca^2+^ dynamics has emanated from these signals. **B** The pancreas slice shown in (**A**) was perifused with an incremental glucose concentration ramp (top bar). Representative Ca²⁺ activity traces from α-cells in response to the glucose ramp are shown. These responses can be categorized into four groups: low glucose-sensitive, high glucose-sensitive, constantly active, and inactive α-cells. **C** Pie chart representations showing the proportions of types of α-cell response in islets in female (left) and male (right) mice. *N* = 47 male and 29 female islets collected from pancreas slices of 9 male and 5 female mice. Each region of interest (ROI) co-expresses GCaMP and glucagon. Every ROI is categorized according to its response to the incrementing glucose concentration ramp. The number of ROIs in each category was then normalized to the total number of ROIs in each islet to derive the percentage proportion of each categorization. **D** *Post-hoc* immunostaining of the pancreas slice shown in **(A)**, stained for glucagon (GCG, red) and insulin (INS, orange). **D-1** Ca^2+^ activity profiles of α-cells (red) and β-cells (blue) corresponding to the inset white square in **(D)** labeled “**1**.”. The same applies to **D-2** and **D-3**, which correspond to inset squares labeled “**2**.” and “**3**.”, respectively. Note the heterogeneous Ca²⁺ responses of α-cells across different regions of the islet, which occur in anti-phase to the Ca²⁺ activity of neighboring β-cells within that particular region. **E** The top left image shows topologically defined ROIs derived from a Ca²⁺ recording. The top right image shows the corresponding *post-hoc* immunostaining of the recorded islet, with GCaMP-expressing cells (green) and staining for glucagon (GCG, red), insulin (INS, orange), and somatostatin (SST, white). Inset squares **1**. and **2**. are enlarged in the top-middle panel, highlighting islet regions where α-, β-, and δ-cell ROIs are identified (left image) and confirmed by immunostaining (right image). The ROIs are numbered, and their corresponding Ca²⁺ activity profiles are shown in **E-1** and **E-2**. Note in **E-1:** an inactive α-cells is neighbored by an active δ-cells, whereas in E-2: an active α-cells is neighbored by a less active δ-cells. **F** This panel follows the same organizational layout as panel **(E)**, with regions of the islet (Insets 1 and 2) where α-, β-, and δ-cell ROIs are identified (left) according to the corresponding immunostaining (right), along with their respective Ca^2+^ activity profiles shown in **F-1** and **F-2**, respectively. **G** Schematic diagram of the islet microenvironment showing that α-cells in distinct regions (1–3) are surrounded by varying numbers of β- and δ-cells, potentially providing differential inputs to nearby α-cells (red arrows, inset). Created in BioRender. Rutter, G. (2026) https://BioRender.com/ofeyxcc.

In order to explore intercellular interactions and Ca^2+^ dynamics in islets, we employed live pancreas tissue slice preparation and high spatiotemporal resolution confocal imaging^36^. A glucose concentration ramp incrementing from 1.8 mM to 10.8 mM over 60 min (Fig. 2), mimicking changes in circulating glucose concentrations following a carbohydrate meal, was applied. In addition to recording the α-cells, which genetically express GCaMP6f, we further loaded a chemical Ca^2+^ indicator (Cabryte 590^AM^; emitting at 590 nm; Fig. 2, A-2) onto the slices to enable simultaneous tracking of Ca^2+^ activities in other cell types within the pancreas slices. Once recording was completed, the slices (140μm thick, ~10-14 islet cell layers) were subjected to *post-hoc* immunostaining with anti-glucagon, -insulin and -somatostatin antibodies. For the scope of this study, we focused only on islet α- (GCaMP6f- and glucagon-positive cells), β- and δ-cells (GCaMP6f-negative, but Calbryte 590^AM^ -, insulin- or somatostatin-positive cells).

We observed that, during an ascending glucose concentration ramp, Ca^2+^ dynamics in α-cells were markedly heterogeneous (Fig. 2B). Whilst a majority of α-cells (52.6 ± 4.2% and 54.0 ± 3.1% in female and male islets, respectively; Fig. 2B-Low glucose sensitive and Fig. 2C) displayed robust Ca^2+^ firing activities over the low glucose range between 1.8 mM to 5.4 mM, a minority of α-cells responded to higher glucose concentrations (15.2 ± 2.3% and 14.5 ± 2.6% in female and male, respectively; Fig. 2B-High glucose sensitive and Fig. 2C)), or remained constantly active throughout the glucose ramp (11.7 ± 1.9% and 9.2 ± 1.5% of all α-cells in female and male, respectively; Fig. 2B-Constantly active and Fig. 2C). Approximately a quarter of α-cells were relatively quiescent throughout the entire glucose concentration ramp (20.4 ± 2.6% and 22.1 ± 2.3% in female and male islets, respectively; Fig. 2B-inactive and Fig. 2C)). We note that these were live α-cells, as evidenced by occasional spontaneous Ca^2+^ oscillations and their intrinsic GCaMP6 fluorescence, which indicated their viability despite being otherwise functionally silent. We defined cells exhibiting less than five spikes throughout the course of glucose ramp as inactive cells.

### Glucose-regulated Ca^2+^ dynamics in α-cells are associated with Ca^2+^ dynamics in neighboring β- and δ-cells

**Fig. 3:**
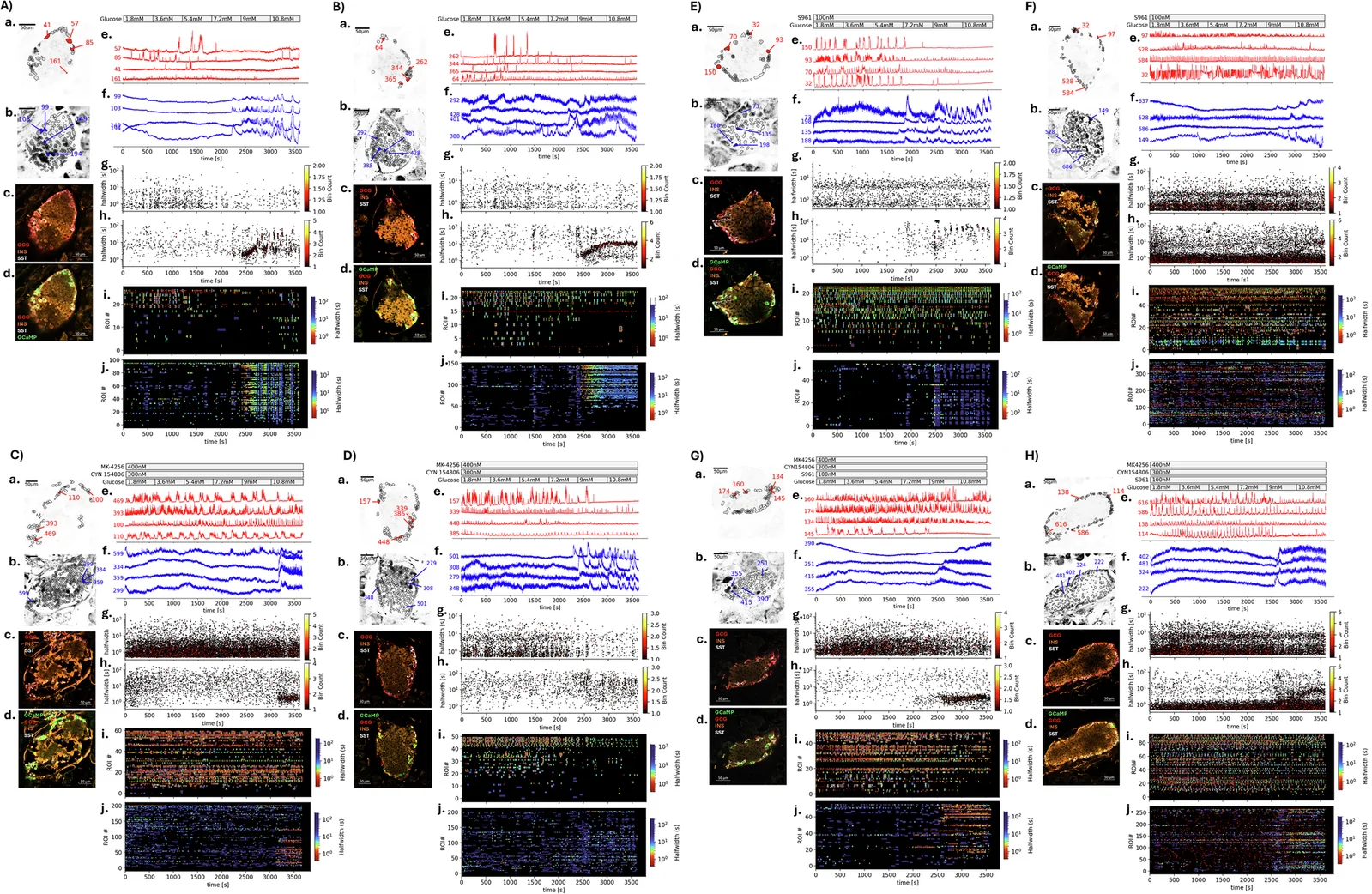
Modulation of α- and β-cell Ca^2+^ dynamics by insulin and somatostatin receptor antagonists in female and male mouse islets. **A**, **B** Control condition. A representative female (**A**) and male (**B**) mouse islet exposed to an incremental glucose concentration ramp (top right). ROIs corresponding to α- and β-cells (subpanels **a.** and **b.**, respectively) in each islet are shown in the left panels of **A** and **B**. Immunostaining for glucagon (GCG), insulin (INS), and somatostatin (SST) (**c.**), as well as GCaMP expression (**d.**), is displayed. Subpanels **e.**, **f.** show representative Ca^2+^ dynamics of α- and β-cells, topologically labeled in **subpanels a.** and **b., respectively**. Subpanels **g.** and **h.** show hexbin plots representing Ca^2+^ transient distributions in all α- (**g**.) and β-cell (**h**.) ROIs within an islet. Events are plotted as a function of peak time (x-axis; time [s]) and duration (y-axis; halfwidth [s]). Color intensity indicates event density according to the color bar on the right. Subpanels **i.** and **j.** show raster plots of Ca^2+^ transients in  all α- and β-cell ROIs, respectively, with each dot representing an event over time and color indicating event duration. An adjacent color bar (right) shows the mapping of halfwidth values on a logarithmic scale. **C–H** Pharmacological modulation. **Subpanels a.-j.** are as described for (**A**) and (**B**), show islet morphology, calcium dynamics, and event distributions under pharmacological modulation during the glucose ramp. **C**, **D** Representative female (**C**) and male (**D**) mouse islet exposed somatostatin receptor antagonists (MK-4256 and CYN154806), **E**, **F** Representative female (**E**) and male (**F**) mouse islets exposed to insulin receptor antagonist (S961). **G**, **H** Representative female (**G**) and male (**H**) mouse islets exposed to combined treatment (S961 + MK-4256 + CYN154806) applied throughout the glucose ramp to assess effects on Ca²⁺ activity in α- and β-cells.

To explore a possible role for neighboring cells in controlling α-cell dynamics, we investigated α-cells, along with adjacent β- and δ-cells, across different topological locations within individual islets. We found that the Ca^2+^ activities in α-cells at different islet regions (Fig. 2D) exhibited asynchronous patterns of Ca^2+^ activities between one another. Unlike β-cells^37^, α-cells exhibited weak connectivity in islets from both sexes (mean degree <2, clustering coefficient <0.2; Supplementary Fig. 1). Remarkably, Ca^2+^ dynamics in α-cells were always in antiphase with the nearby insulin-positive β-cells (Fig. 2D-1 to Fig. 2D-3), aligned with earlier findings^38^. Pearson correlation analysis performed on juxtaposed α- and β-cells also revealed a negative correlation in their Ca^2+^ traces, confirming an antiphase relationship between the two cell types (Supplementary Fig. 2A, B). This pattern was further supported by Pairwise Pearson Correlation analysis of concatenated fluorescence traces from all α-cell and β-cell ROIs within individual islets (Supplementary Fig. 2C).

We repeatedly observed antiphase activity between juxtaposed α- and β-cells in all islets examined. This suggested that either direct interactions exist between α- and β-cells, or that a third cell type (i.e., δ-cells) drives the “on-and-off” activity between α- and β-cells. Hence, we examined the Ca²⁺ dynamics in δ-cells that were neighboring α-/β-cell pairs (Fig. 2, E-F). An inverse relationship in Ca²⁺ activity was observed between δ-cells and adjacent α-cells during ramp stimulation, particularly within the glucose range between 1.8–7.2 mM when β-cells were largely inactive. We observed that active δ-cells were frequently adjacent to less active α-cells (Fig. 2E-1), and conversely, active α-cells were often juxtaposed to less active δ-cells (Fig. 2E-2). This spatial relationship was also evident in temporal activity patterns: as δ-cell Ca²⁺ activity declined (1.8–5.4 mM glucose; Fig. 2F-1), the nearby α-cell activity often increased. Conversely, periods of low δ-cell activity frequently coincided with heightened α-cell activity (1.8–5.4 mM glucose; Fig. 2F-2), with δ-cell activity activating (7.2–9.0 mM glucose; Fig. 2F-2) as α-cell activity declined. Quantification of active time in these cell pairs supported an inverse relationship in the robustness of Ca^2+^ oscillations between juxtaposed α- and δ-cells (Supplementary Fig. 3A). Of note, no obvious association was observed between δ- and the neighboring β-cell Ca²⁺ dynamics (Fig. 2E-1, E-2 and Fig. 2F-1, F-2) and the Ca^2+^activity in δ-cells was notably heterogeneous (Supplementary Fig. 3B), based on observations from 19 male islets (9 animals) and 33 female islets (5 animals) (Supplementary Table 1). It was therefore difficult to define a general pattern of δ-cell Ca^2+^ activity changes in response to the incremental glucose ramp, as the responses varied, showing increases, decreases, or no change over the course of ramp stimulation. Nevertheless, our observations indicated the possibility that α-cells are under the control of both β-cell (anti-phase Ca^2+^ activity relationship between α- and β-cells) and δ-cells (inverse relationship in the strength of Ca²⁺ activity between α- and δ-cells).

### Somatostatin, insulin, and glucose regulate α-cell Ca²⁺ activities

Within the islet microenvironment, α-cells in different regions are surrounded by differing numbers of β- and δ-cells (Fig. 2G, islet regions 1-3). Depending on the precise cell type composition, each of these different combinations could theoretically provide differential inputs into the nearby α-cells (Fig. 2G, inset circle red arrows), given that insulin and somatostatin, secreted by β- and δ-cells respectively, are well-known inhibitors of α-cells^39^. We therefore hypothesized that the interaction of α-cells with different neighboring combinations of β- and δ-cells underlies, or may contribute to, α-cell functional heterogeneity. Accordingly, administering either insulin receptor and/or somatostatin receptor antagonists during the glucose ramp on pancreas slices should shift most of α-cell Ca²⁺ activity toward hyperactivity, thereby altering heterogeneity.

### δ-cell control of α-cell function

We first tested paracrine regulation of α-cell Ca²⁺ activity by δ-cells by using either somatostatin or somatostatin receptor antagonists (SSTRA: 400 nM MK4256 and 300 nM CYN154806, which antagonize somatostatin receptors type 2 and 3 that are expressed mainly in mouse islet α-cells^40,41^). At a steady glucose level of 3.6 mM, where α-cell Ca²⁺ activity was high, SST inhibited α-cell Ca^2+^ activity in a dose-dependent manner, with stronger inhibition observed in α-cells from female islets (Supplementary Fig. 4A-B inset). Conversely, SSTRAs potentiated α-cell Ca^2+^ spike frequency in female islets (Supplementary Fig. 4D) but did so only in few male islets (Supplementary Fig. 4D). These results indicate that female α-cells are more sensitive to δ-cell-mediated somatostatin inhibition.

**Fig. 4:**
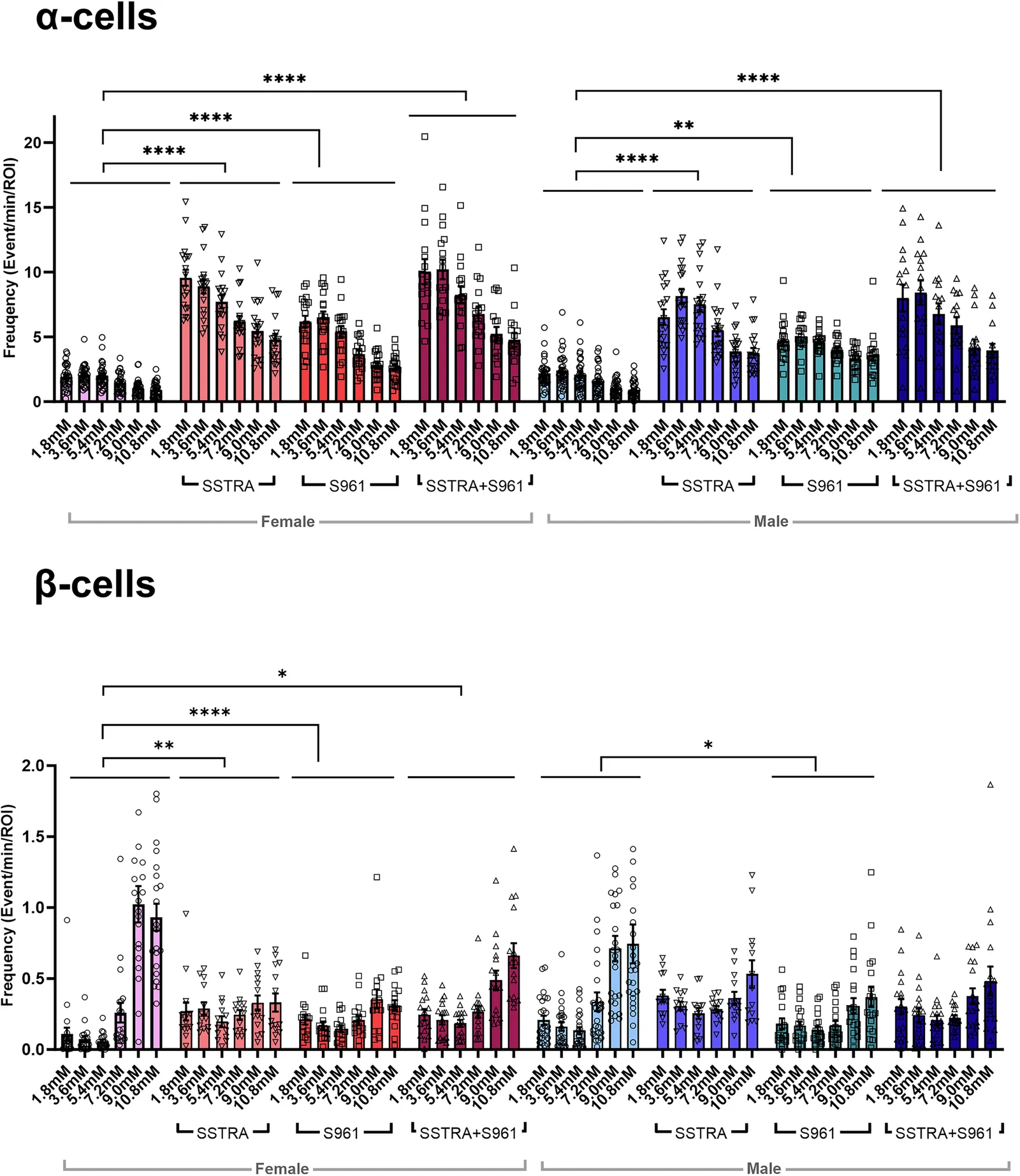
Summary of α- and β-Cell Ca^2+^ activity response across different glucose concentrations and receptor antagonists. Ca²⁺ oscillation frequencies in α-cells (top panel) and β-cells (bottom panel) were measured in response to an increasing glucose ramp alone or in combination with receptor antagonists. Bars represent mean ± standard error of the mean (SEM) Ca²⁺ oscillation frequencies in female (pink, orange, red, dark red) and male (light blue, purple, green, navy) islets under the following conditions: glucose ramp alone; glucose ramp + SSTR antagonists (SSTRA: 400 nM MK4256 + 300 nM CYN154806); glucose ramp + insulin receptor antagonist (S961: 100 nM); or glucose ramp + SSTRA + S961. Each data point indicates the mean Ca^2+^ activity frequency of α-cells (top) or β-cells (bottom) of an islet. **Top panel α-cells:** Glucose ramp only: *n* = 30 female and 31 male islets from 5 mice per sex (females, 85 ± 7.8 days; males, 76 ± 9.3 days); SSTRA: *n* = 18 female and 19 male islets from 3 mice per sex (females, 65.3 ± 5.5 days; males, 77.3 ± 3.8 days); S961: *n* = 19 female and 19 male islets from 3 mice per sex (females, 84 ± 2.0 days; males, 86.5 ± 10.1 days); SSTRA + S961: *n* = 16 female and 15 male islets from 3 mice per sex (females, 88 ± 7.0 days; males, 94.3 ± 6.4 days). The data were analyzed using a mixed-effects model, which accounts for repeated measure analyses and allows comparison of each treatment with control. In this model, islets are considered as the primary unit of analysis while accounting for glucose and treatment (where applicable) as fixed-effects and mouse as random intercept. Significance levels are indicated as: *p* < 0.05 (*), p < 0.01 (**), p < 0.001 (***), and p < 0.0001 (****). Effect size indicated as difference in means ± SEM. Females: Compared with control, SSTRA showed a difference of 5.55 ± 0.41 (95% CI: 4.75 to 6.35); S961 showed a difference of 2.95 ± 0.41 (95% CI: 2.16 to 3.75); and the combined SSTRA + S961 treatment showed a difference of 5.97 ± 0.41 (95% CI: 2.64 to 3.68). Males: Compared with control, SSTRA showed a difference of 4.18 ± 0. 50 (95% CI: 3.20 to 5.15); S961 showed a difference of 2.49 ± 0.50 (95% CI: 1.52 to 3.47); and the combined SSTRA + S961 treatment showed a difference of 4.64 ± 0.51 (95% CI: 3. 65 to 5.63). **Bottom panel β-cells:** Glucose ramp only: *n* = 21 female and 25 male islets from 5 female and 7 male mice (females, 85 ± 7.8 days; males, 78.7 ± 11.2 days); SSTRA: *n* = 14 female and 13 male islets from 3 mice per sex (females, 65.3 ± 5.5 days; males, 77.3 ± 3.8 days); S961: *n* = 16 female and 19 male islets from 3 mice per sex (females, 84 ± 2.0 days; males, 86.5 ± 10.1 days); SSTRA + S961: *n* = 16 female and 18 male islets from 3 mice per sex (females, 88 ± 7.0 days; males, 94.3 ± 6.4 days). The same statistical analysis as in the top panel was performed. Effect size indicated as difference in means ± SEM. Females: Compared with control, SSTRA showed a difference of -0.17 ± 0.06 (95% CI: -0.28 to -0.06); S961 showed a difference of -0.21 ± 0.06 (95% CI: -0.32 to -0.09); and the combined SSTRA + S961 treatment showed a difference of -0.11 ± 0.06 (95% CI: -0.23 to -0.01). Males: Compared with control, S961 showed a difference of -0.16 ± 0.05 (95% CI: -0.26 to -0.05).

**Fig. 5:**
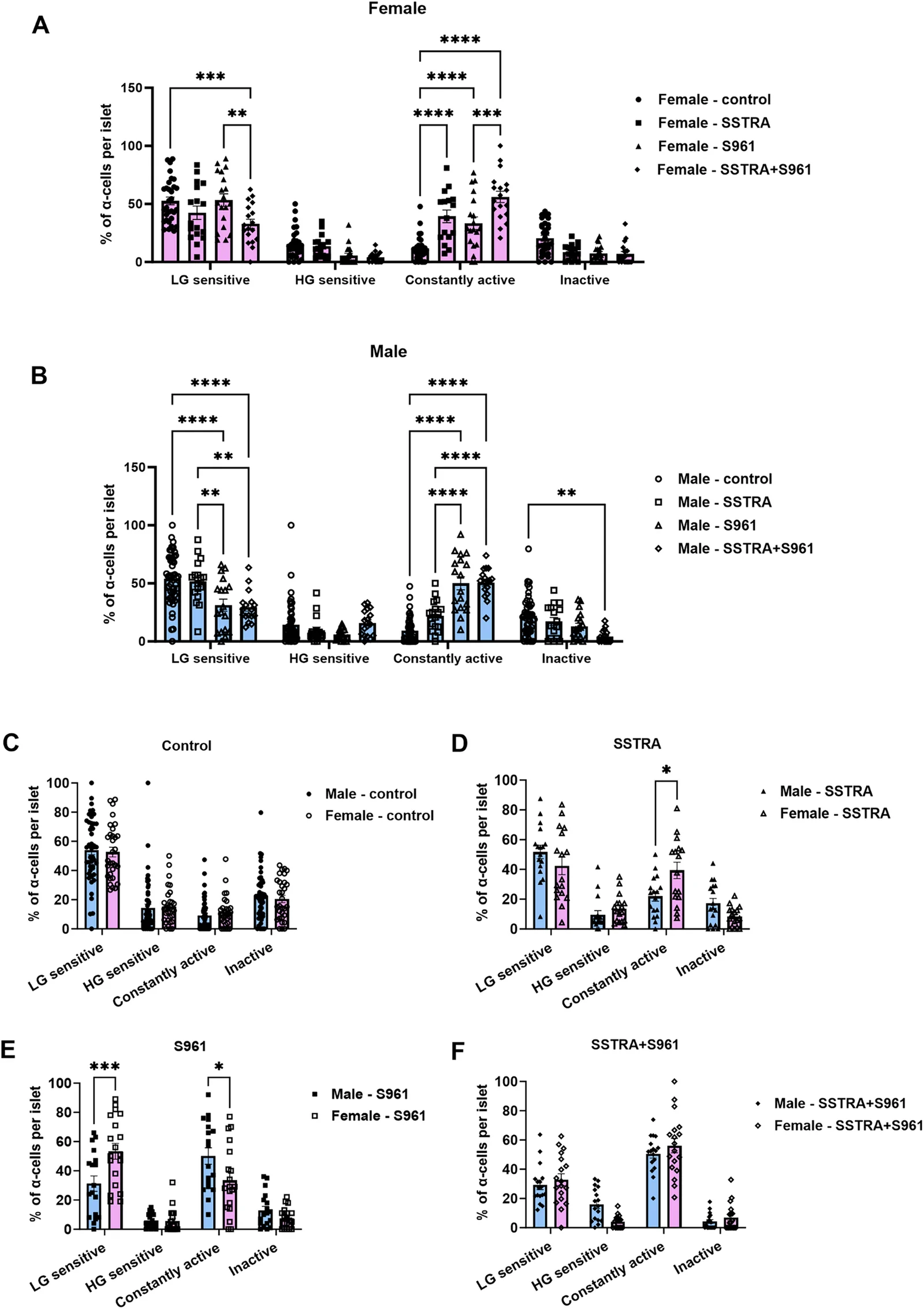
Classification of α-cell Ca²⁺ activity profiles in female (A) and male (B) islets across treatments. Islets from female (**A**) and male (**B**) pancreas slices treated with: glucose ramp alone (control), or in combination with SSTRA, S961, or both. α-cells in each islet were categorized based on their Ca²⁺ oscillation profiles into four groups as defined in Fig. 2C: low glucose (LG) sensitive, high glucose (HG) sensitive, constantly active, or inactive. **C**–**F:** Sex-based comparison of α-cell proportions in each oscillation category: glucose ramp alone (control; **C**), +SSTRA (**D**), +S961 (**E**), or SSTRA + S961 (**F**). Each symbol represents the percentage of α-cells relative to the total α-cell population per islet; each bars indicates mean ± SEM. These data were analyzed with a mixed-effects model, followed by Bonferroni’s post-hoc test. Islets are condisered as the primary unit of analysis while accounting for glucose and treatment (where applicable) as fixed-effects and mouse as random intercept. Significance levels are indicated as: *p* < 0.05 (*), p < 0.01 (**), p < 0.001 (***), and *p* < 0.0001 (****). Effect size indicated as difference in means ± SEM. **A** ***LG sensitive***: Control vs. SSTRA + S961 showed a difference of -19.74 ± 4.51 (95% CI: -35.67 to -3.82); S961 vs SSTRA + S961 showed a difference of -20.43 ± 5.01 (95% CI: -38.11 to -2.75); ***Constantly active***: Control vs. SSTRA showed a difference of 27.66 ± 4.69 (95% CI: 11.12 to 44.21); Control vs. S961 showed a difference of 21.77 ± 4.44 (95% CI: 6.11 to 37.43); Control vs. SSTRA+ S961showed a difference of 44.42 ± 4.51 (95% CI: 20.50 to 60.35); S961 vs SSTRA + S961 showed a difference of 22.65 ± 5.01 (95% CI: 4.97 to 40.33). **C** ***LG sensitive***: Control vs. S961 showed a difference of -22.81 ± 4.28 (95% CI: -37.92 to -7.70); Control vs SSTRA + S961 showed a difference of -24.70 ± 4.37 (95% CI: -40.14 to -9.27); SSTRA vs. S961 showed a difference of -19.79 ± 5.25 (95% CI: -38.34 to -1.25); SSTRA vs. SSTRA + S961 showed a difference of -21.68 ± 5.33 (95% CI: -40.49 to -2.88); ***Constantly active***: Control vs. S961 showed a difference of 43.19 ± 4.37 (95% CI: 27.76 to 58.63); Control vs. SSTRA + S961 showed a difference of 41.38 ± 4.37 (95% CI: 25.94 to 56.81); SSTRA vs. S961showed a difference of 30.48 ± 5.33 (95% CI: 11.68 to 49.29); SSTRA vs SSTRA + S961 showed a difference of 38.66 ± 5.33 (95% CI: 9.86 to 47.47). ***Inactive:*** Control vs. SSTRA + S961 showed a difference of -17.84 ± 4.37 (95% CI: -33.27 to -2.40). **D** ***Constantly active:*** Male vs. female: showed a difference of 17.43 ± 5.35 (95% CI: 0.71 to 34.16). **E** *LG sensitive*: Male vs. female showed a difference of 22.09 ± 5.49 (95% CI: 4.93 to 39.25). ***Constantly active:*** Male vs. female showed a difference of -18.94 ± 5.58 (95% CI: -36.36 to -1.52). Female islets, data were collected from *n* = 29 islets (5 mice, 85 ± 7.8 days old) under glucose ramp alone; *n* = 17 islets (3 mice, 77.3 ± 3.8 days) with SSTRA; *n* = 19 islets (3 mice, 86.5 ± 10.1 days) with S961; and *n* = 18 islets (3 mice, 94.3 ± 6.4 days) with SSTRA + S961. Male islets: glucose ramp alone included *n* = 47 islets (9 mice, 78.7 ± 11.2 days); SSTRA included *n* = 17 islets (3 mice, 65.3 ± 5.5 days); S961 included *n* = 19 islets (3 mice, 84 ± 2 days); and SSTRA + S961 included *n* = 6 islets (3 mice, 88 ± 7 days).

During glucose ramp stimulation, SSTRA triggered significant increases in α-cell Ca^2+^ activity in both female (Fig. 3C) and male islets (Fig. 3D; Fig. 4 and Supplementary Fig. 6 top panel: SSTRA treatment). Quantitative analysis of α-cell Ca²⁺ activity in individual islets showed that SSTRA treatment increased the proportion of constantly active α-cells (Fig. 5A, B: 37.4 ± 5.5% in females, 21.9 ± 3.3% in males, *p* < 0.0001 in females), more potently in the female islets (*p* < 0.05; Fig. 5D). The proportion of inactive (8.3 ± 1.6% in females, 17.2 ± 3.4% in males; Fig. 5A, B) and high glucose-sensitive α-cells under SSTRA treatment remained otherwise comparable to those observed with glucose ramp alone. (13.5 ± 2.3% in females, 9.6 ± 2.7% in males; Fig. 5A-B), although a trend toward reduced proportion of inactive α-cells was observed. These findings suggest that previously inactive α-cells may have transitioned into a constantly active state as a result of SST receptor antagonism. The notion that inhibition by somatostatin, presumably mediated by δ-cells under physiological conditions, contributes, at least to some extent, to the apparent heterogeneity of α-cell Ca²⁺ activity is supported.

Of note, during the SSTRA-supplemented glucose ramp, the decline in α-cell activities and the onset of β-cell Ca²⁺ activities, particularly in female islets (Fig. 3C; g–j), were both right-shifted towards higher glucose concentrations. That is, in the presence of SSTRA, α-cells remain activated above 5.4 mM glucose, where Ca²⁺ oscillations in most of α-cell typically declines, whereas β-cells showed reduced mean Ca²⁺ oscillation frequency at ≥ 9 mM glucose in islets of both sexes (Fig. 4 β-cells: SSTRA treatment), as a result of the shifted onset of their Ca^2+^ activation during the glucose ramp (Fig. 3C). This is also evident in the Ca²⁺ activity density plot (Supplementary Fig. 7C), where α-cell Ca²⁺ activity from all islets under SSTRA treatment is compiled.

**Fig. 6:**
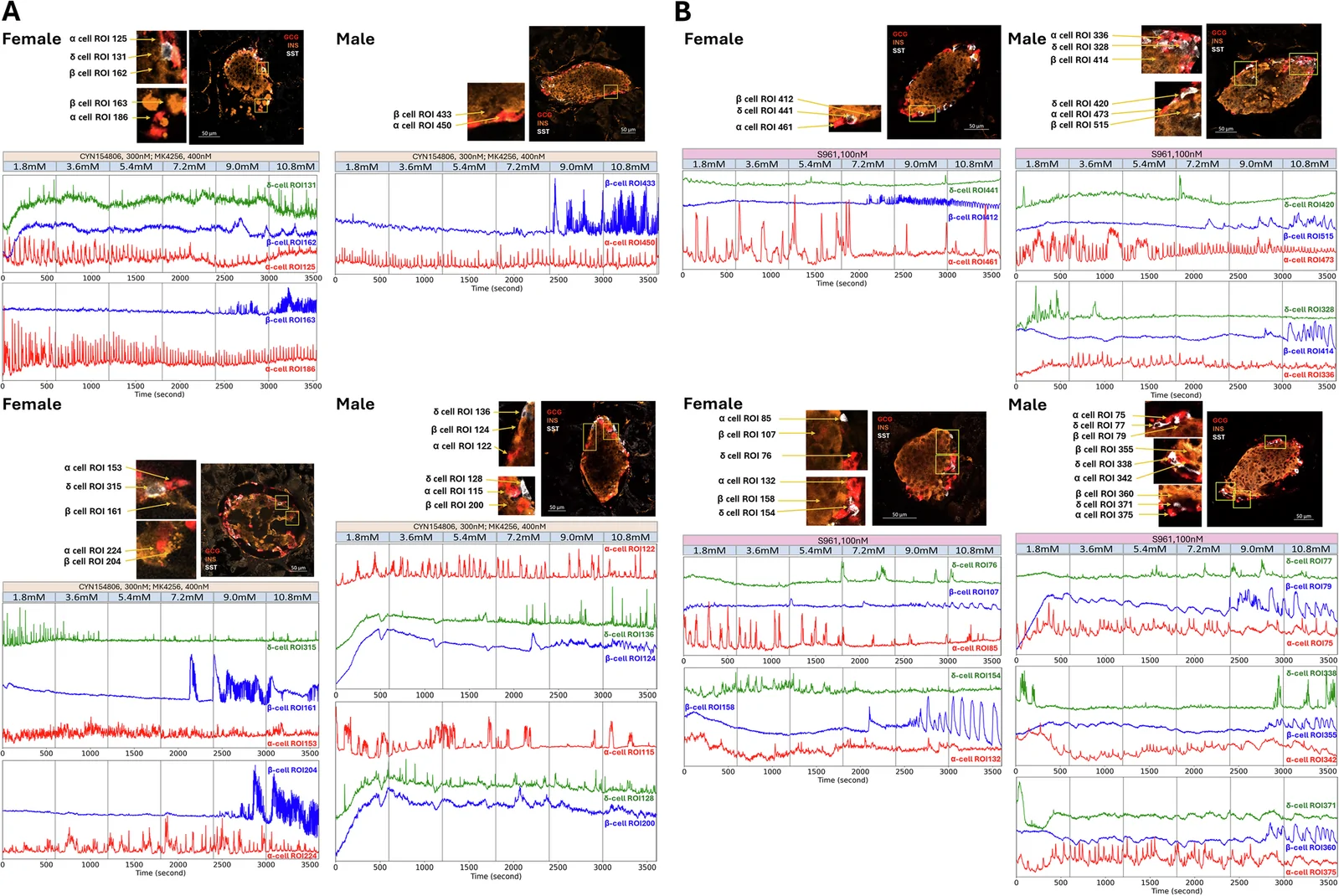
δ cell Ca²⁺ oscillation profiles in relation to neighboring β and α cells under SSTRA (6A) and S961 (6B) treatments. **6A** and **6B** shared the same organizational layout. **6A** shows islets treated with SSTRA, while **6B** shows islets treated with S961. The left panels of Figures **6A** and **6B** display two representitive female islets, while the right panels show two male islets, each stained for insulin (INS, orange), glucagon (GCG, red), and somatostatin (SST, white). Yellow squares within the islet images highlight regions containing identifiable α-, β-, and δ- cells, which are enlarged to the left of the islet images. Below these images, Ca^2+^ oscillation profiles of δ- (green), β- (blue), and α-cells (red) within the yellow squares are displayed and labeled with their respective ROI ID. Note in **6A**, the Ca^2+^ oscillations between δ-cells (green traces) and α-cells (red traces) no longer display an inverse relationship. In contrast, in **6B**, this inverse relationship between δ-cell and α-cell oscillations is still present.

Given that a chemical Ca²⁺ indicator was loaded into each slice, we explored whether δ-cell activity could be evaluated during SSTR agonist treatment. We found that δ-cell Ca²⁺ activity intensity relative to α-cell Ca²⁺ activity no longer exhibited the reciprocal pattern observed in the absence of the inhibitor (Fig. 6A), based on observations drawn from 18 male and 19 female islets (Supplementary Table 1).

### β-cell control of α-cell function

We next tested the paracrine effect of β- on α-cells using insulin or the insulin receptor antagonist, S961^42^. At 3.6 mM glucose, despite the addition of insulin, α-cell Ca^2+^ activity was only modestly reduced (Supplementary Fig. 5A, B). Consistently, the insulin receptor antagonist S961 did not significantly potentiate Ca^2+^ activity in α-cells in both female and male pancreas slices (Supplementary Fig. 5C, D). We therefore preincubated pancreas slices with S961 for 1 hour (hr) before subjecting them to a S961-supplemented glucose ramp. During the glucose ramps, S961 significantly enhanced α-cell Ca²⁺ activity over low glucose ranges (Fig. 3E, F, and Fig. 4 and Supplementary Fig. 6 top: S961 treatment). Particularly in male islets, S961 treatment notably altered α-cell glucose sensitivity (Fig. 3F; Supplementary Fig. 7D), shifting the suppression of α-cell Ca²⁺ oscillations and the activation of β-cell Ca²⁺ oscillations to occur at higher glucose concentrations ( > 7.2 mM). Consistent with this sex-dependent sensitivity of S961, quantitative analysis of α-cell Ca²⁺ activity revealed that S961 treatment increased the proportion of constantly active α-cells to 33.6 ± 5.4% in females vs. 48.1 ± 5.6% in males (*p* < 0.0001 comparing to control; Fig. 5A, B), with higher potency in male islets (*p* < 0.05; Fig. 5E), whereas the proportion of inactive (7.5 ± 1.6% in females, 13.2 ± 2.6% in males; Fig. 5A, B) and high glucose-sensitive α-cells (5.7 ± 1.9% in females, 5.5 ± 1.1% in males; Fig. 5A, B) were modestly reduced. These results reinforced the hypothesis that insulin inhibition, presumably mediated by β-cells in physiological contexts, also contributes, at least in part, to the heterogeneity of α-cell Ca²⁺ activity.

Similar to SSTRA treatment, mean β-cell Ca^2+^ oscillation frequencies under S961 treatment were reduced at ≥9 mM glucose (Fig. 4 bottom panel, S961 treatment). Moreover, β-cell activity under S961 treatment was no longer strictly in antiphase with that of α-cells when both cell types were active (Fig. 6B). Assessment of δ-cell Ca²⁺ activity under S961 treatment revealed that the intensities of α- and δ-cell Ca²⁺ activities continued to exhibit an inverse pattern (Fig. 6B), based on observations from 19 male and 19 female islets (Supplementary Table 1).

### Effects of glucose on α-cell Ca^2+^ activity

Lastly, we applied SSTR antagonists and S961 together (Fig. 3G, H). The combined antagonism of somatostatin and insulin receptors resulted in about half of α-cells shifting to a constantly active state (56.1 ± 4.9% in females, 50.6 ± 3.1% in males; *p* < 0.0001 for both sexes; Fig. 5A, B: SSTRA + S961), reducing the proportion of inactive α-cells (6.9 ± 2.2% in females, 4.2 ± 1.3% in males; *p* < 0.01 in males; Fig. 5A, B). Meanwhile, only approximately 30% of α-cells remained sensitive to low glucose (32.9 ± 4.0% in females, 29.3 ± 3.3% in males; both *p* < 0.0001 vs. control; Fig. 5A, B SSTRA + S961). Consequently, the overall α-cell Ca²⁺ activities, although significantly increased (Fig. 4 and Supplementary Fig. 6 top panel-SSTRA + S961), were still glucose concentration-dependent (Fig. 4 and Supplementary Fig. 6, top panel-SSTRA + S961; supplementary Fig. 7B).

## Discussion

In this study, we demonstrate that during incremental glucose ramps, α-cell Ca²⁺ oscillations exhibit marked heterogeneity. Importantly, α-cell Ca²⁺ oscillations occurred in anti-phase with neighboring β-cell Ca²⁺ oscillations, and the intensity of α-cell Ca²⁺ oscillations was inversely associated with that of the neighboring δ-cell’s Ca²⁺ oscillations. We further demonstrate that interfering with the paracrine communication, either between α- and β-cells and/or α- and δ-cells, by blocking either insulin receptor signaling alone, somatostatin receptor subtypes-2 and 3 alone, or both pathways simultaneously, leads to a large proportion of α-cells shifting toward hyperactivity. Interestingly, this occurred in a sex-dependent manner: female α-cells exhibited enhanced sensitivity to somatostatin-mediated signaling, whereas male α-cells were more responsive to insulin receptor signaling. Collectively, we suggest that α-cell excitability is finely tuned by inhibitory signals arising from the close juxtaposition of insulin- and somatostatin-secreting β- and δ-cells, in addition to α-cells’ *intrinsic* glucose sensitivity, likely involving intracellular metabolism of glucose and regulation of ATP-sensitive K^+^, Na^+^ and Ca^2+^ channels^43^. We note that the latter mechanism presumably persists even when SSTR and InsR signaling are blocked (Fig. 4 and Supplementary Fig. 6), consistent with the activating effects of deleting the key metabolic glucose sensor, glucokinase, selectively in α-cells^44^. These findings highlight the critical role of intra-islet coordination between different cell types in maintaining α-cell responsiveness. They also suggest that sex differences in paracrine signaling may contribute to different glucagon secretion profiles in metabolic disease.

We demonstrated that suppression of SSTR2 and SSTR3 signaling leads to α-cell hyperactivity, with their Ca^2+^ oscillations persisting at high glucose concentrations ( > 5.4 mM) particularly in females. To date, there is no evidence for sex-dependent differences in SSTR2 and SSTR3 expression in mouse α-cells. However, it has been suggested that the female sex hormone, estrogen, modulates SSTR expression in other tissues such as pituitary^45^, prostate^46^, and certain tumor cells^47^. It is therefore plausible that α-cells in female mice also express elevated levels of SSTR2 and SSTR3, leading to prolonged glucose-stimulated α-cell Ca^2+^ activity under SSTR2 and SSTRA3 antagonism. We acknowledge that although the estrous cycle in female mice was not controlled in our study, we expect the putative impact of estrogen to be relatively consistent across the estrous cycle in female mice. Previous studies have shown that the circulating estrogen levels remain higher in females than in males throughout the cycle ( ~ 17 pg/ml during estrus and diestrus, i.e., low-estrous phases, in females versus ~12 pg/ml in males)^48^.

We further reveal that insulin receptor antagonism by S961 leads to sustained α-cell Ca²⁺ activity in male mice under high glucose conditions, suggesting that male α-cells are more sensitive to insulin action. This heightened insulin sensitivity in male α-cells implies that, under conditions of insulin resistance in T2D or after β-cell loss in T1D, males may experience more pronounced hyperglucagonemia. This mechanism may help explain the higher prevalence of T1D or T2D in men^29,30^, as more severe hyperglucagonemia in males could drive excessive hepatic glucose production, contributing to both fasting and postprandial hyperglycemia.

We showed that, during an intraperitoneal glucose tolerance test (IPGTT), female GluCre:GCaMP6f mice exhibited better glucose clearance compared to males, despite having higher circulating glucagon levels. Females also had lower body weights compared to their male counterparts. These findings are consistent with previous reports demonstrating that chronic glucagon agonism^49^, or glucagon and GLP-1 co-agonism^50^, exerts antidiabetogenic effects in mice through whole-body metabolic mechanisms, including greater weight loss and improved glucose metabolism. Consistently, in a rat model, multiple low-dose glucagon injection into the mediobasal hypothalamus led to decreased hepatic glucose production and subsequent improved glucose tolerance^51^, mirroring our observations during IPGTTs in female GluCre:GCaMP6f mice. These effects are likely because glucagon is capable of crossing the blood-brain barrier^52^ and binding to glucagon receptors in the hypothalamus and brainstem^53,54^, which are regions involved in regulating glucose metabolism, energy expenditure, and appetite. Glucagon also stimulates hepatic synthesis and release of fibroblast growth factor 21 (FGF21), a circulating peptide hormone that contributes to energy homeostasis via centrally-mediated mechanisms^55^. Lastly, glucagon can elicit appetite-suppressing effect through the liver-vagus-hypothalamus axis^56^. Together, the central and peripheral actions of glucagon provide a mechanistic explanation for the improved glucose tolerance and reduced body weight observed in female GluCre:GCaMP6f mice in vivo.

We showed that islet insulin-positive area was smaller in female GluCre: GCaMP6f mouse islets (Fig. 1M), yet insulin sensitivities and circulating insulin levels were comparable between female and male GluCre: GCaMP6f mice. This suggests that female β-cells are more efficient in producing insulin than their male counterparts. This observation aligns with previous research^31^, which demonstrated that wild-type female mouse islet β-cells exhibit greater insulin secretion efficiency, attributed to more depolarized glucose-induced membrane potential and increased firing frequency. Correspondingly, clinical studies found that women have higher insulin secretion for a given glucose load^57^. Ex vivo glucose-stimulated insulin secretion assays performed on cadaver donor human islets obtained from age-, BMI-, and HbA1-matched donors also showed greater insulin secretion in female islets compared with male islets, despite comparable β-cell content in islets between sex^58^.

We uncovered that δ-cell Ca^2+^ dynamics in response to an increasing glucose ramp are heterogeneous, with no obvious pattern, and are not strictly associated with the Ca^2+^ oscillation pattern of the neighboring β-cells. Conversely, the rigor of δ-cell Ca^2+^ oscillations was always in an inverse relationship with that of α-cells. These findings deviate from previous reports suggesting δ-cells Ca²⁺ spikes increases with elevating glucose concentration^59^, as a result of direct electrical coupling and metabolic communication with between β- and δ-cells through gap junctions^8^. Since δ-cells have a neuron-like morphology characterized by a compact soma and dendrite-like extensions^59^, combined with their low abundance in islet and small cell size, we acknowledge that fewer δ-cells were successfully loaded, recorded, and confirmed as somatostatin-positive with *post-hoc* immunostaining in our study than β- or α-cells. Nevertheless, a newly published study using mouse models expressing GCaMP specifically in δ-cells in whole islets also reported heterogeneous δ-cell Ca²⁺ responses^60^, supporting our own observations. With an expanded sampling of δ-cells, the authors found that up to 23% exhibited either uncoordinated Ca²⁺ spikes or no activity under high-glucose conditions, possibly reflecting a subset of δ-cells lacking Cx36 gap junctions and therefore failing to exhibit coordinated Ca²⁺ dynamics with neighboring β-cells.

In fresh, live pancreas slices, only ~15% of α-cells were active at elevated glucose ( > 7.2–10.8 mM) during a gradual glucose ramp (1.8–10.8 mM), and these cells showed no islet-wide Ca^2+^ oscillation synchronization (Supplementary Fig. 1). This contrasts with a previous study using a stepped increase in glucose (3–10 mM) to evoke Ca²⁺ oscillations in isolated, cultured mouse islets, in which large proportion ( ~ 50%) of genetically-labeled α-cells became active at 10 mM glucose and their Ca²⁺ oscillations were synchronized and globally phase-locked with β-cells, which were only partially labeled across the islet^38^. These differences likely reflect distinct tissue preparations, β-cell labeling strategies, and glucose stimulation protocols. In particular, islet mantle-localized α-cells are exposed to enzymatic and culture-related stress during islet isolation. Moreover, contributions from unlabeled β-cells and δ-cell-mediated paracrine signaling, which were not explicitly examined by Ren et al.^38^, may further contribute. These factors may collectively account for the divergent α-cell functional properties observed in the two studies.

A recent study found that removing SST signaling in isolated mouse islets lowered the glucose threshold for β-cell Ca²⁺ oscillations^61^. In contrast, our work in fresh pancreas slices showed that SST signaling blockade shifted both the decline of α-cell activity and the onset of coordinated β-cell Ca²⁺ oscillations to higher glucose concentrations, particularly in female islets, requiring higher glucose levels. We consistently observed this shift of functional responses among β-cells when decline of α-cell activity was shifted to higher glucose concentrations, either by SST receptor antagonism in female islets or insulin receptor antagonism in male islets. These findings suggested that α- and β-cells coordinated closely to shape overall islet function, which is consistent with data presented herein and that of Ren et al.^38^, showing antiphase (phase-locked) Ca²⁺ oscillations between α- and β-cells. We note that although the onset of β-cell Ca^2+^ oscillations is shifted to higher glucose concentrations, insulin output may still be substantially potentiated once coordinated Ca²⁺ oscillation among β-cells are established. Our glucose ramp protocol regretfully did not allow assessment of β-cell functional responses above 10.8 mM glucose, as the protocol was originally designed to prioritize detailed assessment of α-cell Ca²⁺ activity. Nevertheless, our findings are consistent with hormone secretion assay reported by Huang et al.^61^ They demonstrated that glucose-stimulated insulin secretion in δ-cell-ablated SST-Cre×DTR islets was unchanged at 3, 7, 11 mM glucose and enhanced only at 16.8 mM. This pattern mirrors the rightward shift in β-cell functional responsiveness observed in our pancreas slice experiments, rather than indicating earlier activation at lower glucose concentrations.

In the study by Huang et al.^61^, immunostained islet images appear to indicate that GCaMP expression was present in only a subset of β-cells in the mouse model used, and α-cell Ca²⁺ dynamics were unfortunately not examined in parallel. These limitations hindered mechanistic insights as to why activation thresholds for coordinated β-cell activities are reset to lower glucose concentrations in response to SST signaling disruption, particularly given the tightly coordinated regulation between α- and β-cell function. We also note that differences in experimental models, i.e., acute pharmacological SST blockade in intact pancreas slices versus chronic SST depletion or genetic δ-cell ablation in animal model and cultured islets, could underlie the divergent outcomes between the two studies. We acknowledge that future studies using GLP-1R or GCGR inhibitors could help clarify whether α-cell hyperactivity is a causal factor in shifting the threshold of β-cell activation, providing further mechanistic insight into the intercellular coordination within the islet.

### Limitations of the study

While our study offered novel insights into sex-specific islet cell Ca²⁺ dynamics in response to glucose, key questions remain. First, ~10% of insulin-positive cells expressed GCaMP6f in our mouse model. While these cells were identified as β-cells according to their Ca²⁺ dynamics, which resembled those of non-GCaMP6f-expressing β-cells within the same islets, we cannot entirely exclude the possibility that they represent a distinct α-cell subpopulation. Further work specifically characterizing this subpopulation may provide fundamental insights into α-/β-cell plasticity and potential transdifferentiation. Second, our ex vivo slice preparation is limited in capturing potential in vivo inputs such as innervation, blood flow, and circulating metabolites^62^. Thus, we cannot exclude these factors as potential contributors to α-cell regulation. We also note that the current analysis of α-cell regulation by neighboring β- and δ-cells is confined to interactions within the imaging plane. Three-dimensional time-lapse imaging encompassing the entire islet would provide a more comprehensive understanding of intra-islet cellular regulation. However, this approach is currently limited by available imaging technologies, as increased volumetric acquisition inherently reduces temporal resolution, thereby compromising the accurate capture of the high-frequency dynamics characteristic of α- and β-/δ-cell activity. Addressing these technical limitations in the future will be an important objective for improving our understanding of islet cell communication. Third, we recognize that cytosolic Ca²⁺ changes may not fully represent hormone secretion in either β-^63^ or α-cells^64^. In particular, some Ca²⁺ signaling persists in α-cells even under glucose-inhibited conditions^1^ and glucagon secretion is regulated by both channel-specific Ca²⁺ influx^65^ and downstream cAMP-dependent amplification^66^. Nevertheless, we emphasize that it remains one of the most informative approaches for studying islet physiology at *single-cell resolution*, particularly for interrogating islet intercellular communication^36^, which is the central focus of this study. Technologies that allow simultaneous single-cell measurements of hormone secretion in intact islets are still limited and technically challenging; most secretion assays (e.g., ELISA or perfusion-based methods) report hormone release at the whole-islet level and therefore lack the spatial resolution needed to link secretion to individual cells or to resolve cell-cell interactions, underscoring the need for future methodological development in this area. We note that recent studies using zinc release as a surrogate for that of insulin^64^, as well as our earlier studies^67^ using a similar approach to achieve single cell resolution, though elegant, do not necessarily report *insulin* release per se (the fusion pore diameter required for the latter being substantially larger than that required for zinc release). Fourth, since insulin and SST receptor antagonists were applied globally in intact pancreas slices, where native cell-cell interactions were preserved, blocking SST or insulin receptors may concurrently have altered input strength from neighboring β- or δ-cells, which expressed somatostatin and insulin receptors as well^40,68^. This complexity made it difficult to isolate direct effects of insulin or somatostatin on α-cells in pancreas slices. Nonetheless, our approach offered valuable mechanistic insights into islet α-, β-, and δ-cells interactions under both unperturbed and perturbed paracrine signaling in the most physiologically relevant ex vivo environments. Fifth, we cannot exclude inherent transcriptomic^69^ and proteomic^70^ heterogeneity among individual α-cells, including those showing β-cell-like Ca²⁺ responses to elevated glucose. Such genetic and molecular diversity may underlie the different Ca²⁺ signaling phenotypes observed among α-cells in our recordings. While single-cell RNA-Seq^71^ supported functional heterogeneity in α-cells in pancreas slices, future studies combining functional imaging with single-cell transcriptomics^72^, immunocytochemistry^73^ or in situ hybridization^74^ may offer an additional means to assess this possibility. Finally, it will be important in the future to extend our findings to human pancreas, where the higher proportion and intermingling between α-/β-cells and other islet cells^22^ may further emphasize paracrine regulation.

In summary, our study provided a simultaneous, cell type-specific characterization of Ca²⁺ dynamics in pancreatic α-, β-, and δ-cells in response to dynamic glucose changes. These findings revealed heterogeneity in α-cell Ca^2+^ dynamics, which is shaped both by glucose but also by insulin and somatostatin secreted from neighboring β-, and δ-cells. We further identified sex-dependent differences in α-cell responses and heterogeneity, laying the groundwork for future investigations. Ultimately, these insights may contribute to the development of more targeted and effective strategies for diabetes treatment.

## Methods

### Generation of GluCre:GCaMP6f mouse model

All animal procedures complied with the University of Montreal Animal Care and Use Committee (CIPA2022-10,040 CM21022GRs to GAR) and all relevant ethical regulations for animal use. GluCre:GCaMP6f mouse model (Fig. 1A) was generated by crossing GluCre mice (obtained from Jackson Laboratory Strain #:030663) to mice that express GCaMP6f downstream of a LoxP-flanked STOP cassette (The Jackson Laboratory, stock no. 028865). Age-matched male and female mice, aged 60-100 days, were used in the study. All mice had free access to water, regular chow and were housed under a 12-hr light/dark cycle.

### In vivo measurements

GluCre:GCaMP6f mice were either fasted for 16 hrs prior to fasting blood glucose measurement or assessed between 9:00-11:00 AM without fasting for resting blood glucose measurement. Blood glucose was measured from pre-nicked mouse tails using a glucometer (Ascensia, Mississauga, ON, Canada). Insulin (ITT) and glucose tolerance (IPGTT) tests were conducted as previously described^27,75^. Briefly, mice were fasted either overnight for 16 hrs (for IPGTT) or 4 hrs (for ITT), with ad libitum access to water. Glucose (2 g/kg body weight) or Insulin (0.75 U/kg body weight, Novolin®ge Toronto, Novo Nordisk, ON, Canada) was diluted in sterile PBS and administered via intraperitoneal injection. Blood glucose levels were recorded at baseline (0 min) and 15-, 30-, 60-, and 90-minutes post-injection. For serum insulin and glucagon levels, blood was collected from the pre-nicked tail using capillary tubes (KIMBLE DWK Life Science, USA), supplemented with HALT protease inhibitor (Thermo Fisher Scientific, Canada), and spun at 10,000 g for 10 min. Serum insulin and glucagon levels were measured using ELISA Kits (ALPCO Diagnostics, New Hampshire, USA; Mercodia, Uppsala, Sweden) per manufacturer’s instructions.

### Pancreas slice preparation, Ca^2+^ indicator loading, and immunostaining

GluCre:GCaMP6f mice were euthanized by cervical dislocation prior to pancreas excision. Pancreas slices (140 μm thick) were prepared following protocols described previously^36^ and maintained in HEPES-ECS (in mM: 125 NaCl, 10 NaHCO3, 10 HEPES, 6 glucose, 6 lactic acid, 3 myo-inositol, 2.5 KCl, 2 Na-pyruvate, 2 CaCl2, 1.25 NaH2PO4, 1 MgCl2, 0.25 ascorbic acid; titrated to pH 7.4 using 1 M NaOH). For Ca²⁺ imaging, slices were loaded for 2 hrs in HEPES-ECS containing the fluorescent indicator Calbryte 590^AM^ (15 μM; AAT Bioquest, CA, USA), 0.03% Pluronic F-127 (w/v), and 0.12% dimethyl sulfoxide (v/v). Imaging was performed on a confocal microscope (Zeiss LSM-900, Airyscan) equipped with temperature control (37°C) using a 20×/0.8 Plan-Achromat objective. Images were acquired at 6.25 Hz in a single plane (256×256 pixels) without averaging, using the 488 and 561 nm laser lines for GCaMP6f and Calbryte 590^AM^ excitation, respectively. During recording, pancreas slices were continuously perifused with HEPES-extracellular solution (ECS)^36^ at 1 ml/min supplemented with incremental (1.8, 3.6, 5.4, 7.2, 9.0, 10.8 mM) glucose concentration and pharmacological compounds as indicated. S961 (Novo Nordisk (Denmark) compound sharing program), insulin (Novolin®ge Toronto, Novo Nordisk, ON, Canada), somatostatin-14 (Sigma-Aldrich, ON, Canada), MK4256 (MedChemExpress LLC, NJ, USA) and CYN154806 (Tocris Bioscience) were freshly prepared before each experiment. In experiments assessing insulin signaling in α-cells, pancreas slices were preincubated with S961 in HEPES-ECS for 1 hr (except Supplementary Fig. 5C, D) before the start of the glucose ramp, with S961 present continuously in the perfusate during the ramp. Movies were subjected to semi-automatized analytical pipeline that were developed previously^36,76^.

### Immunofluorescence staining

Once image acquisition was completed, pancreas slices were immediately fixed in PBS containing 4% paraformaldehyde for 20 min, then permeabilized for 15 min at room temperature with 0.3% Triton X-100 in PBS. Islet containing pancreas slices were incubated overnight at 4 °C in PBS containing 0.3% Triton X-100, 5% bovine serum albumin, a primary monoclonal mouse anti-glucagon antibody (1:1000; Sigma-Aldrich, Canada), a polyclonal guinea pig anti-insulin antibody (1:10; DAKO, Santa Clara, CA, USA), and a monoclonal rat anti-somatostatin antibody (1:500; Invitrogen). The following day, pancreas slices were PBS-washed and incubated for 4 h at room temperature with secondary anti-rat, anti-guinea pig, and anti-mouse antibodies conjugated with Alexa Fluor 405, 561, and 647 (1:250 dilution). Slices were then PBS-washed and imaged immediately. GCaMP, Insulin (INS), Glucagon (GCG), Somatostatin (SST) and total islet area were determined using ZEISS ZEN 3.7 software on the acquired images.

### Classification of α-cells based on Ca^2+^ activity patterns

α-Cell identity was assigned only to ROIs showing both strong, clearly localized glucagon immunofluorescence and GCaMP6f expression. ROI boundaries were defined and Ca^2+^ oscillation traces from these ROIs were extracted using our previously reported, validated automated segmentation pipeline^76^. α-Cells were categorized semi-automatically based on their Ca^2+^ activity patterns during glucose concentration ramps. We applied a custom Python script that segmented the recording into 6 time-intervals matching the six glucose concentrations on the ramp and quantifies Ca^2+^ events per segment per α-cell ROIs. For each cell, we calculated the spikes (events) per interval, total event count, and the relative distribution across intervals. Cells were categorized as: “inactive”, if all six time intervals exhibited fewer than five Ca^2+^ events; “constantly active”, if events were distributed relatively uniformly across all intervals, with a variation ≤15% between segments; “low-glucose sensitive”, if the combined activity during the first three intervals (corresponding to the low-glucose phase) exceeded that of the last three; and “high-glucose sensitive”, if the opposite pattern was observed, with greater activity in the final three intervals (high-glucose phase).

### Spatial and functional correlation analysis of α- and β-cells

Ca^2+^ oscillation traces from α- and β-cell ROIs within each islet were extracted and concatenated for analysis^76^. Pairwise Pearson correlation coefficients were computed between all α- and β-cell traces to quantify their functional relationships. For spatial analysis, the x-y coordinates of each ROI were used to calculate the distance between cell pairs. To investigate local paracrine interactions, we considered α- and β-cell pairs that are within 25 µm apart, given that β-cells average 10–13 µm in diameter and mouse α-cells are smaller than β-cells^5^. Negative correlations (r<0) between nearby α- and β-cell pairs were visualized by plotting cell locations as scatter color-coded coordinates with lines connecting the negatively correlated pairs.

### Pairwise Pearson correlation analysis

Ca^2+^ oscillation traces were aligned in time and detrended to remove baseline fluctuations. The Pearson correlation coefficient measures the linear relationship between two time series according to the formula:

$$r=\frac{\mathop{\sum }_{i=1}^{n}\left({X}_{i}-\bar{X}\right)\left({Y}_{i}-\bar{Y}\right)}{\sqrt{\mathop{\sum }_{i=1}^{n}{\left({X}_{i}-\bar{X}\right)}^{2}}\sqrt{\mathop{\sum }_{i=1}^{n}{\left({Y}_{i}-\bar{Y}\right)}^{2}}}$$

where ${X}_{i}$ and ${Y}_{i}$ represent fluorescence values at time point i from two cells, and $\bar{X}$ and $\bar{Y}$ are their respective mean fluorescence values. A coefficient of r=1 indicates perfect positive correlation, r=−1 indicates perfect negative correlation, and r=0 indicates no linear correlation.

### Network analysis and cell active time

We constructed functional α-cell networks from Ca²⁺ dynamics using NetworkX in two steps as described previously^76^. First, correlations c(i,j) for all pairs (i,j) of α-cells are obtained from recorded time series of Ca²⁺oscillation. Second, these correlations were compared against a threshold (c₀ = 0.7): if c(i,j)>c₀, a line was placed between cells i and j. Networks were generated across six temporal segments corresponding to the glucose ramp. Connected nodes were visualized in red and isolated nodes in navy. For each network, we quantified mean degree (average number of links per node) and clustering coefficient (tendency of nodes to form local clusters). Mean degree values of 0-2 indicated sparse connectivity with mostly isolated cells; 3-7 indicated weakly connected networks with limited hubs; 8–20 reflected functional networks with coordinated communication; and >20 suggested highly synchronous networks. Clustering coefficients of 0-0.1 indicated disconnected clusters, 0.1–0.3 partial local coordination, 0.3–0.6 functional subnetworks, and 0.6–1.0 strong local hubs.

### Quantification of cell active fraction

For each ROI, Ca²⁺ events were grouped into consecutive 60-second intervals, and the total time during which Ca²⁺ signals were elevated was calculated. The active fraction was defined as the total active time divided by 60 s for each interval. Median active fractions across each glucose concentration range for each cell type were then computed and plotted to compare the temporal activity dynamics of α- and δ-cell ROIs.

### Statistics

Statistical analysis was performed (GraphPad Prism 10) with appropriate tests detailed in each figure legend; *p-value* < *0.05* was considered statistically significant.

### Reporting summary

Further information on research design is available in the Nature Portfolio Reporting Summary linked to this article.

## Supplementary information

Transparent Peer Review file

Supplementary Information

Description of Additional Supplementary files

Supplementary data 1

Reporting summary

## Data Availability

The numerical source data for all graphs in the manuscript can be found in Supplementary data 1 file.

## Code availability

Custom Python scripts developed to support the findings of this study are available on GitHub and Figshare^77^: https://doi.org/10.6084/m9.figshare.31988721. For video processing: https://github.com/szarma/Physio_Ca/; for Ca^2+^ trace analysis: https://github.com/MSlakRupnik/Alpha_cell_analysis_for_publication. The software versions used are: Python 3.10.10 | packaged by conda-forge | (main, Mar 24 2023, 20:08:06) [GCC 11.3.0]; networkx 3.0; numpy 1.23.5; scipy 1.10.1; pandas 1.3.5. The commit used for this manuscript is: **d81961bf20e7005197dc9ffac706280492ad4b80**.
