# Supplementary material for: Pancreatic α-cells are functionally heterogeneous and sex-dependently regulated by neighboring endocrine cells
Source: Commun Biol. 2026 Jun 23;9:1324. doi: 10.1038/s42003-026-10163-6 (PMC13655181; doi:10.1038/s42003-026-10163-6)

nature portfolio

Peer Review File

Pancreatic α-cells are functionally heterogeneous and sex-dependently regulated by neighboring endocrine cells

Corresponding Author: Dr Ya-Chi Huang

This manuscript has been previously submitted at another journal. This document only contains information relating to versions considered at Communications Biology.

This file contains all reviewer reports in order by version, followed by all author rebuttals in order by version.

Version 0:

Reviewer comments:

Reviewer #1

(Remarks to the Author)
The authors image mouse pancreatic slices expressing GCaMP6f selectively in alpha-cells (plus a red Ca2+ dye for neighboring beta/delta-cells) during a glucose ramp. They report: (1) marked heterogeneity of alpha-cell Ca2+ oscillation patterns; (2) robust anti-phase coupling between alpha- and beta-cells and an inverse relationship in activity "strength" between alpha- and adjacent delta-cells; and (3) paracrine control that is sex-dependent—female alpha-cells show greater sensitivity to SSTR2/3 blockade, whereas male alpha-cells are more sensitive to insulin-receptor antagonism (S961). Combined SSTR2/3 and InsR blockade shifts ~50–56% of alpha-cells to a constantly active state but preserves overall glucose dependence.

Significance and novelty
The demonstrated simultaneous, cell-type–resolved Ca2+ imaging across alpha/beta/delta cells in intact pancreas slices represents a strong platform to dissect intra-islet coordination; pairing this with pharmacologic dissection of SSTR2/3 and InsR signaling and explicit sex comparisons is novel and important. The paper moves beyond "alpha-cells are heterogeneous" by proposing a concrete paracrine scheme with sex biases. The methods for functional coupling (Pearson correlations/networks) and the open code enhance impact and repeated application of the methods.

The authors' claims are largely supported by convincingly showing alpha cell heterogeneity and alpha to beta antiphase across many islets. Also, the classification bins are defined a priori and applied systematically.

The delta-cell inverse association with alpha-cells is supported by representative traces plus spatial pairing supports the claim and the authors appropriately note delta-cell heterogeneity. The authors establish the sex-dependent paracrine control with dose–response to SST/SSTRA and S961 and the effects are quantified including the female bias to SST inhibition and male bias to InsR blockade are shown. The shift to "constantly active" alpha-cells with preserved glucose dependence during the combined SSTR and InsR antagonism is clearly demonstrated.

Major strengths
1.High-quality large-scale pancreatic slice imaging with cell-type identification, plus careful post-hoc immunostaining.
2.Clear quantification and availability of custom analysis scripts (e.g Ca2+ trace analysis).
3.The authors provide adequate per-group sampling at the islet level, with clear reporting of n of islets per mice in key figures.

Points needing clarification or additional analysis:

1.Statistical design and unit-of-analysis
The manuscript mixes "per-islet" quantification with multiple islets from the same mouse (pseudoreplication risk). Figure legends mention two-way ANOVA/Tukey and a separate note mentions mixed effects, but the random effect (mouse) isn't consistently specified. The authors will need to (1) state the primary unit of analysis per endpoint; (2) use or confirm mixed-effect models with mouse as a random intercept for all per-islet outcomes; and (3) report effect sizes with CIs alongside p-

values.

2.Robustness of alpha-cell “state” classification
Please show that conclusions are insensitive to the spike-count thresholds and the segmentation windowing (e.g., sensitivity analysis varying “inactive” <5 spikes and the c0=0.7 correlation threshold for networks). A small supplement figure would suffice.

Supplementary data

3.Linking Ca2+ to secretion:
Ca2+ is an accepted proxy, but not a perfect surrogate for hormone output. Consider adding (or discussing) parallel glucagon secretion measurements from matched conditions or cite constraints explicitly as limitations.

4.Delta-cell sampling
Authors note delta-cell heterogeneity and limited sampling. It would help to quantify delta-cell counts per islet or condition and show summary statistics for delta-cell activity bins to support generalized statements.

5.Sex biology context
The sex-biased effects are compelling. If feasible, please indicate whether estrous cycle was controlled or logged in females, or state explicitly that it was not and discuss potential impact.

6.GCaMP6f “leakage” into beta-cells
About 10% of GCaMP regions were insulin positive. The authors restrict alpha-cell analyses to dual-positive glucagon+GCaMP ROIs, which is appropriate. Still, a brief control showing that key results are unchanged when excluding border or ambiguous ROIs would strengthen confidence.

Reproducibility

Core analysis code is publicly available on GitHub. Please add commit hashes and a minimal “runbook” - inputs to outputs and example datasets or HDF5 traces sufficient to re-run a figure-level analysis. Also, please state the software versions used for network metrics.

Minor comments
•Report exact drug pre-incubation times and wash-in kinetics for S961 in the main Methods (which is currently scattered).
•Standardize units across figures (e.g., glucose mM; event/min/ROI).
•Where possible, include mouse-level n alongside islet-level n in all panels.

Additional limitations that should be noted
Because SSTR2/3 and InsR antagonists were applied globally in intact slices, observed alpha-cell changes may partially reflect indirect effects via beta- and delta-cells that also express these receptors. Authors need to discuss this explicitly. Also, while mouse slices are physiologically relevant, confirmation in human pancreas with its higher alpha and beta cell intermix will be important for human translation. Given the authors’ acknowledgment of alpha-cell molecular heterogeneity, a brief note on how this could underlie different Ca^{2+} phenotypes would round up the limitation section.

Reviewer #2

(Remarks to the Author)
Huang et al studied islet cell Ca2+ activities (proxy for hormone secretion) in pancreatic slices focusing on glucagon-releasing alpha cells and their dependence on neighbouring insulin and somatostatin secreting beta and delta cells, respectively, when the slices were exposed to gradually increasing glucose concentration. They found that the alpha cell responses during a glucose ramp were markedly heterogenous, but in general in opposite phase to or inversely correlated to the beta and delta cell responses. Exposure to inhibitors of insulin receptors or somatostatin receptors 2+3 strongly activated most alpha cells and shifted their inhibition by glucose to higher concentrations in parallel with a similar shift in the onset of beta cell Ca2+ activity. Female alpha cells were more sensitive to somatostatin receptor inhibition and male alpha cells to insulin receptor inhibition. However, when insulin and somatostatin receptor inhibition were combined the alpha cells showed increased Ca2+ activity but the sensitivity to glucose remained. The authors speculate about the role of intra-islet paracrine coordination in the tuning of alpha cell glucose responses and the potential importance of the observed sex differences in the development of diabetes.
The manuscript is not easily digested. There is plenty of data and the text does not follow the Figures numbers. Instead, one has to jump back and forth between Figures and conditions. I think the manuscript could be considerably improved in this respect. The study confirms previous observations of alpha cell heterogeneity and that they are glucose-regulated both via paracrine interactions and intrinsic effects. The major novel aspect is the somewhat unexpected differences between sexes. I find the study interesting but raise a number of issues for the author’s consideration.

Major issues.
1. In the introduction and later in the manuscript “synchronized beta cell Ca2+ oscillations” are mentioned. In other instances, “synchronized beta cell Ca2+ activity” and “synchronized Ca2+ activation” are used. Extremely well synchronized beta cell oscillations have been shown in many studies but synchronization in the present traces is far from impressive although the beta cells show higher connectivity than the alpha cells.

2. The authors acknowledge the weakness in using Ca2+ as a proxy for secretion. This weakness is probably accentuated by focusing on Ca2+ oscillation frequency. The Ca2+ patterns are thus very different in different cells with short-lived spiking in some, more prolonged oscillations and even sustained elevation in others. A better proxy for secretion is therefore probably area under the curve, when comparing different conditions in individual cells.
3. As stated above some data are confirmatory and it would be appropriate to cite earlier findings of alpha cells heterogeneity, e.g. alpha cells with beta cell-like glucose sensitivity (PMID: 27539300).
4. The observed Ca2+ patten might also be influenced by the indicator used. It would therefore be valuable to compare the GCaMP6f responses of the alpha cells with their Calbryte 590 responses.
5. The confocal imaging was done in a single plane of 140 µm thick pancreatic slices 10-14 cell layers. How deep into this slice was this plane? I suppose that different cell types were only identified within this plane? Please clarify and discuss potential influence from not identified cells in other planes.
6. Line 171: “Counterintuitively”, well, not necessarily since glucagon amplifies insulin secretion.
7. When examining the Ca2+ dynamics between delta and alpha/beta cell pairs an inverse relationship between delta and adjacent alpha cells was observed. However, in Fig 2E-1 the alpha and beta cells remain essentially inactive independent of delta cell activity. Moreover, the activity of the delta cell declines with increasing glucose concentrations (also in Fig 2-F) although somatostatin secretion is characteristically stimulated by increasing glucose. These delta cell Ca2+ responses seem more like what might be expected from alpha cells. Moreover, I cannot follow the authors argumentation about Fig 2F-2 (lines 225-226) “with δ-cell activity resuming (7.2–9.0 mM glucose; Figure 2F-2) as α-cell activity declined”. First, the alpha cell activity declined already in 5.4 mM glucose and the resuming δ-cell activity is unconvincing.
8. In lines 228-230 the authors point out the lack of correlation between delta and beta cell activity. This is not unexpected below the glucose threshold for beta cell activation but it would be appropriate to discuss the lack of correlation at higher glucose concentrations since pulsatile insulin and somatostatin secretion have been found to be essentially in phase (e.g. PMID: 22227186). This may be added to the discussion about beta-delta cell gap junction coupling (around line 385).
9. Although it is stated on lines 325-326 that the somatostatin receptor subtypes-2 and 3 were blocked alone, or both, I think they were only blocked together. This is a pity since blocking the receptors separately would have allowed conclusions about separate effects on alpha and beta cells. It is stated in lines 269-270 “SSTRA-supplemented glucose ramp, β-cell Ca2+ activity frequency was increased at basal glucose concentrations (1.8-5.4mM glucose in both sex; Figure 4 β-cells: SSTRA treatment)”. One wonders how this activity (events/min Fig 4) is defined when comparing Figs 3-1A and B. This is related to issue 2 above. The only thing that is very apparent is the onset of beta cell activity at the higher glucose concentration. Returning to the role of the different SSTRs: are the effects of SSTRAs on beta cells due to inhibition of their SSTRs or indirect and related to increased glucagon secretion after blocking the alpha cell SSTRs? This is one issue that might have been resolved by separate receptor SSTR blocking and further clarified with antagonists of glucagon action (glucagonR and GLP-1R blocking).
10. A particular peculiarity is the right-shifted glucose cell threshold after SSTRA. As discussed in lines 394… removal of SST signalling has previously been found to lower the cell glucose threshold. A lowering of the threshold makes more sense to me considering the amplifying action of glucagon/cAMP on insulin secretion. The authors also make a point of the observation that a decline in alpha cell Ca2+ activity is associated with the appearance of “synchronized” beta cell activity (lines 271-273). This does not make sense considering the just mentioned amplifying effect of glucagon on insulin secretion. Again, experiments with separate SSTR subtype antagonists and glucagonR and GLP-1R blocking may shed light upon the discrepancy with previous data.
11. Referring also to issue 2 above about Ca2+ as proxy for secretion I wonder what the significance is of the “significantly blunted Ca2+ oscillation frequency at >9 mM glucose” in beta cells and why this is “a result of the delayed onset of their synchronized Ca2+ activation during the glucose ramp” (lines 275-277). Incidentally, “synchronized” seems correctly used in the latter sentence since is refers to the onset of Ca2+ activation.
12. Referring to issue 9 above there is a similar peculiarity in the effect of the insR inhibitor in associating the decrease of alpha cell activity at higher glucose concentration with the appearance of “synchronized beta cell Ca2+ oscillations”.
13. Again referring to issues 2 (about Ca2+ as proxy for secretion) and 10, I wonder what the significance is of the blunted Ca2+ oscillation frequencies at >9 mM glucose in response to insR inhibitor (line 304).
14. The inverse relationships between alpha and delta cell activities under insR inhibition is not particularly convincing (lines 307-308, Fig 6B).
15. The statements in lines 356-358 that glucagon agonism or glucagon-GLP-1 co-agonism exerts antidiabetic effects and improves glucose metabolism seem inconsistent with the present findings that SSTR and insR antagonism right shift the glucose threshold for glucose activation of the cells despite maintaining alpha cell activity at higher glucose concentrations.
Minor issues.
16. The term “immunoassay” is repeatedly used when the authors apparently mean “immunostaining”, which is also used. I think that the latter more precisely describes what was done.
17. Line 179: Should probably be “express” rather than “expressing”.
18. Lines 270 and 276: Should probably be “sexes” rather than “sex”
19. Figure 4 beta cells: It is difficult to follow the lines indicating significances.

Reviewer #3

(Remarks to the Author)
The manuscript by Ya-Chi Huang et al. utilizes pancreatic slice calcium imaging to examine islet cell responses from male and female mice to secretagogues and in relation to neighboring cells. The authors find that alpha-cell Ca^{2+} oscillations were heterogeneous and inversely correlated with delta-cell calcium responses. The manuscript also finds that blocking insulin receptors (with S961) and/or somatostatin receptors increased the excitability of alpha-cells slightly differently in male compared to female alpha-cells. The manuscript further identified differences in alpha-cell responses as well as heterogeneity of these responses in male versus female islets. As pancreatic slice technology maintains the islet

microenvironment, the data are physiologically relevant. The data is of high quality and improves the understanding of islet cell calcium handling, paracrine signaling, and sex differences of islet cell calcium responses. There are some minor areas that require attention that are detailed below:

The authors find that, “Remarkably, Ca2+ dynamics in α-cells were always in antiphase with the nearby insulin-positive β-cells (Figure 2D-1 to Figure 2D-3), aligned with earlier findings39.” However, the manuscript also finds that glucose inhibits calcium in many of the alpha cells. Thus, some discussion about the differences observed from the findings in reference 39 is warranted. It would also be nice to see a zoomed view of some of the antiphase firing from one islet especially “in α-cells at different islet regions (Figure 2D) exhibited asynchronous patterns of Ca2+ activities.” It is interesting that “α-cells exhibited weak connectivity in islets from both sexes” yet “were always in antiphase with the nearby insulin-positive β-cells.” At the top of the plateau potential all beta-cells are electrically connected and show rapid electrical signaling across the entire islet, which the authors observe in calcium traces (such as Fig. 3-1A traces). Thus, have the authors looked at the top of the plateau potential to see if the asynchronous patterns of single islet alpha-cell Ca2+ activities show greater synchronization?

The authors show an increase in islet cell activity following treatment with a variety of receptor inhibitors. In Figure 3-1 C-e the red representative alpha cell traces show a clear reduction of activity in response to elevated glucose levels. However, the hexbin plots of Ca2+ transient distributions (3-1 C-g) and the raster plots of Ca2+ transients (3-1 C-i) show that the glucose-dependence of alpha cell calcium responses is lost. An area under the curve measurement may allow for a clearer analysis of the changes of calcium. Or the representative traces are not truly representative. The same holds true for some of the beta-cell graphs. For example, Figure 3-1 B-f and 3-2 B-f shows blue traces that show a clear increase in calcium in response to elevations in glucose, although with some baseline drift. It seems like the changes in calcium in low glucose are mostly drift or movement and not typical of a normal beta cell calcium response. What is considered an amplitude of response that can be measured? Based on the representative beta cell traces there is some noise in the recordings, and it will be important to include some supplementary figures or methods indicating what was considered noise, drift, or movement versus a true beta calcium response. Some of the beta and delta cell responses clearly show movement such as in Fig. 6A delta cell ROI136 and 128 at the lowest glucose and similarly beta cell ROI124 and 200. What is considered movement or imaging artifact in the analysis could be more clearly defined.

Minor:
The following sentence might have a mistake,” These findings revealed heterogeneity in α-cell Ca2+ dynamics, which is shaped both by glucose but also by insulin and glucagon secreted from neighboring β-, and δ-cells.” It seems like the second glucagon in this sentence should be somatostatin.

Version 1:

Reviewer comments:

Reviewer #1

(Remarks to the Author)
I am happy with authors' revision of the manuscript

Reviewer #2

(Remarks to the Author)
The manuscript by Huang et al was improved by the revision. However, it is still not easily digested despite some rearrangements to improve alignment between text and illustrations. All reviewers have drawn attention to the use of Ca2+ as a proxy for secretion or whether it is average Ca2+ or frequency of Ca2+ changes that is important. I would like to return to this issue, which I think may shed light on some very unexpected results in the manuscript.

Ca2+ is a trigger of secretion in all islet cells but the relationship between Ca2+ concentration and the rate of secretion is far from clear. Ca2+ is basal in β cells exposed to low glucose and secretion is not triggered. There is consequently little effect of amplifiers of insulin release in this situation. When the threshold for glucose-stimulated Ca2+ influx is exceeded, secretion is triggered, and now amplifiers have potent effects. The situation in the glucose-regulated α cells is different. The relative glucose effect on glucagon secretion is less prominent than on insulin release. In particular, glucose doesn’t turn off glucagon secretion completely (PMID: 21824262, PMID: 17136393) which is consistent with many observations that some Ca2+ signalling remains in glucose-inhibited α cells. Moreover, in the K-ATP-channel-centred model for glucagon secretion, Ca2+ influx through certain channels is more important for secretion than the total influx (PMID: 22177710). Since Ca2+ signalling remains in glucose-inhibited α cells, secretion-amplifying factors become important, and it has even been suggested that Ca2+ is a permissive factor that allows cAMP to mediate glucose regulation of glucagon secretion (PMID: 30953108).

Against this background I would like to return to my previous concern about the right-shifted β cell glucose threshold after SSTRA. When describing this phenomenon (lines 278-280) it is emphasized that the decline in α-cell Ca^{2+} activities and onset of β-cell Ca^{2+} activities were both right-shifted towards higher glucose concentrations. Later in discussion (lines 426-428) SST signalling blockade is instead stated to prolong α-cell hyperactivity and delay activation of coordinated β-cell Ca^{2+} oscillations. If the authors consider involvement of a temporal aspect, that need to be clarified. Otherwise, I suggest that

“delay” is used with caution. Referring to the published data about antiphase Ca^{2+} oscillations between α- and β-cells (ref 40) the authors argue that “It is therefore intuitive that prolonged α-cell activity delays the onset of coordinated β-cell oscillation during the incremental glucose ramp observed” (433-435). Well, it might be superficially intuitive when considering the antiphase Ca2+ oscillations alone and disregarding that this antiphase relationship was observed in the absence of SSTRA. However, if the α cell Ca2+ oscillations reflect glucagon secretion, as assumed, it is instead counterintuitive that inhibited release of this potent amplifier of insulin secretion coincides with activation the of insulin release. It seems much more reasonable that prevention of SST signalling should lower the glucose threshold for insulin secretion as reported in ref 63.

In their reply to my previous concern about the unexpected SSTRA effect on the β cell glucose threshold the authors emphasize that the α cells, rather than the β cells, are the focus of the current study. The authors nevertheless emphasize the α β cell connection with parallel but opposite right-shifts Ca2+ signalling but do not suggest any mechanism. Indeed, also I find it natural to focus on α β cell interactions since the effect is induced by SSTRA and persists when the insulin receptors are blocked. In any case this peculiar effect, which questions the use of α cell Ca2+ as proxy for glucagon secretion needs to be properly discussed.

Reviewer #3

(Remarks to the Author)
The manuscript by Ya-Chi Huang et al. utilizes pancreatic slice calcium imaging to examine islet cell responses from male and female mice to secretagogues and in relation to neighboring cells. The data does improve the understanding of islet cell calcium handling. While the authors addressed all concerns some of these comments were a bit concerning.

For example two reviewers asked for area under the curve (AUC) analysis and the authors indicate that, "Ca^{2+} oscillation frequency is, in our view, a more reliable proxy for hormone secretion than the total signal magnitude." The authors also indicate that, "Furthermore, oscillations in intracellular Ca^{2+} concentration (leading to repetitive Ca2+ increases, and in contrast to sustained Ca2+ elevations) in islet endocrine cells are the key determinant of the probability (and thus the rate) of granule exocytosis." In response to this:
Frequency is important, but amplitude does have significant impacts on secretion (e.g., KCl stimulation causes greater secretion). I am a bit surprised by the authors argument against AUC. Especially when considering that line-scanning confocal imaging would never allow the primary calcium signal (the millisecond based action potential-induced calcium spike) to be detected. Most electrophysiology is recorded at 20 Khz or faster, which is 3200 times the speed of 6.25 hz. At 6.25 hz an image is taken every 120 milliseconds. As an action potential waveform from a beta-cell is typically ~10 milliseconds in duration, with 6.25 hz imaging speed only one image during an action potential would be possible and only if it was taken at the right time (the image could hit the peak of an action potential or a point between action potentials or a point on the upstroke or repolarization of an action potential). More importantly, alpha-cells fire sodium channel dependent action potentials that are even faster <5 milliseconds in duration. To see a complete action potential calcium response, you would need to image the calcium during the action potential upstroke as well as repolarizing phase, which the line-scanning confocal approach cannot. Therefore, this statement about calcium frequency is a bit misleading, because you are missing most of the action potentials that actually stimulate secretion. Importantly, the frequency plots of this manuscript do not indicate the primary calcium kinetics of an islet cell that occur during an action potential. Thus, the frequency argument against using AUC is flawed with this experimental approach.

Version 2:

Reviewer comments:

Reviewer #2

(Remarks to the Author)
I am essentially satisfied with the second revision but somewhat confused by the statement in lines 435-437, which may require some clarification. The “We note-sentence” seems to state that insulin secretion is not necessarily reduced before Ca2+ elevation is induced at a higher glucose threshold. From their argumentation I think the authors agree that Ca2+ elevation is required for stimulated insulin secretion.

Reviewer #3

(Remarks to the Author)
The manuscript authors have carefully and thoroughly responded to the reviewer comments, which have addressed all of my concerns. I believe that this is an outstanding manuscript suitable for publication in Communications Biology. The data is of exceptional quality and greatly improves the understanding of islet cell calcium handling, paracrine signaling, and sex differences of islet cell calcium responses.

Open Access This Peer Review File is licensed under a Creative Commons Attribution 4.0 International License, which permits use, sharing, adaptation, distribution and reproduction in any medium or format, as long as you give appropriate credit to the original author(s) and the source, provide a link to the Creative Commons license, and indicate if changes were made.
In cases where reviewers are anonymous, credit should be given to 'Anonymous Referee' and the source.
The images or other third party material in this Peer Review File are included in the article's Creative Commons license, unless indicated otherwise in a credit line to the material. If material is not included in the article's Creative Commons license and your intended use is not permitted by statutory regulation or exceeds the permitted use, you will need to obtain permission directly from the copyright holder.
To view a copy of this license, visit https://creativecommons.org/licenses/by/4.0/

Reviewer #1 (Remarks to the Author):

The authors image mouse pancreatic slices expressing GCaMP6f selectively in alpha-cells (plus a red Ca2+ dye for neighboring beta/delta-cells) during a glucose ramp. They report: (1) marked heterogeneity of alpha-cell Ca2+ oscillation patterns; (2) robust anti-phase coupling between alpha- and beta-cells and an inverse relationship in activity "strength" between alpha- and adjacent delta-cells; and (3) paracrine control that is sex-dependent—female alpha-cells show greater sensitivity to SSTR2/3 blockade, whereas male alpha-cells are more sensitive to insulin-receptor antagonism (S961). Combined SSTR2/3 and InsR blockade shifts ~50–56% of alpha-cells to a constantly active state but preserves overall glucose dependence.

Significance and novelty
The demonstrated simultaneous, cell-type–resolved Ca2+ imaging across alpha/beta/delta cells in intact pancreas slices represents a strong platform to dissect intra-islet coordination; pairing this with pharmacologic dissection of SSTR2/3 and InsR signaling and explicit sex comparisons is novel and important. The paper moves beyond "alpha-cells are heterogeneous" by proposing a concrete paracrine scheme with sex biases. The methods for functional coupling (Pearson correlations/networks) and the open code enhance impact and repeated application of the methods.

The authors' claims are largely supported by convincingly showing alpha cell heterogeneity and alpha to beta antiphase across many islets. Also, the classification bins are defined a priori and applied systematically.

The delta-cell inverse association with alpha-cells is supported by representative traces plus spatial pairing supports the claim and the authors appropriately note delta-cell heterogeneity. The authors establish the sex-dependent paracrine control with dose–response to SST/SSTRA and S961 and the effects are quantified including the female bias to SST inhibition and male bias to InsR blockade are shown. The shift to "constantly active" alpha-cells with preserved glucose dependence during the combined SSTR and InsR antagonism is clearly demonstrated.

Major strengths
1.High-quality large-scale pancreatic slice imaging with cell-type identification, plus careful post-hoc immunostaining.
2.Clear quantification and availability of custom analysis scripts (e.g Ca2+ trace analysis).
3.The authors provide adequate per-group sampling at the islet level, with clear reporting of n of islets per mice in key figures.

Points needing clarification or additional analysis:

1.Statistical design and unit-of-analysis
The manuscript mixes “per-islet” quantification with multiple islets from the same mouse (pseudoreplication risk). Figure legends mention two-way ANOVA/Tukey and a separate note mentions mixed effects, but the random effect (mouse) isn’t consistently specified. The authors will need to (1) state the primary unit of analysis per endpoint; (2) use or confirm mixed-effect models with mouse as a random intercept for all per-islet outcomes; and (3) report effect sizes with CIs alongside p-values.

> We are grateful to the reviewer for their constructive feedback. We have re-run all the analyses as suggested. Specifically, Figures 4, 5 and Supplementary Figure 4 and 5 using a mixed-effect model with islets as the primary unit of analysis while accounting for glucose and treatment (where applicable) as fixed-effects and mouse as random intercept. Effect size has been reported as the difference in means ± SE with 95% confidence intervals alongside p-values. We note that the results of these new analyses do not affect main conclusions of the study.

2.Robustness of alpha-cell “state” classification
Please show that conclusions are insensitive to the spike-count thresholds and the segmentation windowing (e.g., sensitivity analysis varying “inactive” <5 spikes and the c0=0.7 correlation threshold for networks). A small supplement figure would suffice.

> We thank the reviewer for the suggestion and have tested these parameters as below:

1: Sensitivity analysis varying the “inactive <5 spikes” threshold
We compared our baseline definition (inactive <5 spikes) with a stricter threshold (inactive <2 spikes) by reanalysing the set of alpha cells that underwent control treatment (glucose ramp only). As shown in the figure below (to avoid unnecessary complexity for the reader, we did not include this as a supplementary figure in the manuscript), the summary statistics (**A** and **B**) were not meaningfully different between the thresholds, demonstrating that our conclusions are robust to reasonable variations in the inactive-spike definition. Thresholds above 5 spikes would incorrectly label moderately-firing cells as inactive, hence these were not performed.

A. B.

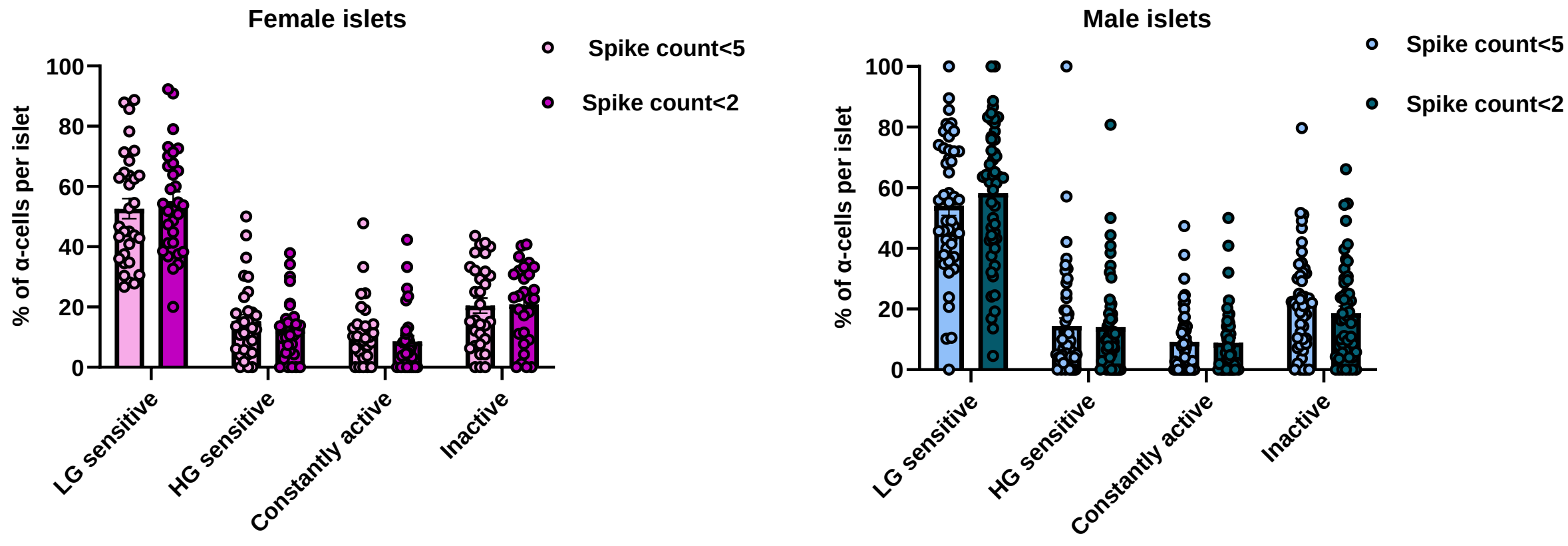

2: c0=0.7 correlation threshold for networks

>In the original manuscript, we adopted a correlation threshold of c0=0.7 to assess putative α-cell functional networks in pancreatic slices. This choice is based on our previously-published work using mathematical modeling of β-cell functional networks in pancreatic tissue slices (PMID: 25598507). In that study, defining functional connections (edges) using a correlation cut-off of ≥0.7 yielded biologically plausible network properties, including small-world organization and realistic degree distributions (mean degree ~5–15 per node), consistent with prior experimental observations in pancreatic islets.

In response to the reviewer's suggestion, we additionally tested c0 = 0.6 and c0 = 0.8, below and above c0 = 0.7. As shown below (**C-E**), neither of the alternative threshold yielded mean degrees or clustering coefficients consistent with meaningful network structure, supporting our conclusion that α-cells do not form a functional correlation-based network. Again, to avoid unnecessary complexity for the reader, we did not include this as a supplementary figure in the revised manuscript.

C. C0:0.6

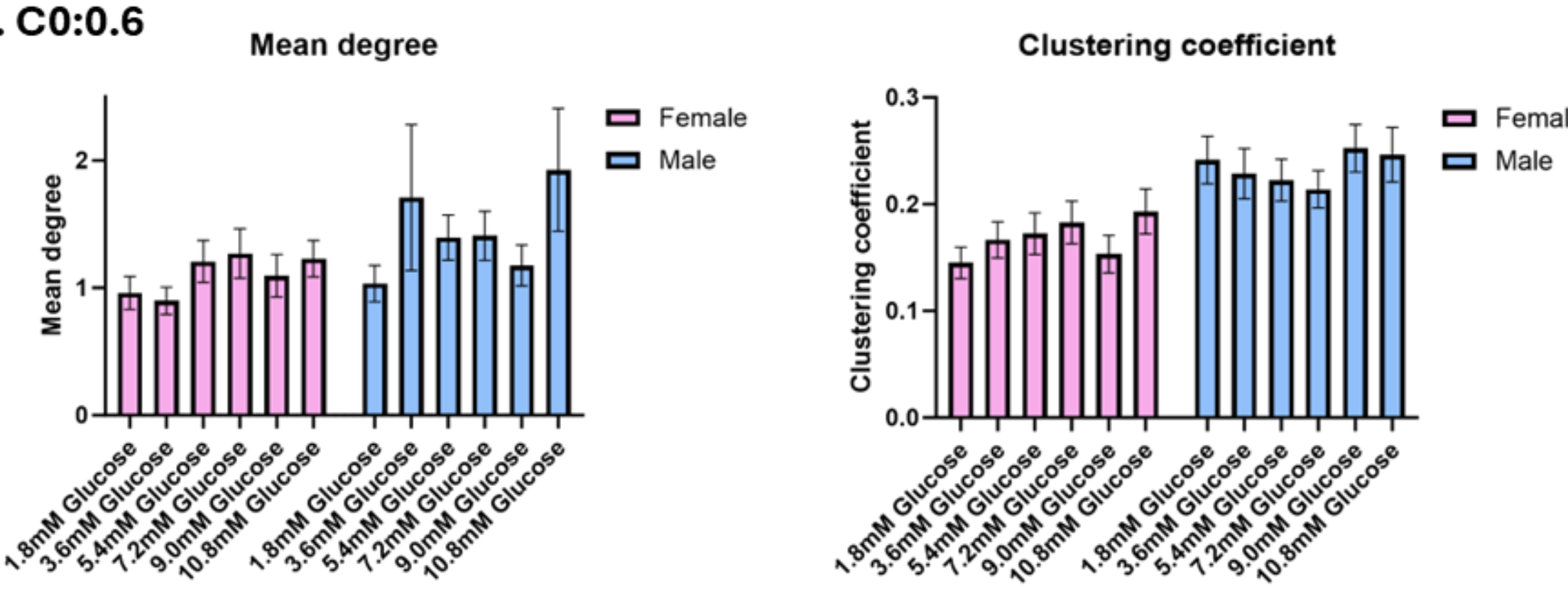

D. C0:0.7

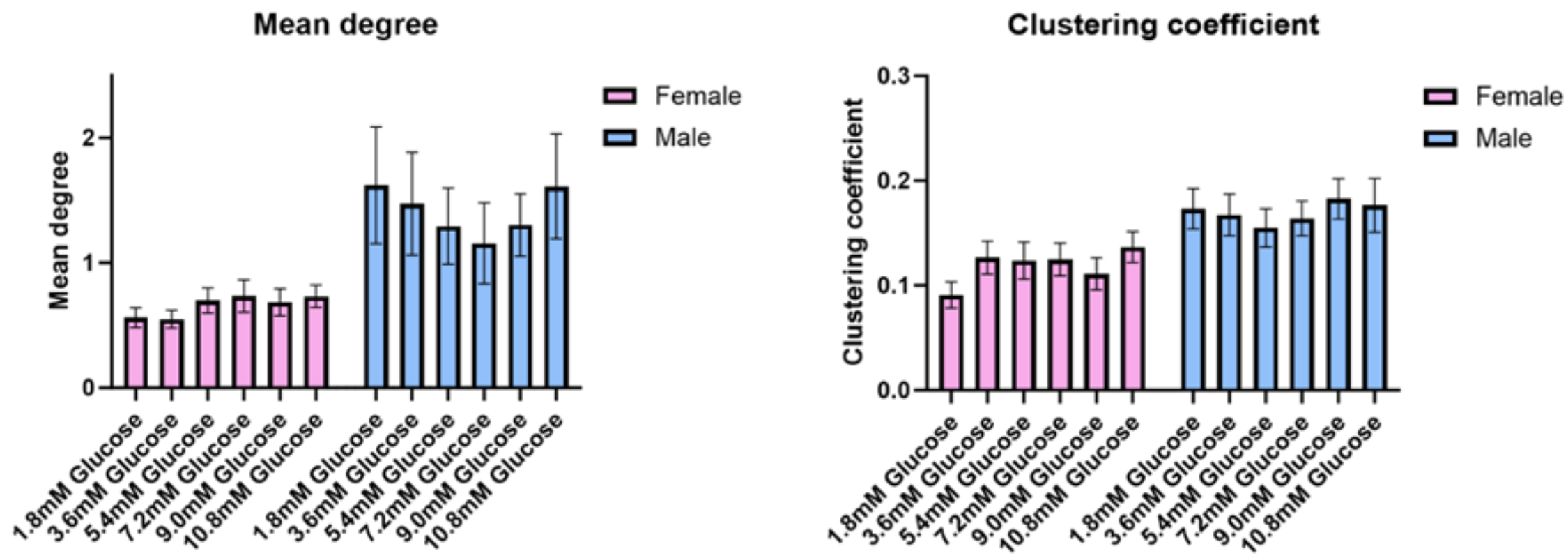

E. C0:0.8

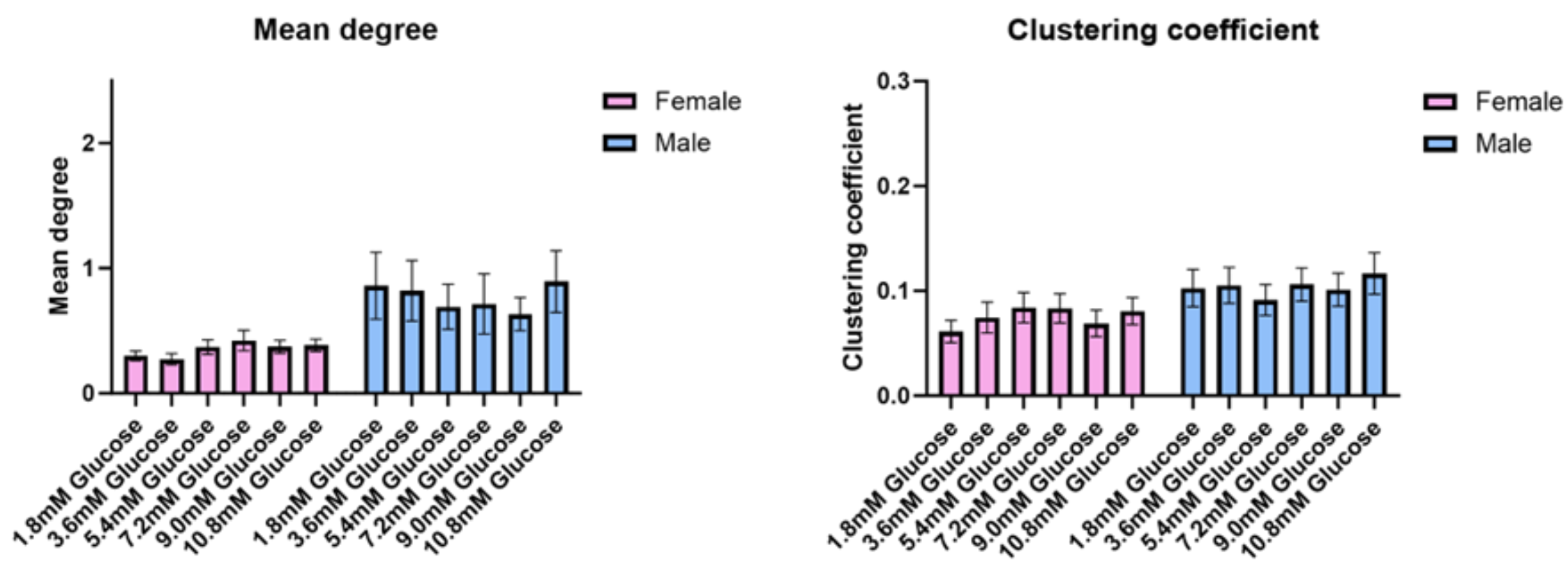

Supplementary data

3.Linking Ca2+ to secretion:
Ca2+ is an accepted proxy, but not a perfect surrogate for hormone output. Consider adding (or discussing) parallel glucagon secretion measurements from matched conditions or cite constraints explicitly as limitations.

> We thank the reviewer for their valuable comment. We have acknowledged this limitation in the Discussion - limitation of the study section, highlighting it as an important area for future methodological development.

Nevertheless, we would like to emphasize that Ca^{2+} imaging remains one of the most informative and widely used approaches for studying islet physiology at *single-cell resolution,* particularly for interrogating islet intercellular communication, which is the primary focus of the present study.

Current technologies that enable simultaneous, single-cell level measurements of hormone secretion in intact islets remain limited or technically challenging. Most secretion assays, including ELISA or perfusion-based approaches, report islet hormone

release only at the whole-islet level. While valuable, these methods lack the spatial resolution required to map hormone output to individual cells or to capture the cell-to-cell interactions that are central to the mechanisms we describe in this study. On the other hand, recent studies using zinc release as a surrogate for that of insulin (PMID: 38278946), as well as earlier studies from our own lab (PMID: 22160693) using a similar approach to achieve single cell resolution, though elegant, do not necessarily report *insulin* release per se (the fusion pore diameter required for the latter being substantially larger than that required for zinc release). This point is now made in the Discussion (page 23-24).

Therefore, while not providing a *direct* measure of hormone secretion, Ca^{2+} imaging does provide the necessary spatial and temporal resolution to investigate the intercellular mechanisms that are central to its control.

4.Delta-cell sampling
Authors note delta-cell heterogeneity and limited sampling. It would help to quantify delta-cell counts per islet or condition and show summary statistics for delta-cell activity bins to support generalized statements.

>We thank the reviewer for this suggestion and have now summarized the number of δ-cells captured per islet per treatment, as shown in Supplementary Table 1.

We emphasize that a more rigorous and substantially larger sampling of δ-cell activity will be required and, ideally, the use of a δ-cell-specific reporter line such as SST-Cre:GCaMP mice, as employed in a recent study by the Huising group (PMID: 40956879). The latter study confirmed our conclusion that δ-cells exhibit heterogeneous Ca^{2+} activity, likely due to variable Cx36 expression.

We did not sub-divide δ-cell activity into bins because, as acknowledged in our manuscript, it was difficult to define a general pattern of δ-cell Ca^{2+} activity in response to glucose ramps within the limited number of δ-cells captured in this study. Therefore, we do not think categorizing δ-cell activity is appropriate as it would require assumptions about stimulus-response relationships which are not experimentally established in the context of our study.

Overall, our study uses the limited δ-cell data primarily in a qualitative, and now descriptively quantitative, manner to suggest δ-cell heterogeneity and to inform follow-up experiments investigating the functional relationship between δ-cells and neighboring α-cells. Indeed, the subsequent pharmacological experiments using SSTR antagonists supported this rationale and demonstrate that inhibition of somatostatin signaling shifts most of α-cells toward a hyperactive state.

5.Sex biology context
The sex-biased effects are compelling. If feasible, please indicate whether estrous cycle

was controlled or logged in females, or state explicitly that it was not and discuss potential impact.

> We thank the reviewer for highlighting the potential effect of the estrous cycle in female mice on our experimental outcomes. Estrous cycles were not controlled or logged in female mice in our experiments. This is because, based on previously published work (PMID: 19608983), female mice have a higher estrogen level than a male mouse even during the low-estrogen phases of the estrous cycle. Specifically, female estradiol (E2) levels fluctuate from approximately 60pg/ml during proestrus to 17pg/ml during estrus and diestrus. In comparison, the typical estrogen level in male mice is much lower, around 12 pg/ml, which is below female levels even during estrus and diestrus. Therefore, we believe that the potential impact of estrogen is likely consistent throughout the estrous cycle of the female mice. We have discussed this point in the Discussion (page 19) per the reviewer's suggestion.

6.GCaMP6f "leakage" into beta-cells
About 10% of GCaMP regions were insulin positive. The authors restrict alpha-cell analyses to dual-positive glucagon+GCaMP ROIs, which is appropriate. Still, a brief control showing that key results are unchanged when excluding border or ambiguous ROIs would strengthen confidence.

> We appreciate the reviewer's attention to a potential confounding effect of GCaMP6f expression outside of α-cells, and agree that careful cell identification is important. However, we would like to note two reasons why further restricting the dataset by removing ROIs adjacent to β-cells would not be appropriate.

First, all ROIs included in our analysis were required to be clearly glucagon-positive based on high-quality immunostaining, and ROI boundaries were defined using our validated automated segmentation pipeline, which yields stable, well-separated cellular outlines (PMID: 36449570), minimizing uncertainty in ROI identity or boundary placement.

Second, in intact mouse islets, α-cells are commonly intermingled with, or immediately adjacent to, β-cells. Therefore, excluding border ROIs would introduce substantial sampling bias by disproportionately removing a large portion of α-cells, potentially excluding multiple naturally occurring α-cell subtypes.

For these reasons, we believe that additional exclusion of border ROIs would not strengthen our confidence in cell identity and could instead skew sampling away from physiologically representative α-cell populations. We have clarified these criteria in the Methods-Classification of α-cells based on Ca^{2+} activity patterns.

Reproducibility
Core analysis code is publicly available on GitHub. Please add commit hashes and a minimal “runbook” - inputs to outputs and example datasets or HDF5 traces sufficient to re-run a figure-level analysis. Also, please state the software versions used for network metrics.

> We thank the reviewer for this inquiry. The core analysis code is publicly available on GitHub. Full reproduction of the published figures, however, requires access to the private server environment at the Medical University of Vienna, which is available only on request and is currently used by 10 laboratories worldwide.

While we are unable to provide a full runbook or example datasets sufficient for complete figure-level reproduction outside this environment, the public code allows readers to inspect and test the analysis workflow. The software versions used to reproduce the figures are:

Python 3.10.10 | packaged by conda-forge | (main, Mar 24 2023, 20:08:06) [GCC 11.3.0]
networkx 3.0
numpy 1.23.5
scipy 1.10.1
pandas 1.3.5

The code can alternatively be downloaded and installed on a user’s own virtual machine, though exact reproduction of the published figures requires the server setup. All commits in the repository are fully functional within the server environment, and the listed commit used for this manuscript is:
d81961bf20e7005197dc9ffac706280492ad4b80.

Minor comments
•Report exact drug pre-incubation times and wash-in kinetics for S961 in the main Methods (which is currently scattered).

> It is now added (line 112) in the Method section in the revised manuscript.

•Standardize units across figures (e.g., glucose mM; event/min/ROI).

> We thank the reviewer for noting this inconsistency. Units in all figures have been standardized: glucose in mM and frequencies as events/min/ROI.

•Where possible, include mouse-level n alongside islet-level n in all panels.

> We thank the reviewer for this suggestion. We would like to note that mouse-level *n* values were already included alongside islet-level *n* in all applicable figure panels in the original manuscript.

Additional limitations that should be noted
Because SSTR2/3 and InsR antagonists were applied globally in intact slices, observed alpha-cell changes may partially reflect indirect effects via beta- and delta-cells that also express these receptors. Authors need to discuss this explicitly. Also, while mouse slices are physiologically relevant, confirmation in human pancreas with its higher alpha and beta cell intermix will be important for human translation. Given the authors' acknowledgment of alpha-cell molecular heterogeneity, a brief note on how this could underlie different Ca^{2+} phenotypes would round up the limitation section.

> We thank the reviewer for highlighting this important limitation. These aspects were, in fact, discussed in the original version of the manuscript:

Line 476: Discussion of the limitation associated with the global application of SSTR2/3 and InsR antagonists in pancreatic slice experiments.
Line 489: Acknowledgment of the need for future validation of our findings in human pancreatic tissue.

Line 486: In response to the reviewer's comment, we have included a note highlighting the potential contribution of α-cell transcriptomic and proteomic heterogeneity to the different Ca^{2+} phenotypes observed in our study.

Reviewer #2 (Remarks to the Author):

Huang et al studied islet cell Ca2+ activities (proxy for hormone secretion) in pancreatic slices focusing on glucagon- releasing alpha cells and their dependence on neighbouring insulin and somatostatin secreting beta and delta cells, respectively, when the slices were exposed to gradually increasing glucose concentration. They found that the alpha cell responses during a glucose ramp were markedly heterogenous, but in general in opposite phase to or inversely correlated to the beta and delta cell responses. Exposure to inhibitors of insulin receptors or somatostatin receptors 2+3 strongly activated most alpha cells and shifted their inhibition by glucose to higher concentrations in parallel with a similar shift in the onset of beta cell Ca2+ activity. Female alpha cells were more sensitive to somatostatin receptor inhibition and male alpha cells to insulin receptor inhibition. However, when insulin and somatostatin receptor inhibition were combined the alpha cells showed increased Ca2+ activity but the sensitivity to glucose remained. The authors speculate about the role of intra-islet paracrine coordination in the tuning of alpha cell glucose responses and the potential importance of the observed sex differences in the development of diabetes. The manuscript is not easily digested. There is plenty of data and the text does not follow the Figures numbers. Instead, one has to jump back and forth between Figures and conditions. I think the manuscript could be considerably improved in this respect. The study confirms previous observations of alpha cell heterogeneity and that they are glucose-regulated both via paracrine interactions and intrinsic effects. The major novel aspect is the somewhat unexpected differences between sexes. I find the study interesting but raise a number of issues for the author's consideration.

> We would like to thank the reviewer for having read our manuscript carefully and for their suggestion regarding figure rearrangement. To further enhance readability and improve alignment between the text and figures, we have revised specifically Figure 3. Male and female islets under the same treatments are now presented side-by-side, while preserving the original experimental sequence and logic. We would like to note that although our figures contained a substantial amount of data, we have carefully arranged them to present the results in the clearest and most logical sequence possible (as the reviewer is no doubt aware this is always a little challenging with a complex dataset). We hope, nonetheless, that the revised arrangement provides a clearer and more intuitive flow for the reader.

We would also like to emphasize that, while our findings confirm certain aspects of previous work, the present study provides substantial new advances to the field.

Notably, we present a precise (GluCre-driven GCaMP6f-expression) and comprehensive large-scale characterization of Ca^{2+} dynamics selectively in α-cells in response to physiologically relevant glucose ramps spanning a broad range (1.8-10.8

mM) of gradually increasing glucagon concentrations. In addition, by combining genetically encoded and chemically loaded Ca^{2+} indicators in live, freshly-prepared pancreatic slices, we establish a robust experimental platform that enables islet cell-type-resolved Ca^{2+} imaging of α-, β-, and δ-cells within the intact islet microenvironment. This approach allows direct, simultaneous interrogation of coordinated activity among multiple endocrine cell types. Furthermore, by integrating this imaging strategy with targeted pharmacological dissection of SSTR2/3 and insulin receptor signaling, along with explicit analyses of sex-dependent differences, we provided a level of mechanistic insight and physiological relevance that has not been previously achieved. These features, as provided in our study, stand in remarkable contrast to earlier studies, which largely relied on sparse (viral) labeling or analyses restricted to single islet cell types, employed large stepwise increases or decreases in glucose concentrations (that are less consistent with normal physiological changes) or involved studies in whole islets previously exposed to the potentially deleterious effects of the isolation process, and denuded of interactions with non-islet cells. Therefore, we believe that the insights provided by our study substantially enrich the field and will inform both future mechanistic investigations and the development of more targeted therapeutic strategies for diabetes.

Major issues.

1. In the introduction and later in the manuscript "synchronized beta cell Ca2+ oscillations" are mentioned. In other instances, "synchronized beta cell Ca2+ activity" and "synchronized Ca2+ activation" are used. Extremely well synchronized beta cell oscillations have been shown in many studies but synchronization in the present traces is far from impressive although the beta cells show higher connectivity than the alpha cells.

>We thank the reviewer for this comment. To address the concern, we have removed the term *"synchronized"* throughout the revised manuscript.

Our β-cell recordings appeared less impressive because the fluorescence signals obtained from β-cells (as well as δ-cells) were from a small molecule Ca^{2+} indicator (Calbryte 590) loaded into the cells, for which the recording quality depends on efficient dye loading, imaging depth, and cellular uptake efficiency. In contrast, the α-cells in our mouse model genetically express GCaMP6f, which provides superior and clearer fluorescence signals. Typically, the very top layers of islet cells in pancreatic slices offer the best resolution for Calbryte 590-loaded cells such as β-cells (as well as all islet cells on the same plane), as they were directly exposed to the incubation medium containing the Ca^{2+} dye during loading. However, when recording GCaMP6f signals from α-cells, we often found that a slightly deeper focal plane (~3 μm below where β-cells signals appeared optimal) yielded more optimal resolution. Therefore, to achieve the best

possible imaging quality across all islet cell types, either GCaMP6f-expressing or Calbryte 590-loaded cells, we had to balance these slightly different focal planes, which resulted in a modest compromise in the β-cell recordings.

We would like to note that although the β-cell recordings were not optimized for maximal Ca^{2+} signal intensity, they were fully sufficient for resolving spatiotemporal Ca^{2+} dynamics at the resolution required for interrogating α-cell activity and its association with neighboring β-cells within intact islet architecture, which is the main aim of our study.

2. The authors acknowledge the weakness in using Ca2+ as a proxy for secretion. This weakness is probably accentuated by focusing on Ca2+ oscillation frequency. The Ca2+ patterns are thus very different in different cells with short-lived spiking in some, more prolonged oscillations and even sustained elevation in others. A better proxy for secretion is therefore probably area under the curve, when comparing different conditions in individual cells.

>Re: We thank the reviewer for their suggestion.

In our preparation, the activity of α-cells (as well as β- and δ-cells) is dominated mostly by short-lived Ca^{2+} oscillations rather than sustained Ca^{2+} elevations. For this reason, we decided to use the frequency of Ca^{2+} events as a more appropriate proxy for hormone secretion.

Over the course of the glucose concentration ramp which we applied to the pancreas slices, α-cells and δ-cells typically exhibited short-lived spike activities whereas β-cell activation begins around the midpoint of a 10-minute perifusion at 7.2 mM glucose, followed by an initial phase of Ca^{2+} oscillations characterized by high-frequency activity. Over time, these oscillations transition into a sustained phase composed of slower oscillations superimposed with fast-frequency activity. This sustained phase was generally observed around 10.8 mM glucose, although its onset varied, occurring sometimes early, but mostly late, or not at all within the 10-minute perifusion period.

When subjected to pharmacological manipulations, α-cells and δ-cells cells showed obvious changes in Ca^{2+} oscillation frequency without major alterations in the overall mean width or amplitude of Ca^{2+} transients (unpublished observations). Whereas for β-cells, the onset of activity was frequently delayed until the achievement of higher glucose concentrations, resulting primarily in fast-frequency Ca^{2+} oscillations.

We previously demonstrated that in tissue slices, fast, short-lived Ca^{2+} oscillations in β-cells are glucose concentration dependent, whereas slow oscillations did not exhibit such glucose dependence (PMID: 33833686). Furthermore, oscillations in intracellular Ca^{2+} concentration (leading to repetitive Ca^{2+} increases, and in contrast to sustained

Ca^{2+} elevations) in islet endocrine cells are the key determinant of the probability (and thus the rate) of granule exocytosis (PMID: 8145165; PMID: 10092049). This is likely because oscillatory increases in intracellular Ca^{2+} are essential for activating the local exocytotic machinery, preventing desensitization, and supporting vesicle pool replenishment (PMID: 10092049), ultimately promoting effective hormone release.

For reasons above, the Ca^{2+} oscillation frequency is, in our view, a more reliable proxy for hormone secretion than the total signal magnitude (AUC). The latter frequently masks the underlying kinetics of exocytosis and is susceptible to variability from technical factors such loading efficiency of the dye (i.e., Calbryte 590) between islets or between individual cells or in the expression levels of Ca^{2+} indicators (i.e., GCaMP), as well as differences in the tissue depth of the imaging plane, leading to fluctuations in the signal-to-noise ratio between samples. These factors can introduce substantial data variability in AUC measurements that obscure physiologically meaningful changes in secretion. Hence, we prefer to report Ca^{2+} spike frequency rather than AUC as a more relevant proxy for hormone release.

3. As stated above some data are confirmatory and it would be appropriate to cite earlier findings of alpha cells heterogeneity, e.g. alpha cells with beta cell-like glucose sensitivity (PMID: 27539300).

>Re: Thank you for the suggestion. This article is now cited (ref 20).

4. The observed Ca2+ patten might also be influenced by the indicator used. It would therefore be valuable to compare the GCaMP6f responses of the alpha cells with their Calbryte 590 responses.

> Thak you. As now shown below, we compared α-cell Ca^{2+} dynamics measured with GCaMP6f and Calbryte 590 in pancreas slices in response to the reviewer's request.

Both indicators reported qualitatively similar Ca^{2+} dynamics (Fig. A and B). Quantitative analysis of signals extracted from α-cells in three islets (Fig. C) showed that GCaMP6f signals exhibited slightly higher event frequencies and larger amplitudes, along with modestly shorter event half-widths, and area under the curve. Comparison between the width of α-cell Ca^{2+} events extracted from GCaMP6f and Calbryte 590 signals were also performed (Figure E).

We would note that GCaMP6f has a higher Ca^{2+} dissociation constant (Kd) than the chemical indicator Calbryte 590. Consequently, in cells that both express GCaMP6f and are loaded with Calbryte 590, such as the case of α-cells in our study, GCaMP6f is, in theory, expected to bind and retain Ca^{2+} for longer periods, thereby altering the apparent kinetics and amplitude of Calbryte 590 signals relative to its intrinsic properties. Nevertheless, while different indicators may influence absolute signal

characteristics, they do not affect the relative temporal patterns, coordination, or intercellular relationships that form the basis of our conclusions. All α-cell analyses in our manuscript have been performed using a single, genetically-encoded indicator (GCaMP6f), ensuring internal consistency across experiments.

Lastly, we note that since we did not find the analysis shown below necessarily meaningful for the aims of our study, which focus on α-β (as well as α-δ) cell interactions rather than indicator performance, it is not included within the revised manuscript.

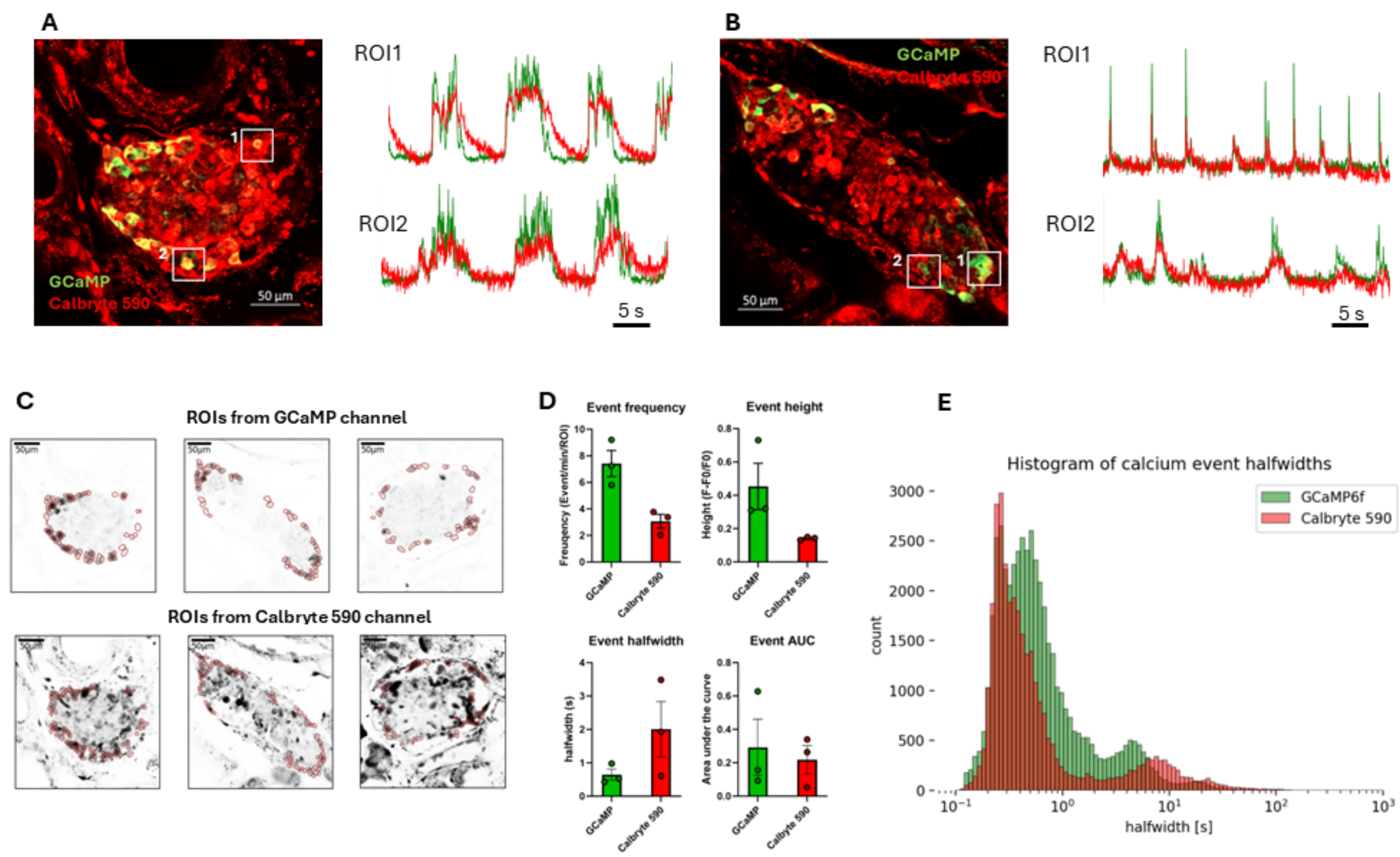

Figure. A–E. Ca^{2+} events in α-cells monitored with GCaMP6f and Calbryte 590.
A–B. Example Ca^{2+} events recorded from α-cells under 3.6mM glucose condition. The left panels show islet images with inset regions (1 and 2) highlighting individual α-cells expressing GCaMP6f and loaded with Calbryte 590. The right panels show the corresponding fluorescence traces extracted from these α-cells. GCaMP6f signals are shown in **green**, and Calbryte 590 signals in **red,** recorded concurrently.
C. α-cell ROIs within 3 islets were identified in the 488 nm channel (GCaMP6f; top panel) and the 561 nm channel (Calbryte 590; bottom panel).
D. Quantitative analysis of Ca^{2+} event properties from the islets shown in **C.**, including event frequency, amplitude, half-width, and area under the curve recorded during the 3.6 mM glucose phase of a glucose ramp.
E. Histogram distribution of event halfwidths for all Ca^{2+} events extracted from α-cell ROIs in panel **C.** during a glucose ramp, measured from both GCaMP6f (green) and Calbryte 590 signals (red).

5. The confocal imaging was done in a single plane of 140 µm thick pancreatic slices 10-14 cell layers. How deep into this slice was this plane? I suppose that different cell types were only identified within this plane? Please clarify and discuss potential influence from not identified cells in other planes.

>Re: We thank the reviewer for making this point highlighting this potential limitation. Our recording typically lay within 0-20 µm from the surface of the tissue slice, where

visualization of all islet cell types was most optimal. This limitation was recognized by us but was not elaborated upon in the original manuscript due to space constraints and the prioritization of the main findings. We have now expanded the discussion of this limitation in the revised version of the manuscript.

6. Line 171: “Counterintuitively”, well, not necessarily since glucagon amplifies insulin secretion.

>We have removed the word “Counterintuitively” in the revised manuscript.

7. When examining the Ca2+ dynamics between delta and alpha/beta cell pairs an inverse relationship between delta and adjacent alpha cells was observed. However, in Fig 2E-1 the alpha and beta cells remain essentially inactive independent of delta cell activity. Moreover, the activity of the delta cell declines with increasing glucose concentrations (also in Fig 2-F) although somatostatin secretion is characteristically stimulated by increasing glucose. These delta cell Ca2+ responses seem more like what might be expected from alpha cells. Moreover, I cannot follow the authors argumentation about Fig 2F-2 (lines 225-226) “with δ-cell activity resuming (7.2–9.0 mM glucose; Figure 2F-2) as α-cell activity declined”. First, the alpha cell activity declined already in 5.4 mM glucose and the resuming δ-cell activity is unconvincing.

>We thank the reviewer for these important and detailed observations. In Fig 2. E-1 and 2E-2, we aimed to illustrate that when a δ-cell is active, a neighboring α-cell is often quiescent (Fig. 2E-1; Supp Fig. 3A), whereas when an α-cell is active, the neighboring δ-cell is frequently inactive (Fig. 2E-2; Supp Fig. 3A). This inverse relationship is also evident temporally: periods of δ-cell activity are often accompanied by reduced Ca^{2+} oscillations in neighboring α-cells (Fig 2. F-1; Supp Fig. 3A), and vice versa (Fig2. F-2; Supp Fig. 3A). These observations led us to hypothesize that δ-cells may regulate the intensity of α-cell activity, which we subsequently tested using pharmacological blockers. We apologize for the previous use of the term “resuming,” which has now been corrected to “activating” for clarity.

8. In lines 228-230 the authors point out the lack of correlation between delta and beta cell activity. This is not unexpected below the glucose threshold for beta cell activation but it would be appropriate to discuss the lack of correlation at higher glucose concentrations since pulsatile insulin and somatostatin secretion have been found to be essentially in phase (e.g. PMID: 22227186). This may be added to the discussion about beta-delta cell gap junction coupling (around line 385).

> We thank the reviewer for the suggestion and have now extended our discussion on the lack of synchronized Ca^{2+} activity between δ- and β-cells in the revised manuscript (page 21, line 406-412).

9. Although it is stated on lines 325-326 that the somatostatin receptor subtypes-2 and 3 were blocked alone, or both, I think they were only blocked together. This is a pity since blocking the receptors separately would have allowed conclusions about separate effects on alpha and beta cells. It is stated in lines 269-270 “SSTRA-supplemented glucose ramp, β-cell Ca2+ activity frequency was increased at basal glucose concentrations (1.8-5.4mM glucose in both sex; Figure 4 β-cells: SSTRA treatment)”. One wonders how this activity (events/min Fig 4) is defined when comparing Figs 3-1A and B. This is related to issue 2 above. The only thing that is very apparent is the onset of beta cell activity at the higher glucose concentration. Returning to the role of the different SSTRs: are the effects of SSTRAs on beta cells due to inhibition of their SSTRs or indirect and related to increased glucagon secretion after blocking the alpha cell SSTRs? This is one issue that might have been resolved by separate receptor SSTR blocking and further clarified with antagonists of glucagon action (glucagonR and GLP-1R blocking).

> We apologize for the ambiguity. In our manuscript, “both” refers to the combined blockade of insulin receptor signaling and somatostatin receptor (SSTR2/3) signaling. We have clarified this in line 366 as follows: “…by blocking insulin receptor signaling, somatostatin receptor subtypes 2 and 3, *or both pathways simultaneously*.”

We respectfully disagree that blocking SSTR2 or SSTR3 individually would allow clear conclusions regarding their separate effects on α- and β-cells. As reported by the Huising and Gribble groups (PMID: 27408771 and PMID: 27390011), and the images below,

SSTR

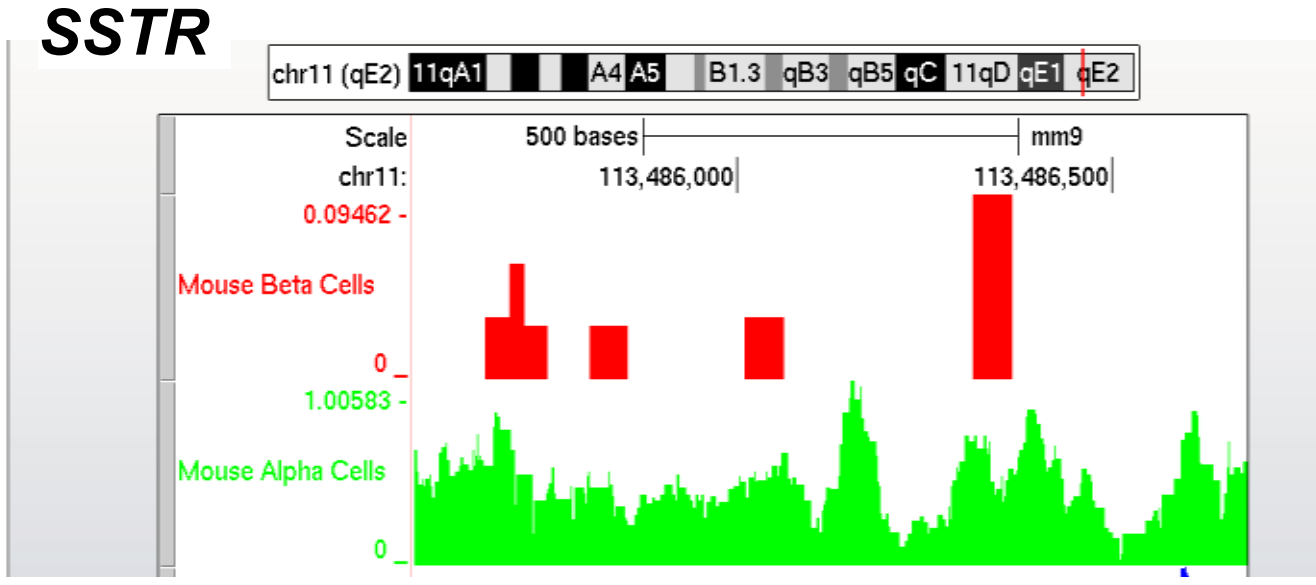

SSTR3

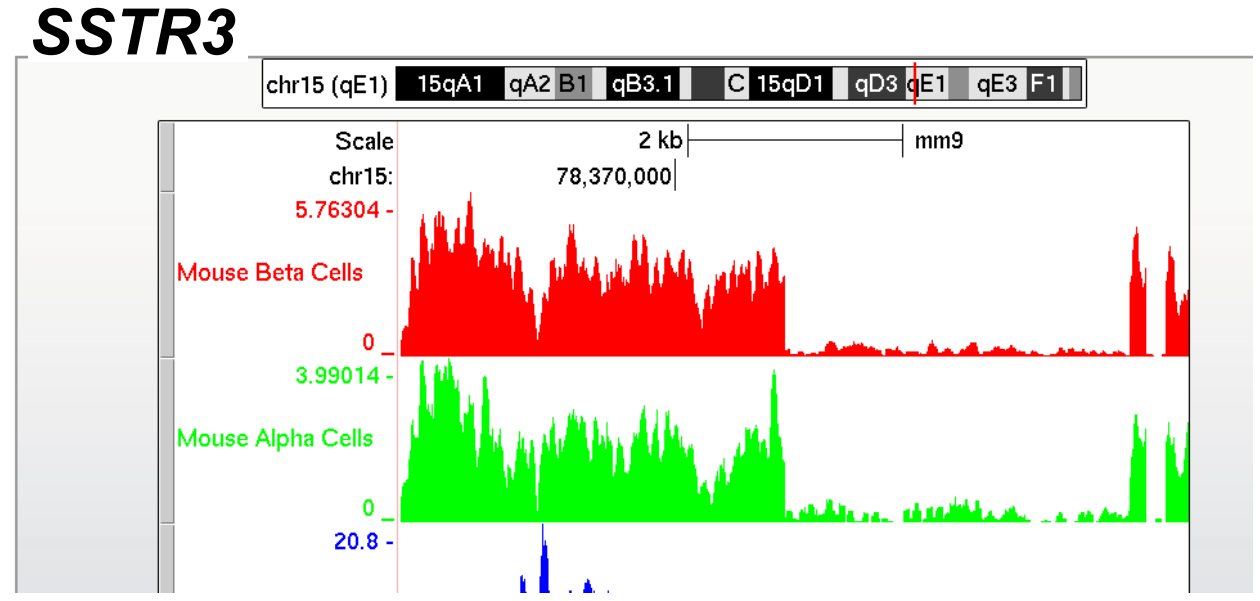

while **α-cells** selectively express **Sstr2**,
Sstr3 is also expressed in **α-,** β-, and δ-cells. Therefore, our rationale for blocking both SSTR2 and SSTR3 was to ensure complete inhibition of somatostatin receptor–mediated signaling in α-cells. Although the use of an SSTR3 antagonist would also block somatostatin signaling in β- and δ-cells, we have acknowledged this as one of the limitations of our study in the Discussion section. Our data, nonetheless, reflect the integrated physiological behavior of the islet when somatostatin signaling is removed within the islet.

Regarding the reviewer's question about the effects of SSTRAs on β-cells, the observed effects on β-cells in our study could arise from either direct inhibition of β-cell SSTRs or indirect mechanisms secondary to increased glucagon secretion following blockade of α-cell SSTRs. Our data would not allow us to distinguish between these two possibilities. We value the reviewer's suggestion that by including the glucagon receptor (GCGR) or GLP-1 receptor (GLP-1R) inhibitors would help determine whether the increased β-cell activity observed after SSTRA treatment reflects direct somatostatin receptor blockade or indirect enhancement of glucagon signaling. However, since the primary objective of our study was to investigate α-cell regulation within the islet rather than β-cell signaling mechanisms, we do not expect that performing the suggested GCGR or GLP-1R inhibitor experiments would alter the main conclusions of our work. Nevertheless, we have incorporated this valuable suggestion into the Discussion as an important direction for future work (page 22, Line 466).

10. A particular peculiarity is the right-shifted glucose b cell threshold after SSTRA. As discussed in lines 394… removal of SST signalling has previously been found to lower the b cell glucose threshold. A lowering of the threshold makes more sense to me considering the amplifying action of glucagon/cAMP on insulin secretion. The authors also make a point of the observation that a decline in alpha cell Ca2+ activity is associated with the appearance of "synchronized" beta cell activity (lines 271-273). This does not make sense considering the just mentioned amplifying effect of glucagon on insulin secretion. Again, experiments with separate SSTR subtype antagonists and glucagonR and GLP-1R blocking may shed light upon the discrepancy with previous data.

> We emphasize that the "decline in α-cell Ca^{2+} activity is associated with the appearance of 'synchronized' β-cell activity" to highlight our observation of the antiphase relationship between α- and β-cell Ca^{2+} oscillations. In our experiments, we consistently observed delayed activation of β-cell activity when α-cell Ca^{2+} activity was potentiated, either by SSTRA or S961, likely reflecting this antiphase relationship. We must respectfully disagree with the reviewer since, as addressed in Reviewer Comment #9, separating SSTR subtype antagonists would probably not help dissect the route of inhibition of somatostatin on β-cells, given the expression of SSTR3 in α-, β-, and δ-cells. Most importantly, the focus of the current study is to decipher the complex regulation of α-cells, rather than β-cells, with or without insulin and/or somatostatin signaling. We have acknowledged the potential value of future experiments using glucagonR or GLP-1R inhibitors, as pointed out by the reviewer to further explore β-cell regulation under these conditions in the revised manuscript; however, we think that performing such experiments, which would require up to a year's further work, is unlikely to alter the main conclusions of the study.

11. Referring also to issue 2 above about Ca2+ as proxy for secretion I wonder what the significance is of the "significantly blunted Ca2+ oscillation frequency at >9 mM glucose" in beta cells and why this is "a result of the delayed onset of their synchronized Ca2+ activation during the glucose ramp" (lines 275-277). Incidentally, "synchronized" seems correctly used in the latter sentence since is refers to the onset of Ca2+ activation.

> Although we have acknowledged in the "Limitations of the study" section that Ca^{2+} dynamics may not fully capture hormone secretion, islet-wide intracellular Ca^{2+} dynamics remain a widely used and informative proxy for assessing cellular activity, particularly for studying islet intercellular communication (PMID: 33833686; PMID: 36084321; PMID: 34190340).

Regarding our statement that Ca^{2+} oscillation frequency was "significantly blunted at ≥9 mM glucose," this refers to **the mean oscillation frequency of β-cells at 9 mM glucose** and above, as shown in Figure 4 β-cells. Under control conditions, mean β-cell Ca^{2+} oscillation frequency increased at 7.2 mM glucose and above in both males and females. However, in the presence of S961 or/and SSTR antagonists, β-cell activation is shifted to higher glucose concentrations, with the exact activation threshold varying across slices, resulting in significantly reduced **mean** Ca^{2+} oscillation frequency at 9 mM glucose and above. Please note that this is an observation due to a **"shift"** of the inhibition and activation threshold of α and β-cell, respectively.

12. Referring to issue 9 above there is a similar peculiarity in the effect of the insR inhibitor in associating the decrease of alpha cell activity at higher glucose concentration with the appearance of "synchronized beta cell Ca2+ oscillations".
> Please refer to response for issue 11.

13. Again referring to issues 2 (about Ca2+ as proxy for secretion) and 10, I wonder what the significance is of the blunted Ca2+ oscillation frequencies at >9 mM glucose in response to insR inhibitor (line 304).
>Please refer to response for issue 11.

14. The inverse relationships between alpha and delta cell activities under insR inhibition is not particularly convincing (lines 307-308, Fig 6B).

> We thank the reviewer for raising this point. To address the concern, we performed additional analyses for Figure 6B (below), similar to the approach used in Supplementary Figure 3A. These new analyses highlight the often-opposite trends between the mean active fraction of α-cells and δ-cells.

Figure 6B Female-top islet

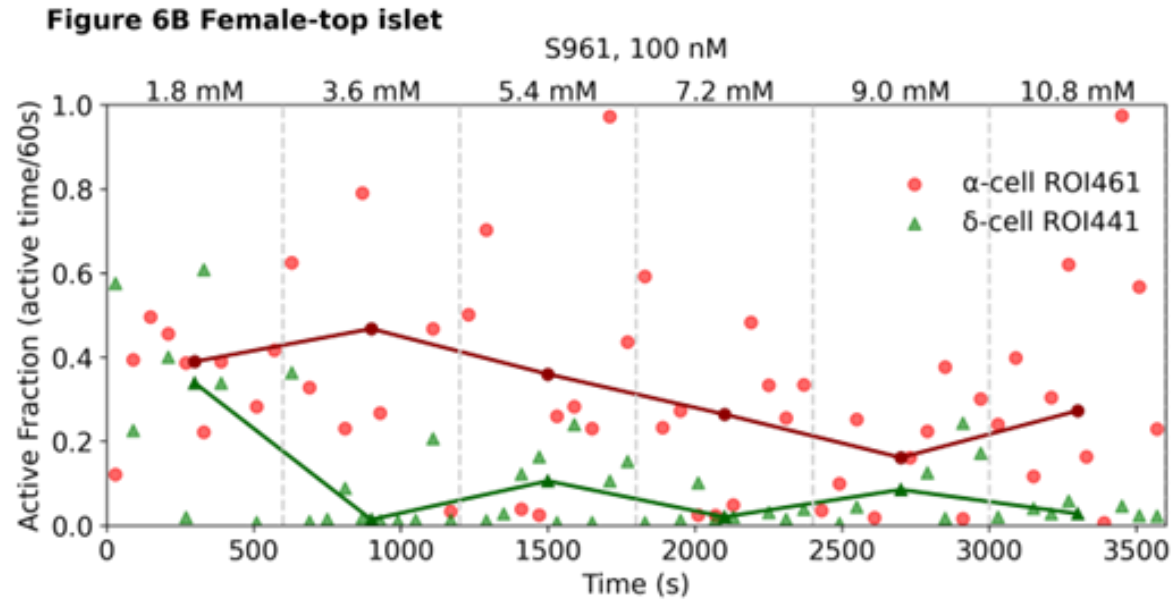

Figure 6B Male-top islet

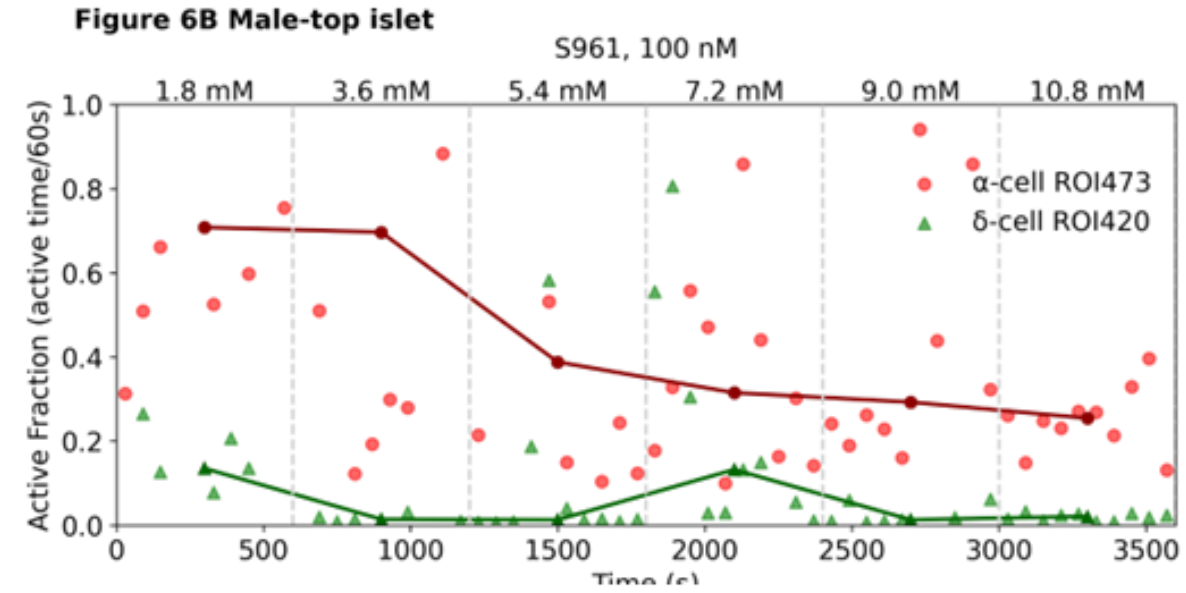

Figure 6B Male-top islet

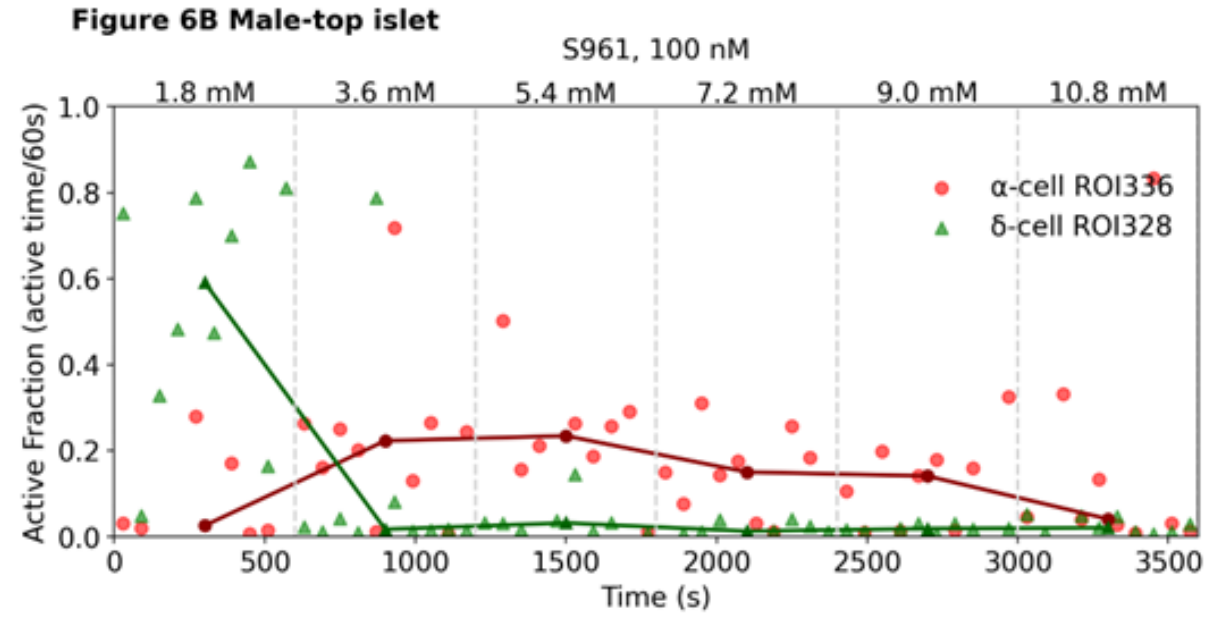

Figure 6B Female-bottom islet

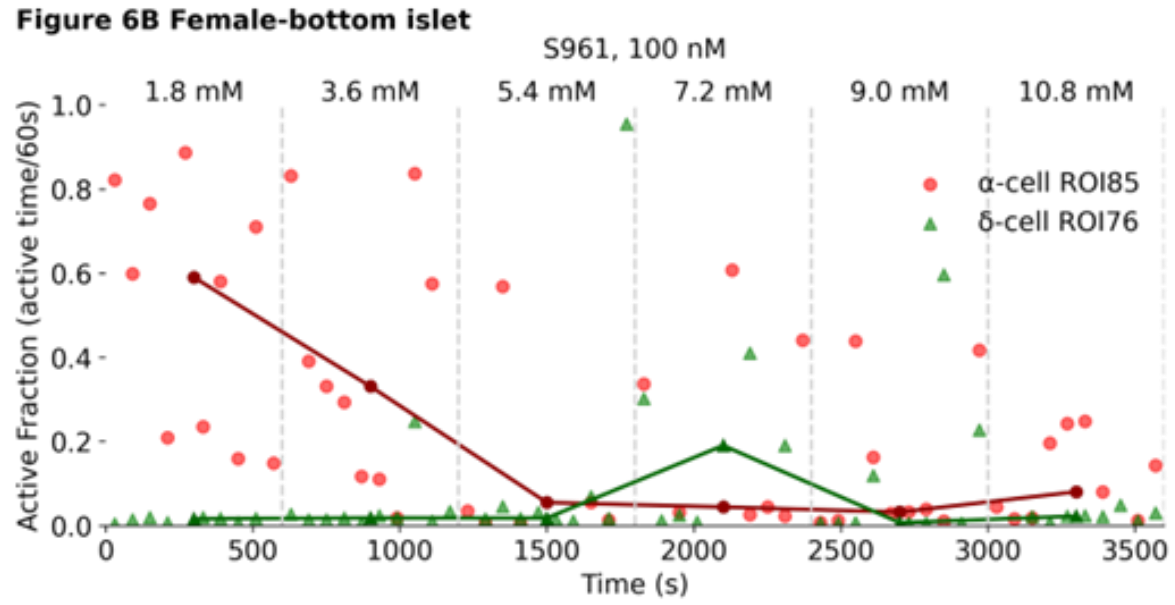

Figure 6B Male-bottom islet

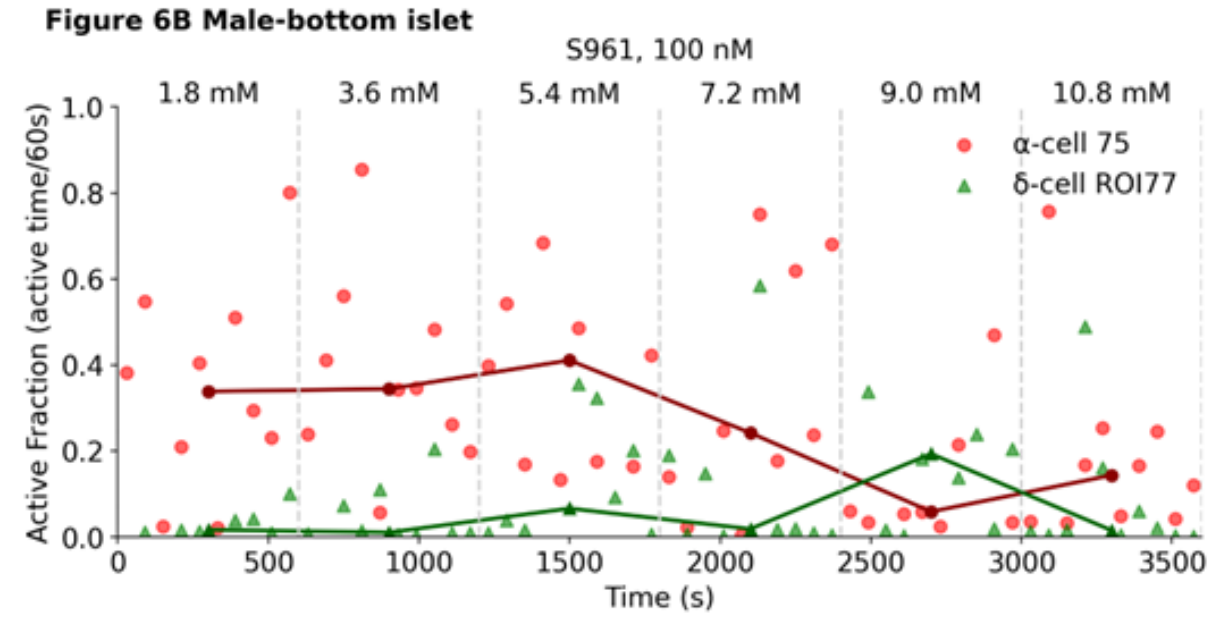

Figure 6B Female-bottom islet

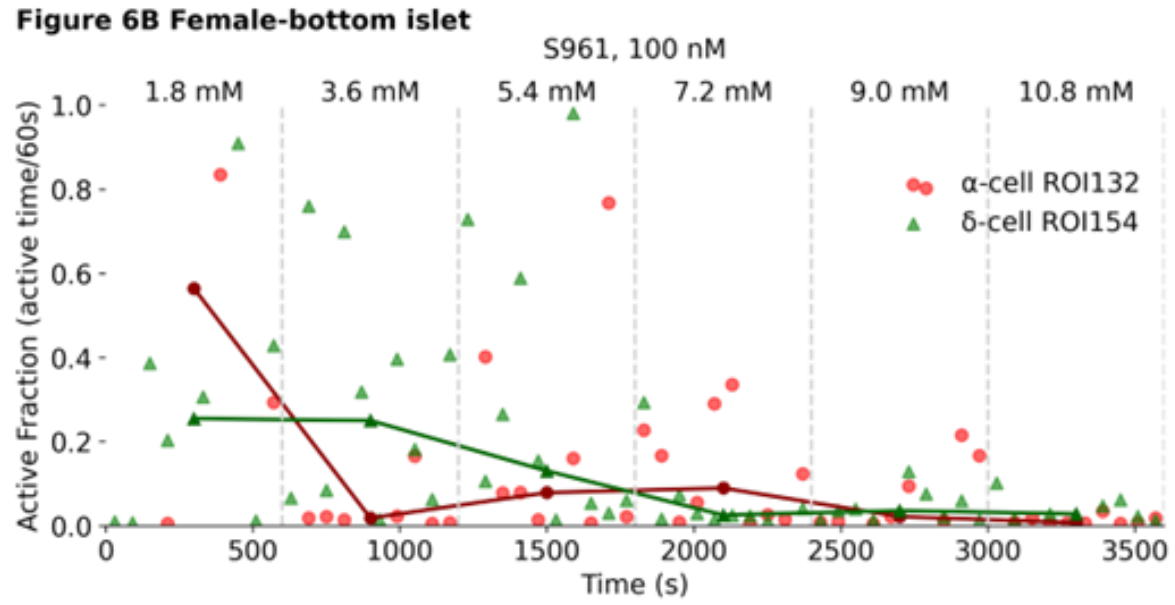

Figure 6B Male-bottom islet

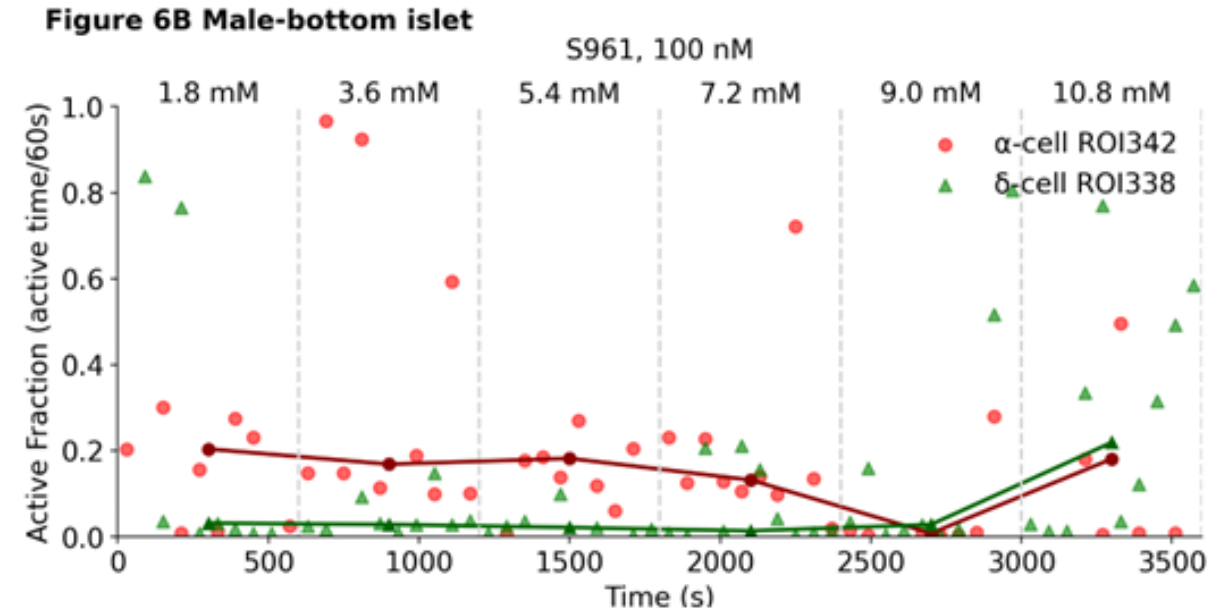

Figure 6B Male-bottom islet

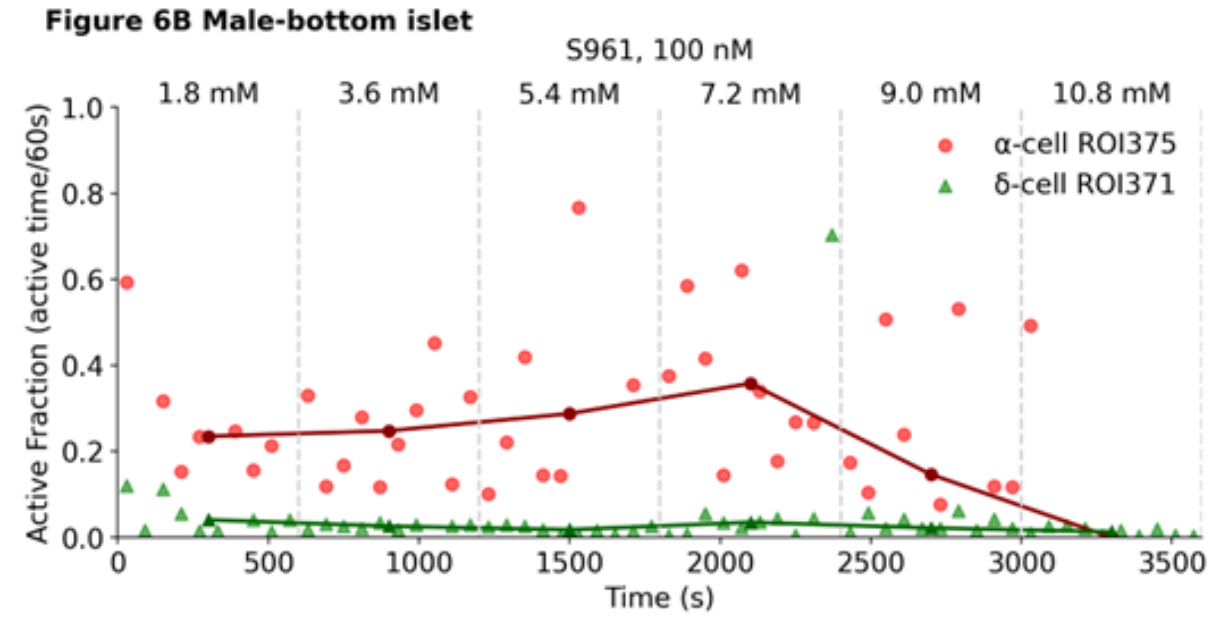

15. The statements in lines 356-358 that glucagon agonism or glucagon-GLP-1 co-agonism exerts antidiabetic effects and improves glucose metabolism seem inconsistent with the present findings that SSTR and insR antagonism right shift the glucose threshold for glucose activation of the b cells despite maintaining alpha cell activity at higher glucose concentrations.

>The antidiabetic effects of glucagon agonism or glucagon-GLP-1 co-agonism described in the original manuscript refer to *systemic metabolic outcomes* demonstrated in *in vivo* studies, where glucagon receptor activation enhances energy expenditure, improves lipid metabolism, and acts synergistically with GLP-1 signaling to lower body weight and improve glycemic control. These mechanisms are distinct from the *acute intra-islet paracrine interactions* examined in our study.

In contrast, our present findings show that *antagonism of SSTR or insR* alters the intra-islet signaling environment by sustaining α-cell Ca^{2+} activity at higher glucose levels. Under these conditions, elevated α-cell activity can shift the β-cell glucose activation threshold to the right, reflecting a local paracrine effect rather than the systemic actions associated with pharmacological glucagon agonism.

To avoid confusion, we have revised the text to clarify that the cited antidiabetic effects arise from **whole-body metabolic pathways**, whereas our data focus on *local intra-islet signaling dynamics* that may acutely modulate β-cell glucose responsiveness.

Minor issues.

16. The term “immunoassay” is repeatedly used when the authors apparently mean “immunostaining”, which is also used. I think that the latter more precisely describes what was done.

> It is now corrected to immunostaining throughout the text.

17. Line 179: Should probably be “express” rather than “expressing”.

> We thank the reviewer for pointing out this typo, which has now been corrected.

18. Lines 270 and 276: Should probably be “sexes” rather than “sex”

> We thank the reviewer for pointing out this typo, which has now been corrected.

19. Figure 4 beta cells: It is difficult to follow the lines indicating significances.

> We thank the reviewer for pointing this out. The lines are now modified according to the new analyses suggested by Reviewer 1.

Reviewer #3 (Remarks to the Author):

The manuscript by Ya-Chi Huang et al. utilizes pancreatic slice calcium imaging to examine islet cell responses from male and female mice to secretagogues and in relation to neighboring cells. The authors find that alpha-cell Ca^{2+} oscillations were heterogeneous and inversely correlated with delta-cell calcium responses. The manuscript also finds that blocking insulin receptors (with S961) and/or somatostatin receptors increased the excitability of alpha-cells slightly differently in male compared to female alpha-cells. The manuscript further identified differences in alpha-cell responses as well as heterogeneity of these responses in male versus female islets. As pancreatic slice technology maintains the islet microenvironment, the data are physiologically relevant. The data is of high quality and improves the understanding of islet cell calcium handling, paracrine signaling, and sex differences of islet cell calcium responses. There are some minor areas that require attention that are detailed below:

The authors find that, “Remarkably, Ca2+ dynamics in α-cells were always in antiphase with the nearby insulin-positive β-cells (Figure 2D-1 to Figure 2D-3), aligned with earlier findings39.” However, the manuscript also finds that glucose inhibits calcium in many of the alpha cells. Thus, some discussion about the differences observed from the findings in reference 39 is warranted. It would also be nice to see a zoomed view of some of the antiphase firing from one islet especially “in α-cells at different islet regions (Figure 2D) exhibited asynchronous patterns of Ca2+ activities.” It is interesting that “α-cells exhibited weak connectivity in islets from both sexes” yet “were always in antiphase with the nearby insulin-positive β-cells.” At the top of the plateau potential all beta-cells are electrically connected and show rapid electrical signaling across the entire islet, which the authors observe in calcium traces (such as Fig. 3-1A traces). Thus, have the authors looked at the top of the plateau potential to see if the asynchronous patterns of single islet alpha-cell Ca2+ activities show greater synchronization?

>We appreciate the reviewer’s insightful comment. We have now discussed the differences between our study and Ren et al. on page 21. For Figure 2D-1 to D3, the frequency of α-cell activity at high glucose was sufficiently low to reveal the antiphase relationship between α- and β-cells. Furthermore, Supplementary Figures 2B and 2C demonstrate negative correlations between nearby α-β cell pairs, and the islet-wide correlation matrix between α- and β-cells further supports this antiphase relationship. Therefore, we think that an additional zoomed-in view of these activities would not provide additional meaningful information, hence is not provided. We have indeed examined the potential synchronization of α-cell activity at higher glucose concentrations, during which β-cell activity becomes synchronized. As shown in Figure 2C, only approximately 15% of GCaMP-expressing α-cells were active at high glucose, and these cells were sporadically distributed along the islet periphery. Furthermore, our network analysis (Supplementary Figure 1A) revealed limited correlation among these α-cells, indicating minimal synchronization.

The authors show an increase in islet cell activity following treatment with a variety of receptor inhibitors. In Figure 3-1 C-e the red representative alpha cell traces show a clear reduction of activity in response to elevated glucose levels. However, the hexbin plots of Ca2+ transient distributions (3-1 C-g) and the raster plots of Ca2+ transients (3-1 C-i) show that the glucose-dependence of alpha cell calcium responses is lost. An area under the curve measurement may allow for a clearer analysis of the changes of calcium. Or the representative traces are not truly representative. The same holds true for some of the beta-cell graphs. For example, Figure 3-1 B-f and 3-2 B-f shows blue traces that show a clear increase in calcium in response to elevations in glucose, although with some baseline drift. It seems like the changes in calcium in low glucose are mostly drift or movement and not typical of a normal beta cell calcium response. What is considered an amplitude of response that can be measured? Based on the representative beta cell traces there is some noise in the recordings, and it will be important to include some supplementary figures or methods indicating what was considered noise, drift, or movement versus a true beta calcium response. Some of the beta and delta cell responses clearly show movement such as in Fig. 6A delta cell ROI136 and 128 at the lowest glucose and similarly beta cell ROI124 and 200. What is considered movement or imaging artifact in the analysis could be more clearly defined.

> We thank the reviewer for these detailed comments. While the representative Ca^{2+} traces show an apparent reduction in amplitude, these activities are still counted as events as they exceed the threshold of z-score > 3. Accordingly, they are still reflected in the hexbin and raster plots as dots or vertical segments, which are inherently insensitive to the amplitude of Ca^{2+} events. The representative traces were chosen for clarity, but all ROIs were included in the analysis, so the plots accurately reflect the overall trends in the dataset.

Regarding the use of area under the curve (AUC) measurements, we refer the reviewer to our response to Reviewer 2, Question 2, which explains the rationale for using Ca^{2+} event frequency as a proxy for glucagon secretion rather than AUC.

Concerning drift, noise, or movement in the traces, the representative traces shown in the figures are unprocessed recordings, which we intended to show originality. For detailed analysis, these Ca^{2+} signals were further processed using analytical pipelines we previous developed; high-frequency events were detrended, distilled and then extracted, with events defined as signals exceeding a z-score of 3. Slow drifts or movement would have been detrended during processing and artifacts would be diluted across the large dataset. Only events meeting this threshold were considered true Ca^{2+} responses. Therefore, these factors do not affect the conclusions of the study. We refer

the reviewer to our previous published work for more details on the analytical process of the Ca^{2+} traces in islets in pancreas slices if required (PMID: 36449570).

Minor:
The following sentence might have a mistake,” These findings revealed heterogeneity in α-cell Ca2+ dynamics, which is shaped both by glucose but also by insulin and glucagon secreted from neighboring β-, and δ-cells.” It seems like the second glucagon in this sentence should be somatostatin.

> We are grateful to the reviewer for carefully identifying this oversight. We regret the editing error in the original manuscript and have corrected it in the revised version.

Reviewers' comments:

Reviewer #1 (Remarks to the Author):

I am happy with authors' revision of the manuscript
>We thank the reviewer for the positive evaluation of our work.

Reviewer #2 (Remarks to the Author):

The manuscript by Huang et al was improved by the revision. However, it is still not easily digested despite some rearrangements to improve alignment between text and illustrations. All reviewers have drawn attention to the use of Ca2+ as a proxy for secretion or whether it is average Ca2+ or frequency of Ca2+ changes that is important. I would like to return to this issue, which I think may shed light on some very unexpected results in the manuscript.

Ca2+ is a trigger of secretion in all islet cells but the relationship between Ca2+ concentration and the rate of secretion is far from clear. Ca2+ is basal in β cells exposed to low glucose and secretion is not triggered. There is consequently little effect of amplifiers of insulin release in this situation. When the threshold for glucose-stimulated Ca2+ influx is exceeded, secretion is triggered, and now amplifiers have potent effects. The situation in the glucose-regulated α cells is different. The relative glucose effect on glucagon secretion is less prominent than on insulin release. In particular, glucose doesn't turn off glucagon secretion completely (PMID: 21824262, PMID: 17136393) which is consistent with many observations that some Ca2+ signalling remains in glucose-inhibited α cells. Moreover, in the K-ATP-channel-centred model for glucagon secretion, Ca2+ influx through certain channels is more important for secretion than the total influx (PMID: 22177710). Since Ca2+ signalling remains in glucose-inhibited α cells, secretion-amplifying factors become important, and it has even been suggested that Ca2+ is a permissive factor that allows cAMP to mediate glucose regulation of glucagon secretion (PMID: 30953108).

Against this background I would like to return to my previous concern about the right-shifted β cell glucose threshold after SSTRA. When describing this phenomenon (lines 278-280) it is emphasized that the decline in α-cell Ca^{2+} activities and onset of β-cell Ca^{2+} activities were both right-shifted towards higher glucose concentrations. Later in discussion (lines 426-428) SST signalling blockade is instead stated to prolong α-cell hyperactivity and delay activation of coordinated β-cell Ca^{2+} oscillations. If the authors consider involvement of a temporal aspect, that need to be clarified. Otherwise, I suggest that "delay" is used with caution. Referring to the published data about antiphase Ca^{2+} oscillations between α- and β-cells (ref 40) the authors argue that "It is therefore intuitive that prolonged α-cell activity delays the onset of coordinated β-cell oscillation during the incremental glucose ramp observed" (433-435). Well, it might be superficially intuitive when considering the antiphase Ca2+ oscillations alone and disregarding that this antiphase relationship was observed in the absence of SSTRA. However, if the α cell Ca2+ oscillations reflect glucagon secretion, as assumed, it is instead counterintuitive that inhibited release of this potent amplifier of insulin secretion

coincides with activation the of insulin release. It seems much more reasonable that prevention of SST signalling should lower the glucose threshold for insulin secretion as reported in ref 63.

In their reply to my previous concern about the unexpected SSTRA effect on the β cell glucose threshold the authors emphasize that the α cells, rather than the β cells, are the focus of the current study. The authors nevertheless emphasize the α β cell connection with parallel but opposite right-shifts Ca2+ signalling but do not suggest any mechanism. Indeed, also I find it natural to focus on α β cell interactions since the effect is induced by SSTRA and persists when the insulin receptors are blocked. In any case this peculiar effect, which questions the use of α cell Ca2+ as proxy for glucagon secretion needs to be properly discussed.

>We thank the reviewer for the detailed discussion and for outlining the physiological rationale suggesting that SSTRA might lower the glucose threshold for β-cell activation, as reported in ref. 63.

We agree with the reviewer that cytosolic Ca^{2+} is not a perfect proxy for hormone secretion, and this limitation is now further extended in line 479-482 of the revised manuscript. We would also like to note that the insulinotropic action of glucagon is widely considered **glucose-dependent**, becoming prominent primarily under elevated glucose conditions while exerting minimal effects at low glucose levels (PMID: 31335319; 30380405). In this context, increased α-cell Ca^{2+} activity, reflecting enhanced glucagon secretion presumably, would not necessarily be expected to lower the glucose threshold for β-cell activation. Rather, glucagon is generally thought to amplify insulin secretion once β-cells are already activated by glucose. Thus, enhanced α-cell activity during the ramp protocol does not necessarily predict a leftward shift in β-cell Ca^{2+} activation by glucose but may instead influence β-cell responses once glucose-dependent activation has already occurred.

We further note that our observed shift in β-cell Ca^{2+} activity under SSTRA treatment does not necessarily indicate reduced insulin secretion. Although the onset of oscillations is shifted, insulin release could still be substantially amplified once Ca^{2+} oscillations are established at higher glucose concentrations. Our glucose ramp protocol unfortunately did not allow assessment of β-cell functional responses at glucose concentrations above 10.8mM, as the protocol was originally designed to prioritize detailed assessment of α-cell Ca^{2+} activity. Nevertheless, the delay should be interpreted as a shift in β-cell activation rather than definitive evidence of reduced secretory output. We have acknowledged in the manuscript that direct measurement of hormone secretion will be required to resolve these questions; however, quantifying hormone release from single cells - which would be the most relevant assay in our study given our focus on cell-cell interactions and heterogeneity - in islets in intact pancreas slices presently remains technically challenging (as we discuss carefully in the Discussion section of the manuscript), especially if the object is to measure the release of the three most relevant islet hormones simultaneously! We would note that our present studies were not, therefore, performed with simultaneous measurements of “bulk” hormone secretion and it would be infeasible now to repeat all of the studies with this objective.

We would like to reinforce that we consistently observed a rightward shift in β-cell Ca^{2+} activation when α-cell hyperactivity was induced, either by SST receptor antagonism in female islets or insulin receptor antagonism in male pancreas slices. This consistency indicates that the phenomenon is not specific to SSTRA but likely reflects altered islet network dynamics. These findings suggest that α-β network interactions and antiphase oscillatory dynamics may exert dominant control beyond simplified threshold models of glucose-stimulated insulin secretion. Supporting this interpretation, several recent studies indicate that α-cell can strongly influence the dynamic Ca^{2+} behavior of neighboring β-cells (PMID: 35764654; **doi:** https://doi.org/10.1101/2024.08.21.608986/ **doi:** https://doi.org/10.1101/2025.08.09.669458).

Lastly, we would also like to emphasize that in ref. 63 (Figure 7), α-cell activity was not examined concurrently with β-cell activity in their isolated islet model, in which δ-cells were ablated and β-cells appeared partially labeled (Figure 7C andD). This limits insight into global islet network behavior.

More importantly, in Figure 3M–N of ref. 63 (also shown on the right), glucose-stimulated insulin secretion in δ-cell-ablated Sst-Cre × DTR islets (green bars) was not siginificantly increased compared with controls (blue bars) at **3, 7, or 11 mM glucose,** and was enhanced only at supraphysiological glucose (16.8 mM). Thus, these insulin secretion data do not substantiate enhanced β-cell activation at lower glucose concentrations as shown in their paper and are in fact more consistent with the rightward-shifted β-cell functional responsiveness observed in our pancreas slice experiments.

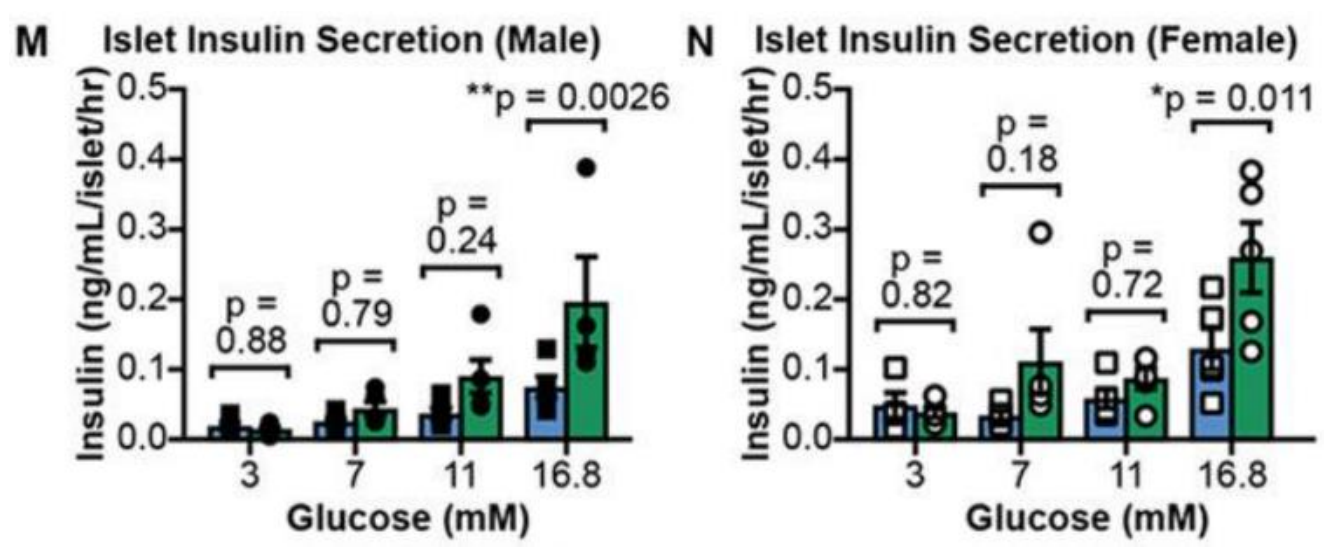

We have expanded the Discussion to address the above points (lines 437-447) and in response to reviewer's request, we revised the text wherever "delay" was used.

Reviewer #3 (Remarks to the Author):

The manuscript by Ya-Chi Huang et al. utilizes pancreatic slice calcium imaging to examine islet cell responses from male and female mice to secretagogues and in relation to neighboring cells. The data does improve the understanding of islet cell calcium handling. While the authors addressed all concerns some of these comments were a bit concerning.

For example two reviewers asked for area under the curve (AUC) analysis and the authors indicate that, "Ca^{2+} oscillation frequency is, in our view, a more reliable proxy for hormone secretion than the total signal magnitude." The authors also indicate that, "Furthermore, oscillations in intracellular Ca^{2+} concentration (leading to repetitive Ca2+ increases, and in contrast to sustained Ca2+ elevations) in islet endocrine cells are the key determinant of the probability (and thus the rate) of granule exocytosis." In response to this:

Frequency is important, but amplitude does have significant impacts on secretion (e.g., KCl stimulation causes greater secretion). I am a bit surprised by the authors argument against AUC. Especially when considering that line-scanning confocal imaging would never allow the primary calcium signal (the millisecond based action potential-induced calcium spike) to be detected. Most electrophysiology is recorded at 20 Khz or faster, which is 3200 times the speed of 6.25 hz. At 6.25 hz an image is taken every 120 milliseconds. As an action potential waveform from a beta-cell is typically ~10 milliseconds in duration, with 6.25 hz imaging speed only one image during an action potential would be possible and only if it was taken at the right time (the image could hit the peak of an action potential or a point between action potentials or a point on the upstroke or repolarization of an action potential). More importantly, alpha-cells fire sodium channel dependent action potentials that are even faster <5 milliseconds in duration. To see a complete action potential calcium response, you would need to image the calcium during the action potential upstroke as well as repolarizing phase, which the line-scanning confocal approach cannot. Therefore, this statement about calcium frequency is a bit misleading, because you are missing most of the action potentials that actually stimulate secretion. Importantly, the frequency plots of this manuscript do not indicate the primary calcium kinetics of an islet cell that occur during an action potential. Thus, the frequency argument against using AUC is flawed with this experimental approach.

> We thank the reviewer for this thoughtful and technically detailed comment regarding the interpretation of Ca^{2+} oscillation frequency and the limitations imposed by imaging speed. We would like to respectively clarify that we are not opposing a role for changes in Ca^{2+} AUC measurements and recognize that Ca^{2+} oscillation amplitude can influence secretion, particularly under strong depolarizing conditions, such as KCl stimulation.

We recognize that in our experimental configuration (6.25 Hz confocal imaging), the recorded fluorescence signal reflects integrated cytosolic Ca^{2+} dynamics rather than millisecond action potential-associated Ca^{2+} microdomains. This approach does not directly resolve individual electrical spikes (5-10 ms in β-cells and faster still in α-cells) but instead integrates them temporally into mesoscopic oscillatory events via Ca^{2+} buffering and indicator kinetics. Under these conditions, the area under the curve (AUC) is influenced not only by event amplitude, but also by baseline shifts, slow tonic elevations, decay kinetics, indicator loading variability and photobleaching. This confounds several independent parameters, making AUC less reliable. In contrast, oscillation frequency quantifies the number of discrete activation episodes per unit time and more directly reflects the repetitive Ca^{2+} elevations associated with granule fusion probability in endocrine cells. We therefore think that reporting frequency provides a more robust and physiologically interpretable metric within the temporal resolution limits of our imaging approach.

Below, we attached the analysis of area under the curve (AUC) of the Ca^{2+} signal reported in Figure 4 of the manuscript. The overall trends observed in the AUC analysis were consistent with those obtained from the oscillation frequency analysis. However, in this analysis, the strength of the difference between groups is reduced due to the limitations of this approach as discussed above. In the revised manuscript, we retained frequency-based quantification as the primary metric in the main figures and inserted the AUC analysis as Supplementary Figure 6.

α-cells

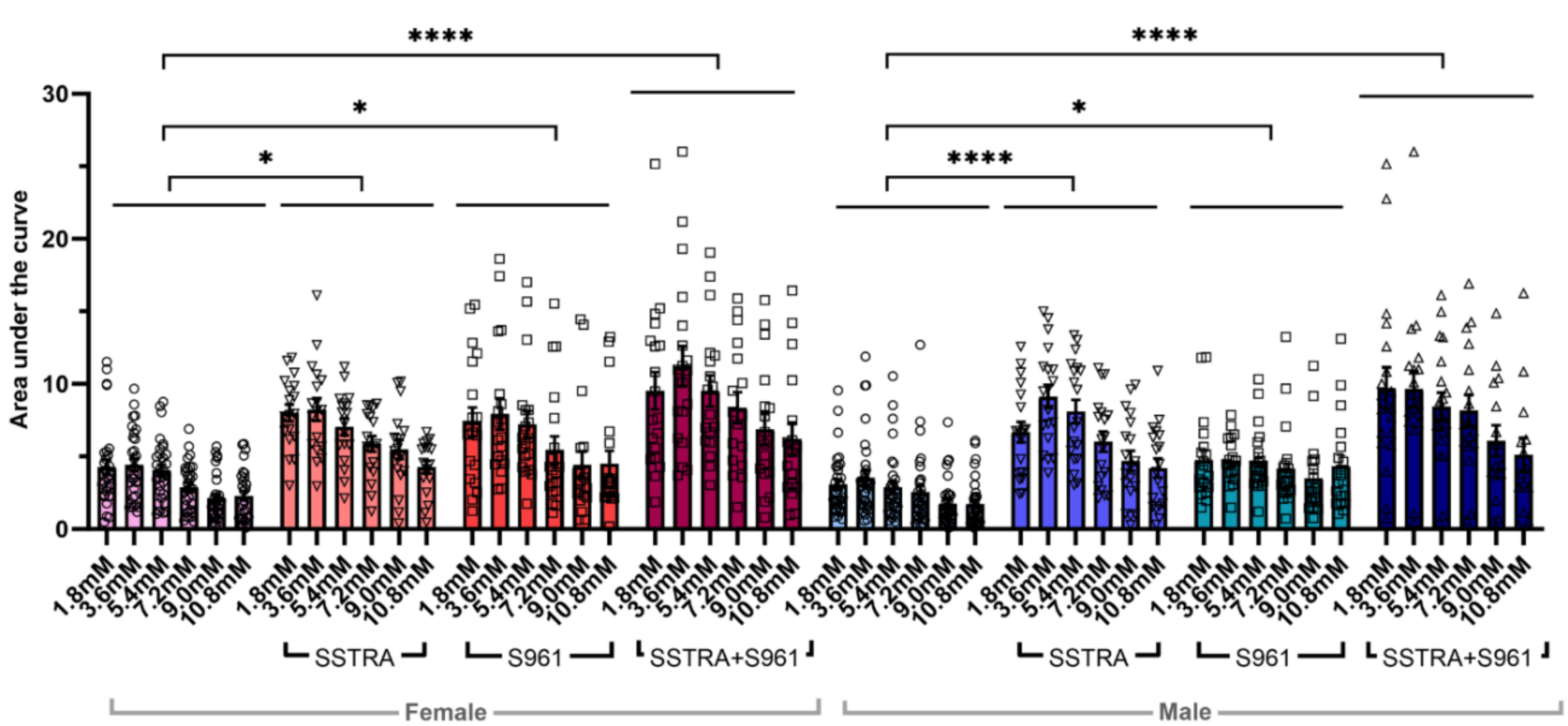

β-cells

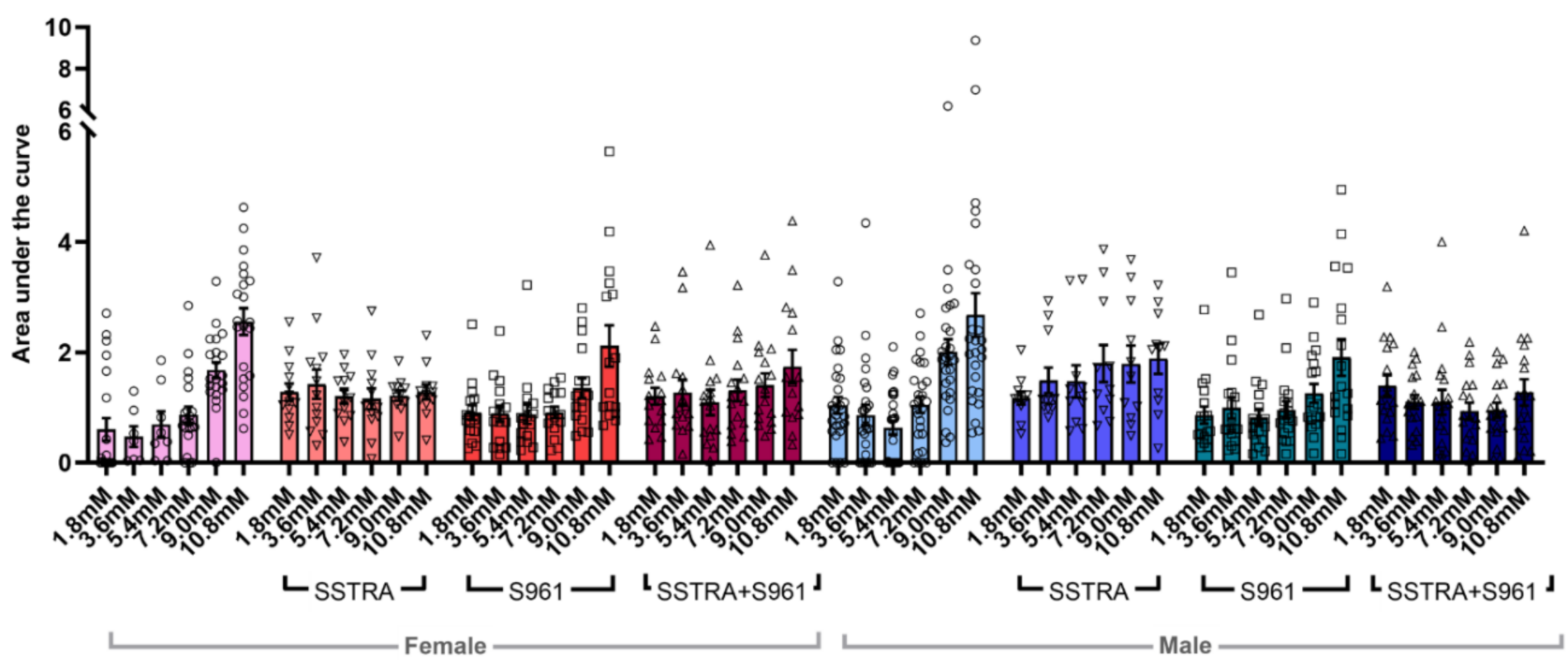

Supplement: Supplementary file 1 [file 42003_2026_10163_MOESM1_ESM.pdf]
