# Supplementary material for: Pancreatic α-cells are functionally heterogeneous and sex-dependently regulated by neighboring endocrine cells
Source: Commun Biol. 2026 Jun 23;9:1324. doi: 10.1038/s42003-026-10163-6 (PMC13655181; doi:10.1038/s42003-026-10163-6)
Supplement: Supplementary file 2 [file 42003_2026_10163_MOESM2_ESM.pdf]

Supplementary Figure 1

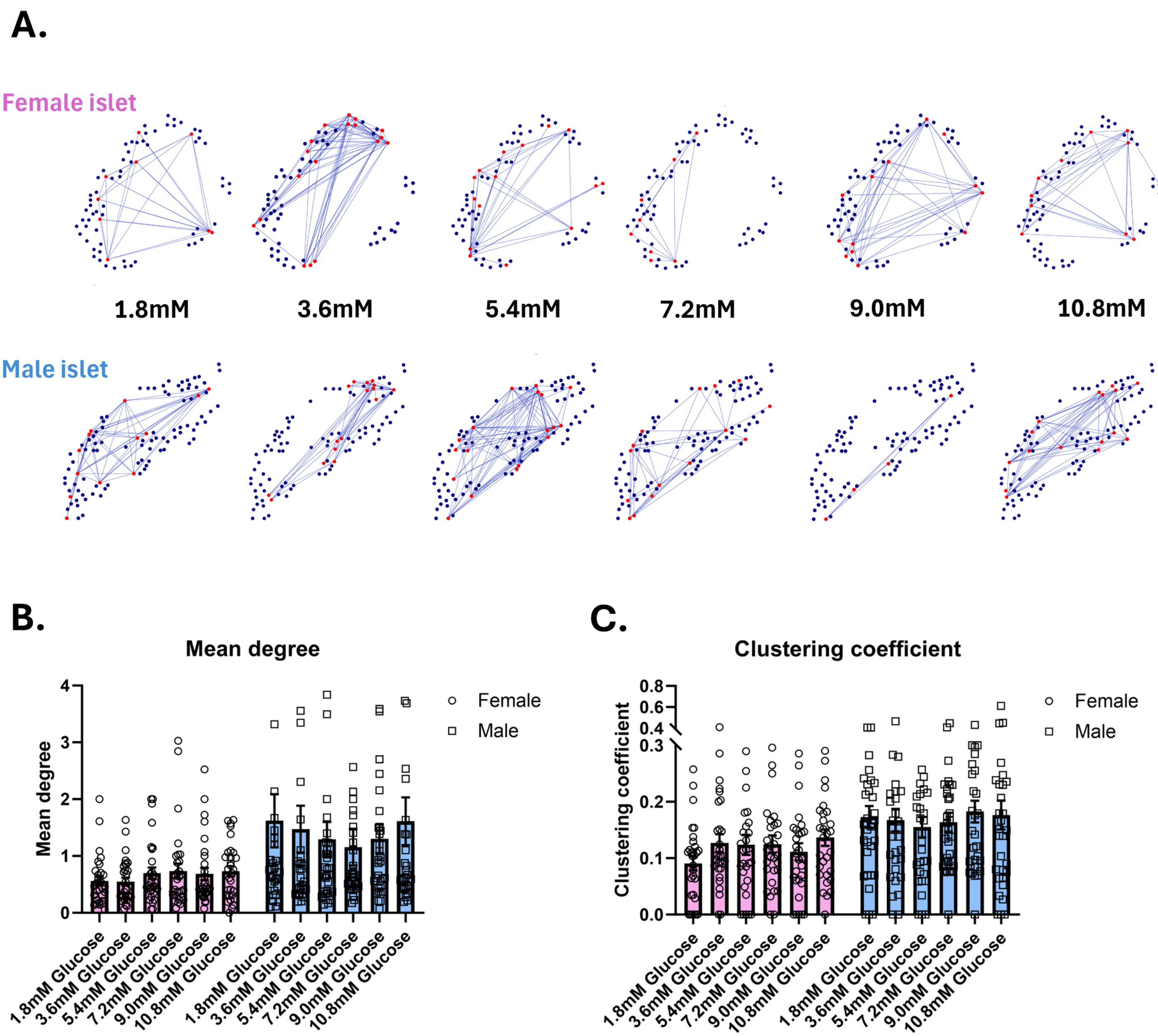

Supplementary Figure 2

A

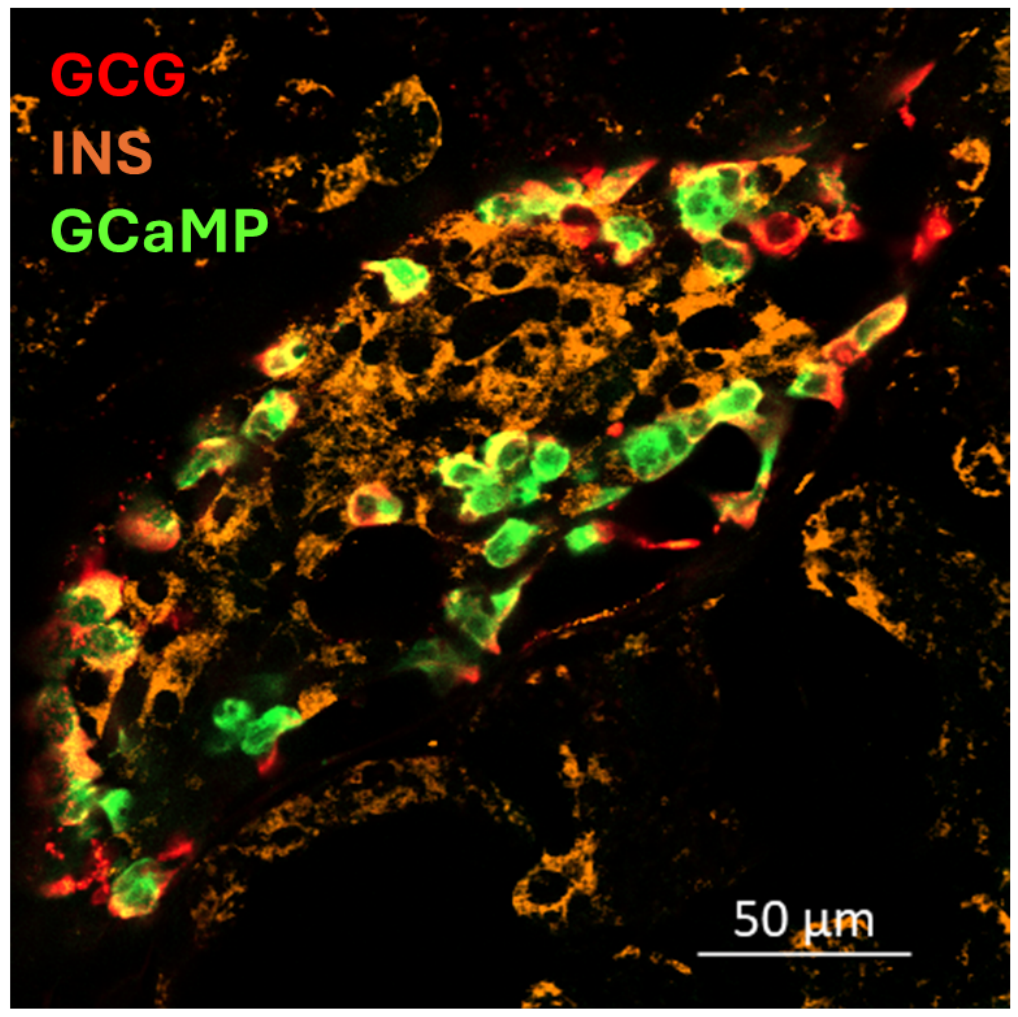

B Negative correlations between nearby α- vs. β-cell pairs

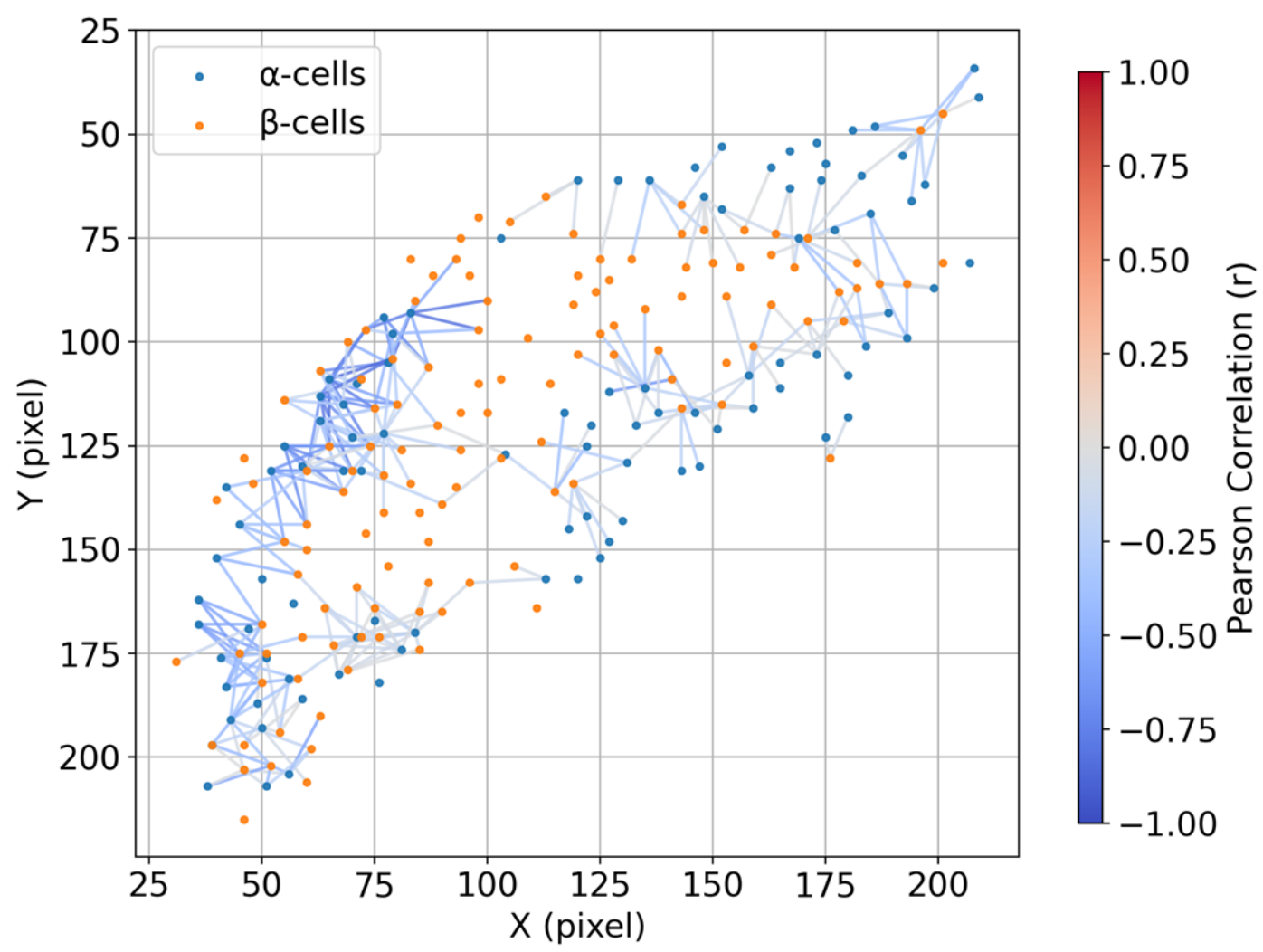

C Pairwise Correlation Matrix Between α- and β-cells

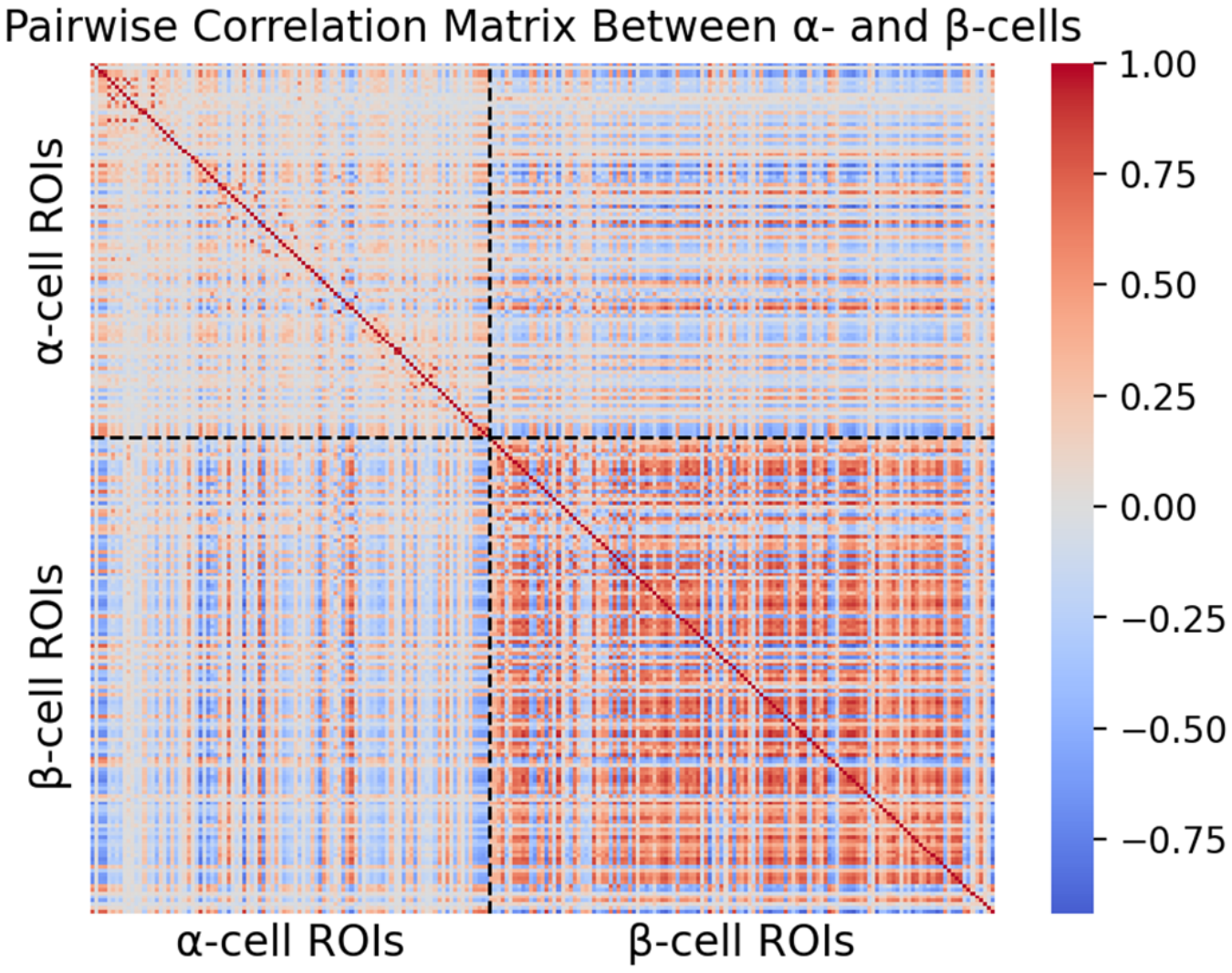

Supplementary Figure 3

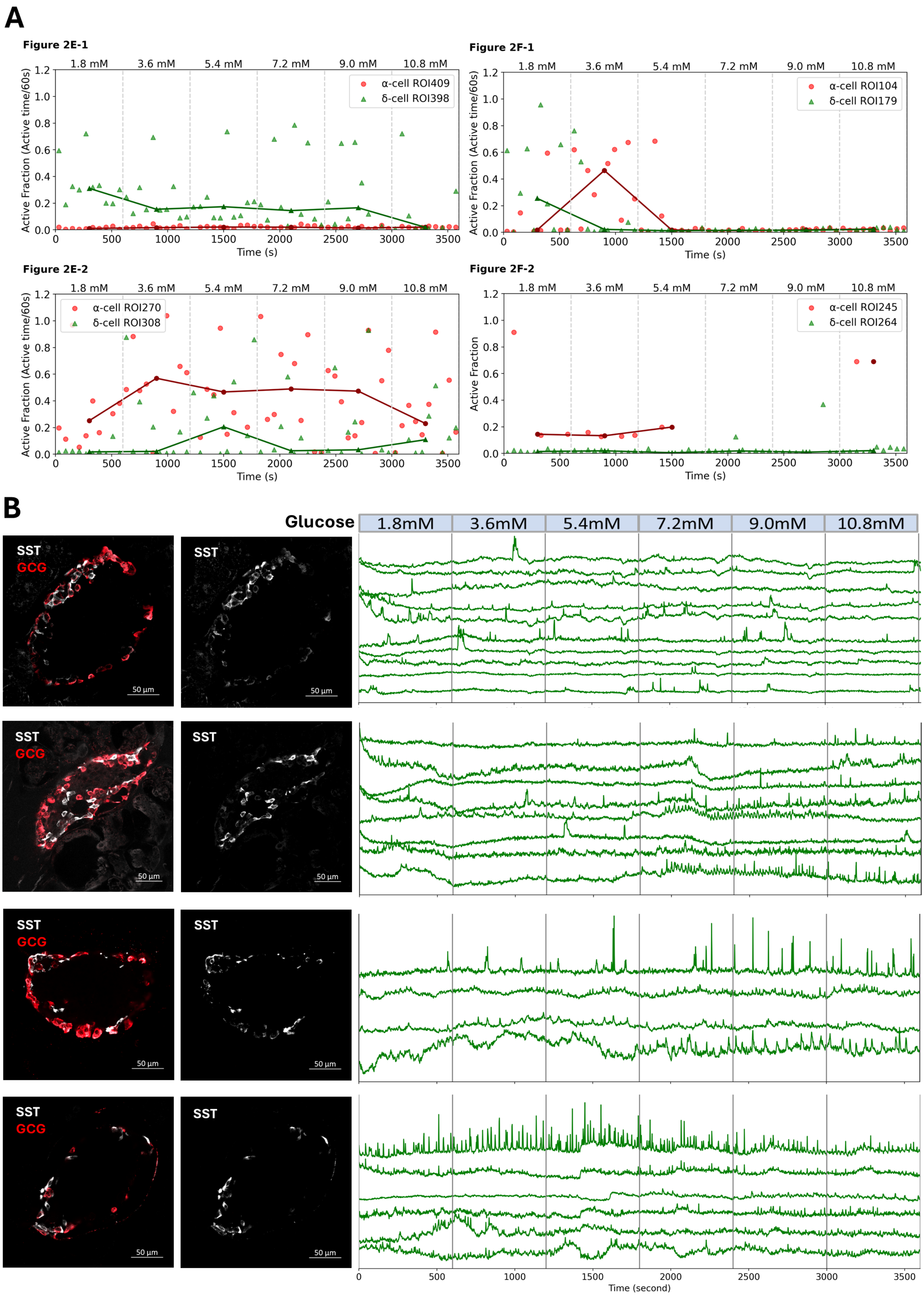

Supplementary Figure 4

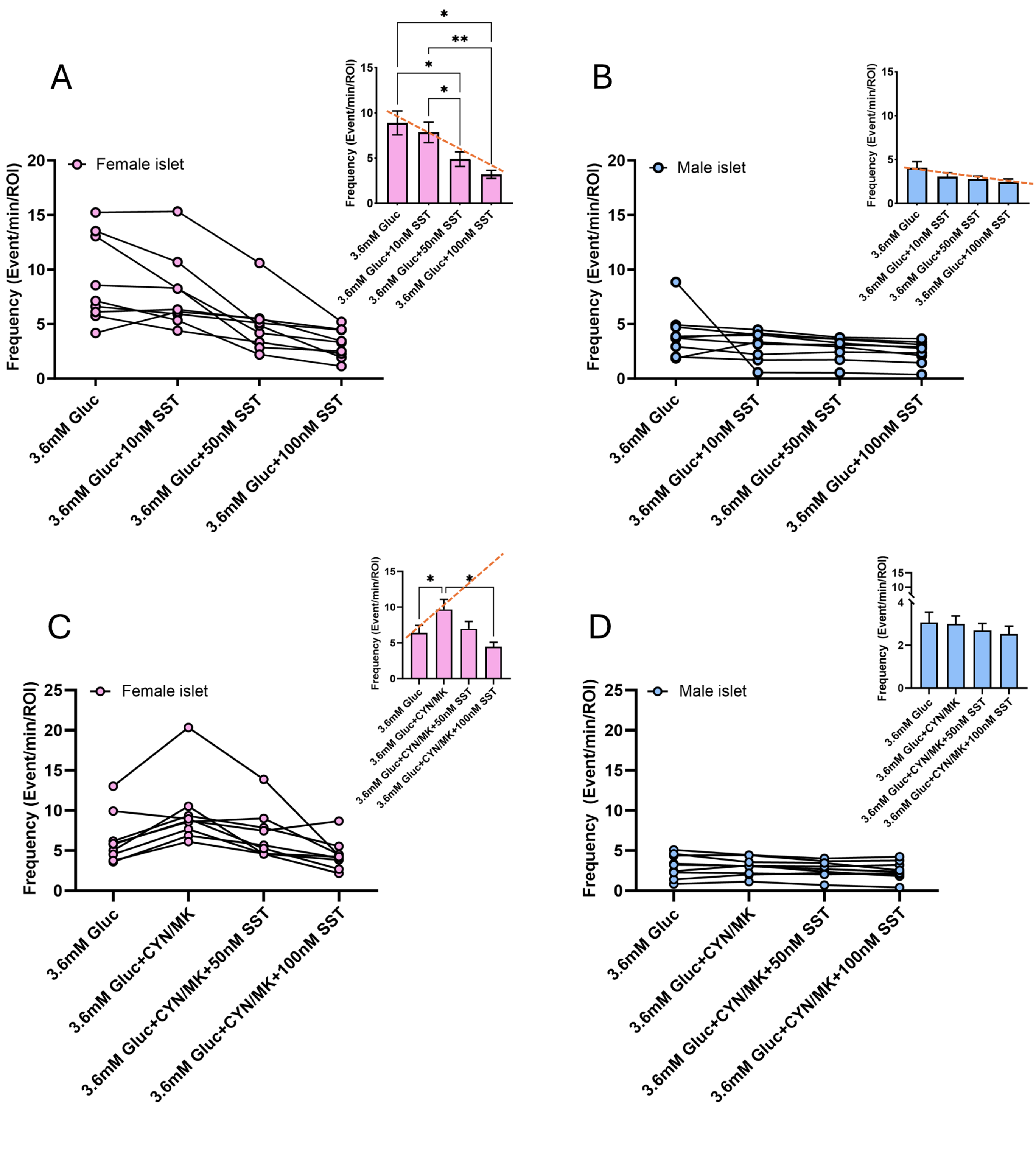

Supplementary Figure 5

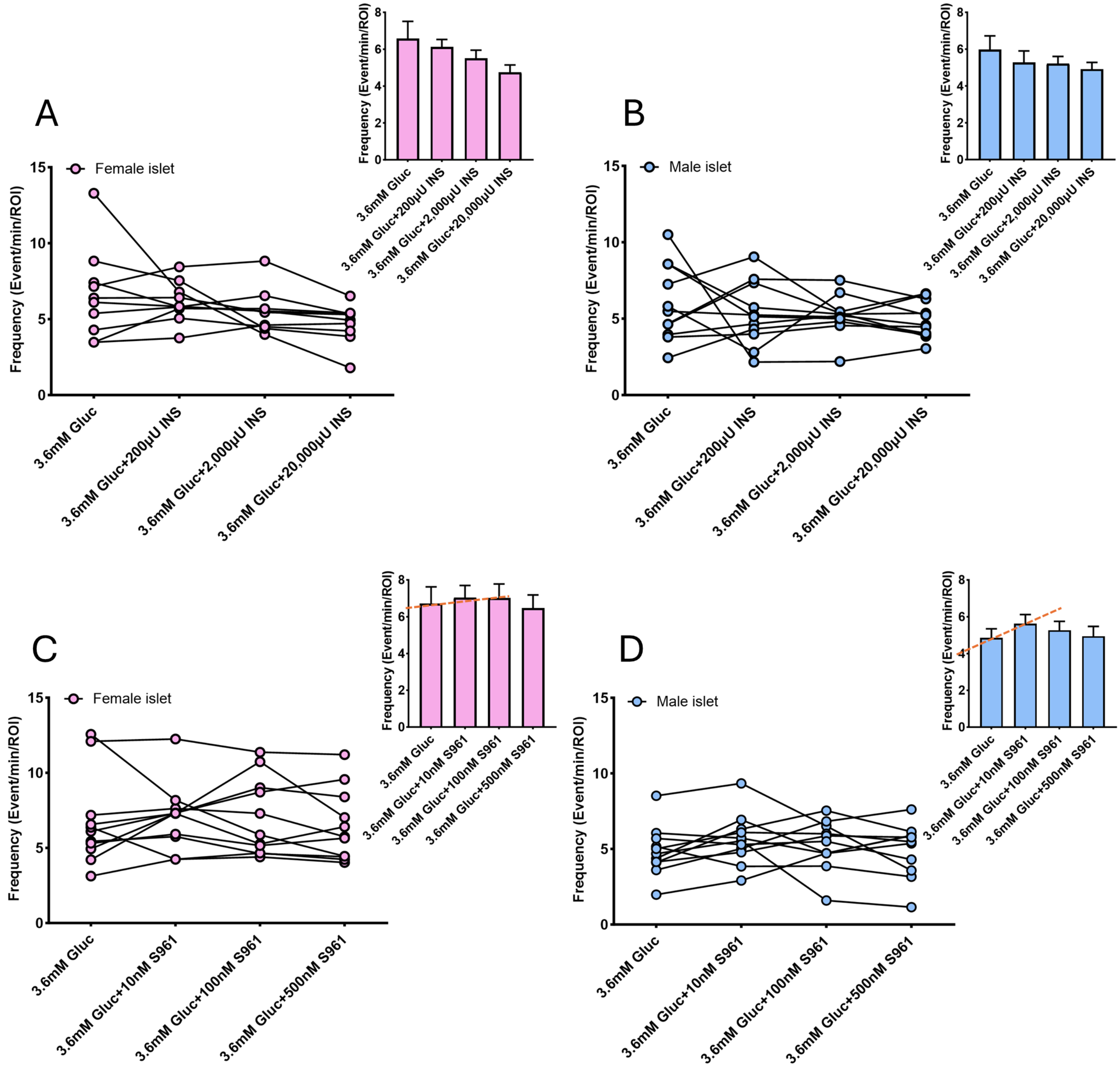

Supplementary Figure 6

α-cells

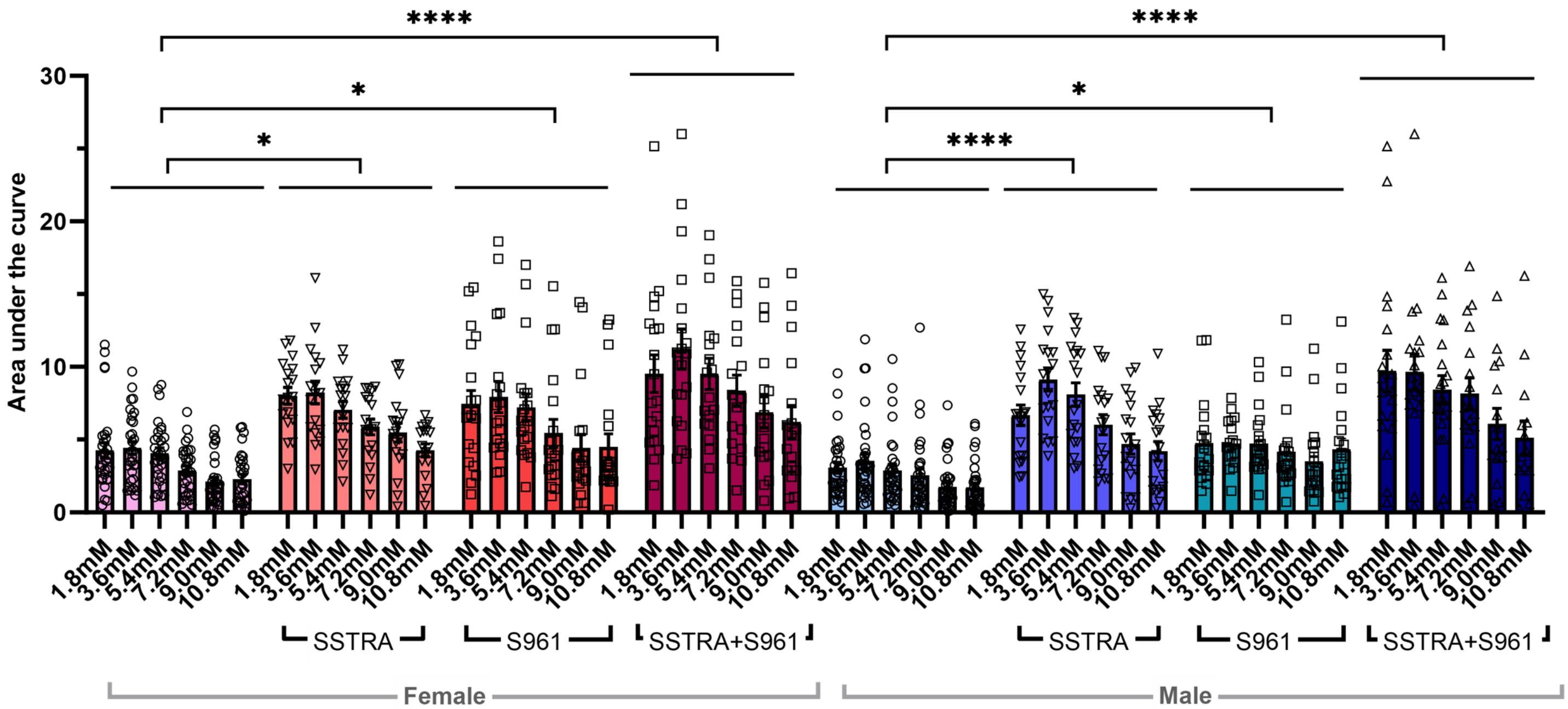

β-cells

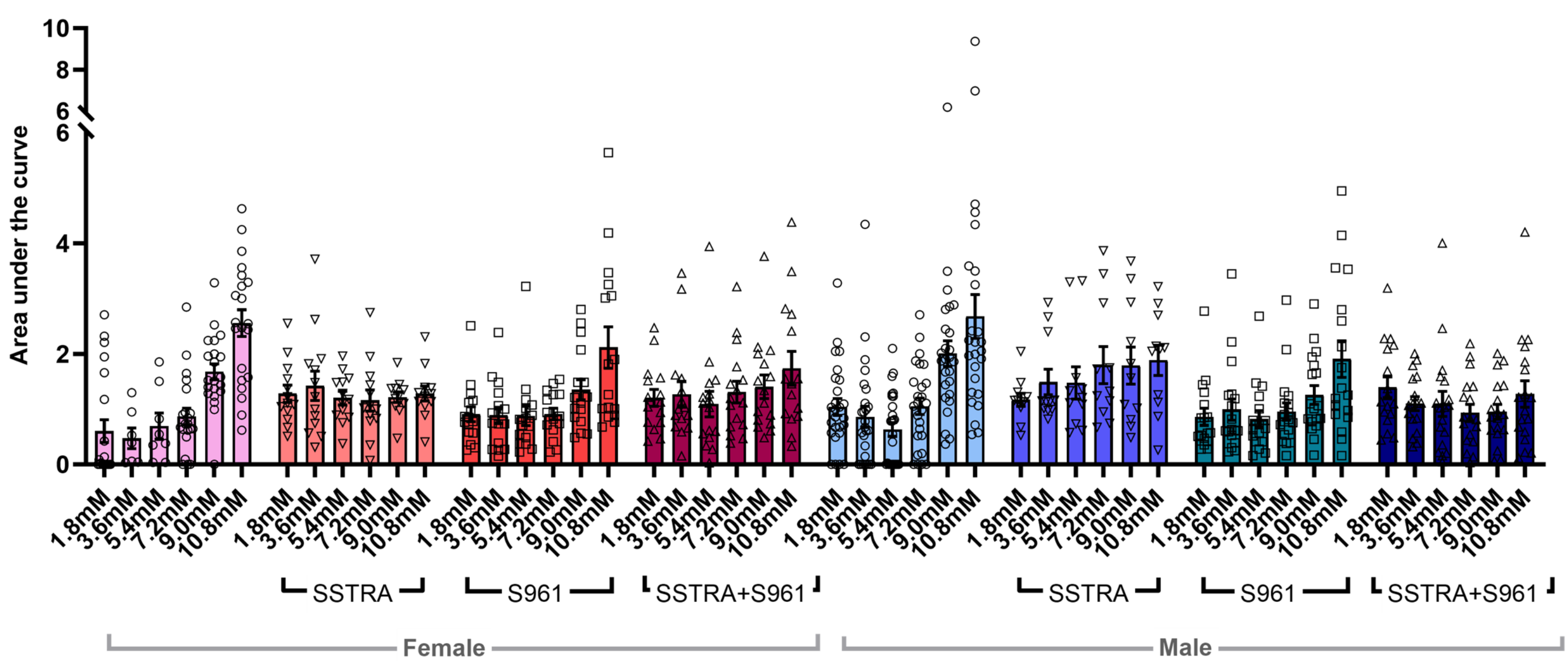

Supplementary Figure 7

A)

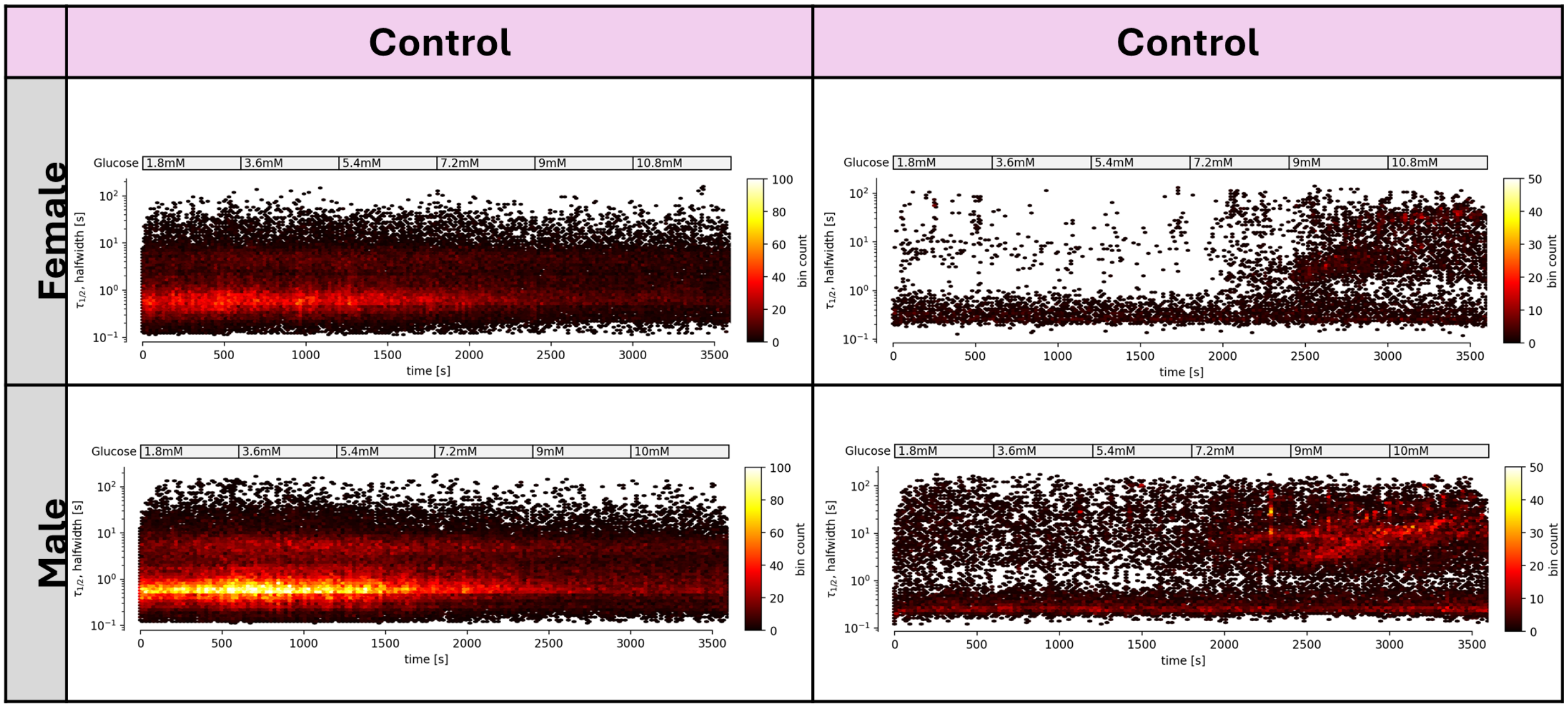

B)

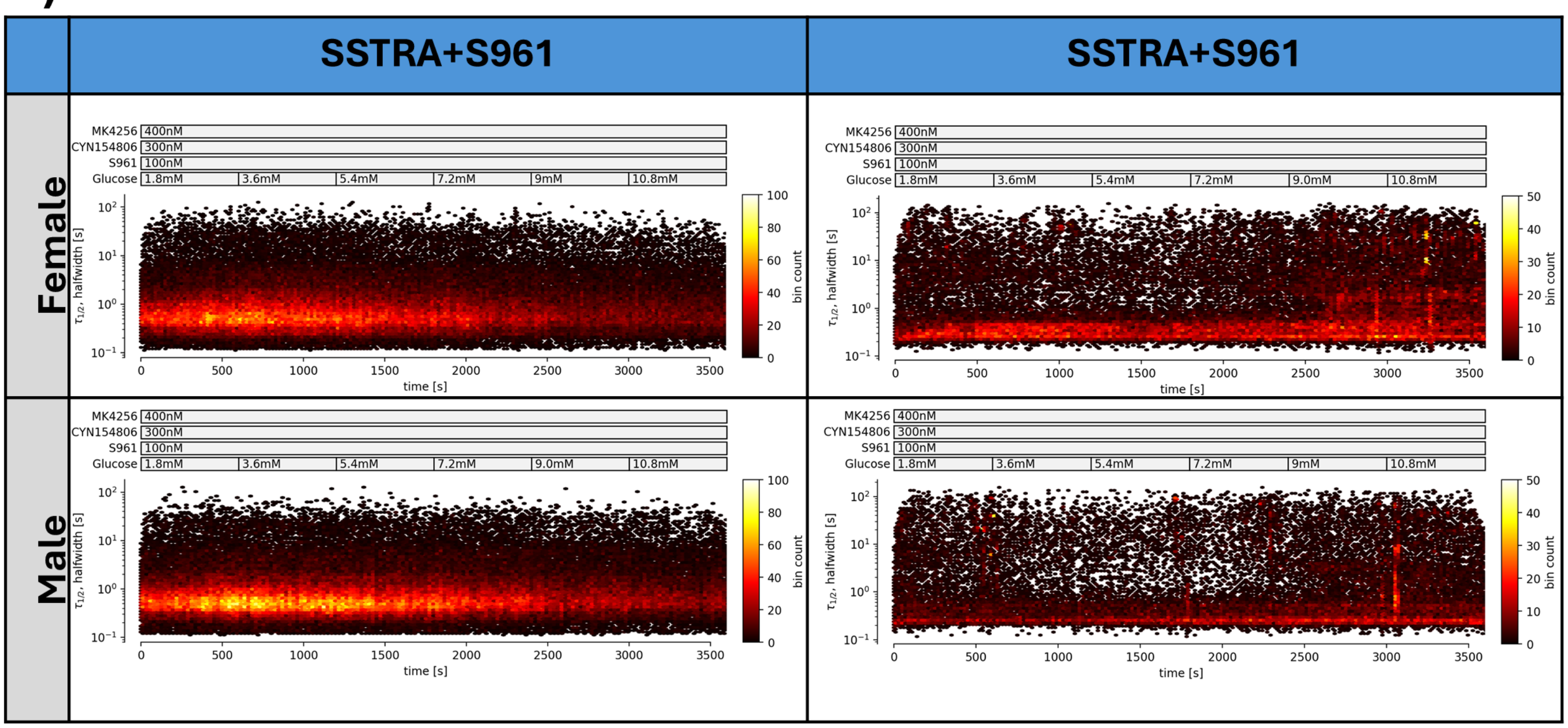

C)

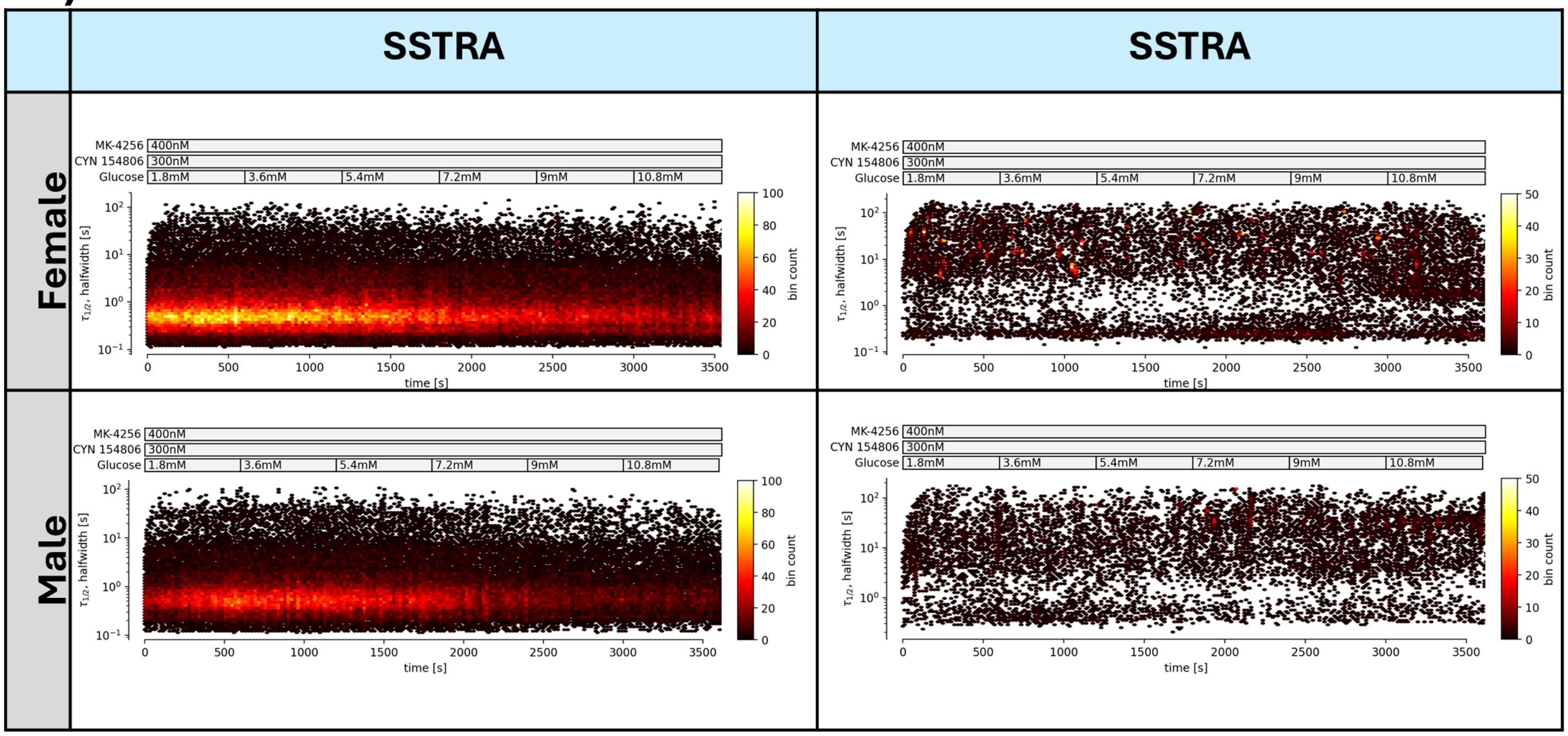

SSTRA
SSTRA
Female
Male
MK-4256 400nM
CYN 154806 300nM
Glucose 1.8mM 3.6mM 5.4mM 7.2mM 9mM 10.8mM
τ1/2, halfwidth [s]
time [s]
bin count

D)

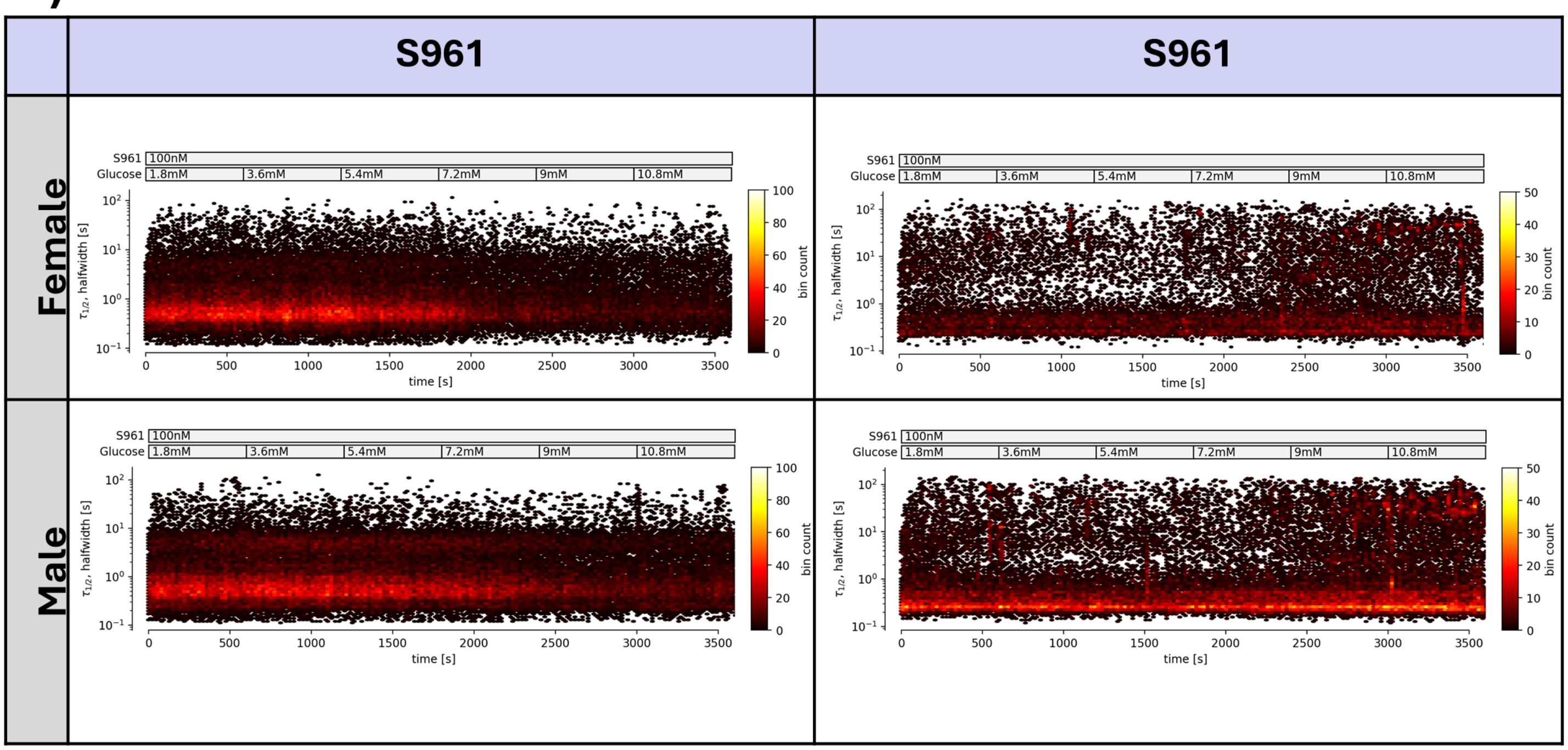

S961
S961
Female
Male
S961 100nM
Glucose 1.8mM 3.6mM 5.4mM 7.2mM 9mM 10.8mM
τ1/2, halfwidth [s]
time [s]
bin count

Supplementary Figure Legends

Supplementary Figure 1: Functional network analysis of α-cell Ca^{2+} dynamics

A. Representative α-cell networks in female (top) and male (bottom) islets across six glucose concentrations corresponding those in the glucose ramp. Each dot (known as nodes) represents an α-cell ROI. Connected nodes are shown in red, isolated nodes in navy. Note the weak connectivity across all networks with mostly isolated nodes. Quantification of **B.** Mean degree (average links per node) and **C.** clustering coefficient (local clustering tendency) across the six-glucose concentration over n = 30 islets from 5 females (85 ± 7.81 days) and 32 islets from 6 males (76 ± 9.31 days). Error bars represent mean ± standard error of the mean (SEM). All animal age were provided as mean ± standard deviation (SD).

Supplementary Figure 2: Spatial mapping reveals anticorrelated Ca^{2+} activity between juxtaposed α- and β-Cells in mouse islets

A. A representative islet underwent high-speed calcium imaging followed by post-hoc immunostaining. The image shows GCaMP expression in glucagon-positive cells and their spatial relationship to insulin-positive areas. The corresponding glucagon- and insulin-positive regions are spatially mapped in **B.** Scatter plots display α-cells (blue) and β-cells (orange) positioned according to their x-y coordinates within image **A**. Negatively correlated α- and β-cell ROIs are spatially mapped. Lines connecting α-β cell pairs represent Pearson correlation coefficients (r) of Ca^{2+} activity, color-coded by strength according to the scale bar on the right. **C.** This heatmap visualizes Pearson correlation coefficients computed between Ca^{2+} activity traces of α-cell and β-cell ROIs within islet shown in **A.** The matrix is divided into four blocks by dashed lines: top-left for α-α correlations, bottom-right for β-β correlations, and the off-diagonal blocks for α-β and β-α correlations. Color intensity and hue represent correlation

strength and direction, with red indicating positive and blue indicating negative correlations, centered at zero.

Supplementary Figure 3: Quantification of active fraction of adjacent α- and δ-cells and heterogeneous Ca^{2+} activity in GluCre:GCaMP6f mouse

A. Each of the four panels shows the active fraction of individual cells, corresponding to the quantification of Ca^{2+} activity trances of α- and δ-cell pairs in the main Figures 2E-1, 2F-2, 2F-1, and F-2 (also labeled at the top left of each panel). Green triangles (for δ-cells) and red circles (for α-cells) indicate individual measurements of active fraction per 60 seconds. Active fraction is defined as the proportion of each 60-second interval during which Ca^{2+} signals were elevated. The overlayed dark green triangles and dark red circles highlight the median active fraction across each glucose concentration range. Cell types and ROI numbers are indicated in the legends. Note the inverse relationship in the Ca^{2+} activity between α- and δ- cells in the left panels, and the temporally inverse relationship in the right panels.

B. Left panels show islets from four different mice, immune-stained for glucagon (GCG) and somatostatin (SST) to identify α- and δ-cells, respectively. Right panels display the corresponding Ca^{2+} activity traces recorded from δ- cells within the islets shown on the left. Note the heterogeneous nature of Ca^{2+} dynamics among individual δ-cells and across different islets.

Supplementary Figure 4: Somatostatin suppresses α-cell Ca^{2+} dynamics via SSTR-mediated inhibition in female mice.

Somatostatin (SST; 10, 50, 100nM, 10 minutes for each concentration), when applied to female **(A)** and male **(B)** GluCre:GCaMP6f mouse pancreas slices at 3.6mM glucose condition, reduced the frequency of Ca^{2+} activities in α-cells in a dose dependent manner. Each circle (pink: female;

blue: male) represents the mean α-cell Ca^{2+} activity frequency of a single islet. Insets show quantification of mean ± SEM from individual islets represented in the main plots. Note the steeper slope (orange dashed line) in **A-inset** than in **B-inset,** indicating greater somatostatin sensitivity in female α-cells. n = 9 islets from 3 female mice (101 ± 6.2 days) and 9 islets from 3 male mice (113.3 ± 1.5 days).

Somatostatin receptor antagonists MK4256 and CYN154806 (SSTRA; 400 and 300 nM, respectively, 10 minutes for each concentration) increased α-cell Ca^{2+} activity in females (**C**; n = 10 islets from 3 mice, 101 ± 6.2 days), but only in few male islets (**D**; n = 9 islets from 3 mice, 113.3 ± 1.5 days). In female islets, SSTRA-induced potentiation was reversed by 50 or 100nM SST, suggesting binding competition between SST and SSTRA and confirming a direct regulatory effect of somatostatin on α-cell excitability. All animal age were provided as mean ± SD.

Mixed-effects model analysis was performed, followed by Bonferroni's post-hoc test. Islets are considered as the primary unit of analysis while accounting for treatment as fixed-effects and mouse as random intercept. Significance levels are indicated as: $p < 0.05$ (*), $p < 0.01$ (**). Effect size indicated as difference in means ± SEM. **A inset**: 3.6mM Gluc vs. 3.6mM Gluc+50nM SST showed a difference of -4.01 ± 0.83 (95% CI: -6.61 to -1.40); 3.6mM Gluc vs. 3.6mM Gluc+100nM SST showed a difference of -5.71 ± 0.83 (95% CI: -8.31 to -3.10); 3.6mM Gluc+10nM SST vs. 3.6mM Gluc+50nM SST showed a difference of -2.95 ± 0.83 (95% CI: -5.65 to -0.35); 3.6mM Gluc+10nM SST vs. 3.6mM Gluc+100nM SST showed a difference of -4.65 ± 0.83 (95% CI: -7.26 to -2.05); **C inset**: 3.6mM Gluc vs. 3.6mM Gluc+CYN/MK showed a difference of 3.27 ± 0.98 (95% CI: 0.22 to 6.32); 3.6mM Gluc+CYN/MK vs. 3.6mM Gluc vs.

3.6mM Gluc+CYN/MK+100nM SST showed a difference of -5.22 ± 0.98 (95% CI: -8.27 to -2.17). Error bars represent mean ± SEM.

Supplementary Figure 5: Effects of insulin and insulin receptor blockade on α-cell Ca^{2+} dynamics.

Insulin (INS; 200, 2,000, 20,000μU/mL) was applied to pancreas slices from female **(A)** and male **(B)** GluCre: GCaMP6f mouse at 3.6mM glucose condition, resulting in a downward trend in α-cell Ca^{2+} activity frequency **(A and B, insets)**. The insulin receptor antagonist S961 (10, 100, 500) elicited a mildly potentiated α-cell Ca^{2+} dynamics in both sexes **(C, females; and D, males)**. Each data point (pink circle: female; blue circle: male) represents the mean α-cell Ca^{2+} oscillation frequency for an individual islet. Insets display quantification (mean ± SEM) from islets shown in the main panels. INS treatment: n = 10 islets from 3 females (86 ± 36 days) and 11 islets from 3 males (93 ± 5.1 days). S961 treatment: n = 11 islets each from 3 female and 3 male mice (86 ± 36 and 93 ± 5.1 days, respectively). Statistical analysis was performed using mixed-effects model. All animal age were provided as mean ± SD. Error bars represent mean ± SEM.

Supplementary Figure 6: Area under the curve (AUC) of α- and β-cell Ca^{2+} activity across glucose concentrations and receptor antagonists.

AUC quantification of Ca^{2+} activity in α-cells (top) and β-cells (bottom) corresponding to the experiments shown in Figure 4. Experimental groups, mouse numbers, ages, sample sizes, color coding, and statistical analysis are identical to those described in Figure 4. Bars represent mean AUC ± SEM for female (pink, orange, red, dark red) and male (light blue, purple, green, navy) islets under glucose ramp alone, glucose ramp + SSTR antagonists (SSTRA: 400 nM MK4256 +

300 nM CYN154806), glucose ramp + insulin receptor antagonist (S961: 100 nM), or glucose ramp + SSTRA + S961. Within each glucose concentration, the AUC was summed per ROI, then normalized to duration of the glucose treatment. Each data point represents the mean AUC of α- or β-cell Ca^{2+} events per minute per islet. Statistical analysis was performed using the same mixed-effects model described in Figure 4. Note the overall trend is consistent with the Ca^{2+} oscillation frequency analysis (Figure 4). Significance levels are indicated as: $p < 0.05$ (*), $p < 0.01$ (**), $p < 0.001$ (***), and $p < 0.0001$ (****). Effect size indicated as difference in means ± SEM. **Top panel α-cells** Females: Compared with control, SSTRA showed a difference of 3.27 ± 1.29 (95% CI: 0.74 to 5.79); S961 showed a difference of 2.52 ± 1.28 (95% CI: 0.003 to 5.04); and the combined SSTRA+S961 treatment showed a difference of 5.53 ± 1.29 (95% CI: 3.00 to 8.05). Males: Compared with control, SSTRA showed a difference of 3.80 ± 0. 90 (95% CI: 2.05 to 5.56); S961 showed a difference of 1.75 ± 0.89 (95% CI: 0.001 to 3.50); and the combined SSTRA+S961 treatment showed a difference of 5.33 ± 0.91 (95% CI: 3. 54 to 7.12). Error bars represent mean ± SEM.

Supplementary Figure 7: Population-level Ca^{2+} activity dynamics visualized by hexbin event density plots.

Hexbin density plots illustrate the spatiotemporal distribution of Ca^{2+} events in α- and β-cells across all recorded islets. Panels **A-D** share a consistent layout: α-cells (left) and β-cells (right) from female (top row) and male (bottom row) GluCre:GCaMP6f mice were analyzed under the following conditions: (**A**) glucose ramp alone, (**B**) SSTR antagonists (SSTRA) + insulin receptor antagonist S961, (**C**) SSTRA alone, and (**D**) S961 alone. The x-axis indicates the time (in seconds) when Ca^{2+} events reached their peak; the y-axis indicates event duration at half-maximum amplitude (halfwidth, log-scaled seconds). Warmer colors denote higher event

density. These plots represent a global view of event timing and variability across the entire α- and β-cell populations.

A) Glucose ramp alone: α-cells: n = 30 islets from 5 female mice (85 ± 7.8 days) and 48 islets from 9 male mice (78.9 ± 10.6 days); β-cells: n = 21 islets from 5 female mice (85 ± 7.8 days) and 26 islets from 8 male mice (79.8 ± 8.8 days).

B) SSTRA + S961: α-cells: n = 19 islets from 3 female mice (88 ± 7.0 days) and 19 islets from 3 male mice (94.3 ± 6.4 days); β-cells: n = 16 islets from 3 female mice (88 ± 7.0 days) and 18 islets from 3 male mice (94.3 ± 6.4 days).

C) SSTRA alone: α-cells: n = 18 islets from 3 female mice (65.3 ± 5.5 days) and 18 islets from 3 male mice (77.3 ± 3.8 days); β-cells: n = 17 islets from 3 female mice (65.3 ± 5.5 days) and 11 islets from 3 male mice (77.3 ± 3.8 days).

D) S961 alone: α-cells: n = 19 islets from 3 female mice (84 ± 2.0 days) and 19 islets from 3 male mice (86.5 ± 10.1 days); β-cells: n = 16 islets from 3 female mice (84 ± 2.0 days) and 19 islets from 3 male mice (86.5 ± 10.1 days).

All animal age were provided as mean ± SD.

Supplementary Table 1

	Glucose Ramp			
	No. of mouse	No. of Islets	No. of D-cell ROIs	mean D-cell ROIs/islet ± STD
Male	9	19	124	8.27 ± 3.92
Female	5	33	246	7.45 ± 6.28
	SSTRA+Glucose Ramp			
	No. of mouse	No. of Islets	No. of D-cell ROIs	mean D-cell ROIs/islet ± STD
Male	3	18	70	3.89 ± 2.03
Female	3	19	66	3.47 ± 2.89
	S961+Glucose Ramp			
	No. of mouse	No. of Islets	No. of D-cell ROIs	mean D-cell ROIs/islet ± STD
Male	3	19	55	2.89 ± 2.54
Female	3	19	53	2.79 ± 2.18
	SSTRA+S961+Glucose Ramp			
	No. of mouse	No. of Islets	No. of D-cell ROIs	mean D-cell ROIs/islet ± STD
Male	3	19	124	8.27 ± 3.92
Female	3	19	90	5.29 ± 2.69

Supplementary Table 1. Summary of δ-cell numbers identified under each experimental condition. Shown are the number of mice (column 1), islets (column 2), δ-cell regions of interest (ROIs; column 3), and the mean number of δ-cell ROIs per islet for experiments performed under glucose ramp conditions alone or in the presence of SSTR antagonists (SSTRA), S961, or their combination.
