# Supplementary material for: Pancreatic α-cells are functionally heterogeneous and sex-dependently regulated by neighboring endocrine cells
Source: Commun Biol. 2026 Jun 23;9:1324. doi: 10.1038/s42003-026-10163-6 (PMC13655181; doi:10.1038/s42003-026-10163-6)
Supplement: Supplementary file 3 [file 42003_2026_10163_MOESM3_ESM.pdf]

Description of Additional Supplementary Files:

File name: Supplementary Data 1

Description: The numerical source data for all graphs in the manuscript can be found in Supplementary data 1
